# Comparison of the two up-to-date sequencing technologies for genome assembly: HiFi reads of Pacific Biosciences Sequel II system and ultralong reads of Oxford Nanopore

**DOI:** 10.1093/gigascience/giaa123

**Published:** 2020-12-15

**Authors:** Dandan Lang, Shilai Zhang, Pingping Ren, Fan Liang, Zongyi Sun, Guanliang Meng, Yuntao Tan, Xiaokang Li, Qihua Lai, Lingling Han, Depeng Wang, Fengyi Hu, Wen Wang, Shanlin Liu

**Affiliations:** GrandOmics Biosciences, No.1, East Nengyuan Road, Beijing 102200, China; State Key Laboratory for Conservation and Utilization of Bio-Resources in Yunnan, Research Center for Perennial Rice Engineering and Technology of Yunnan, School of Agriculture, Yunnan University, No.2, North Cuihu Road, Kunming, Yunnan 650091, China; GrandOmics Biosciences, No.1, East Nengyuan Road, Beijing 102200, China; GrandOmics Biosciences, No.1, East Nengyuan Road, Beijing 102200, China; GrandOmics Biosciences, No.1, East Nengyuan Road, Beijing 102200, China; GrandOmics Biosciences, No.1, East Nengyuan Road, Beijing 102200, China; GrandOmics Biosciences, No.1, East Nengyuan Road, Beijing 102200, China; GrandOmics Biosciences, No.1, East Nengyuan Road, Beijing 102200, China; GrandOmics Biosciences, No.1, East Nengyuan Road, Beijing 102200, China; GrandOmics Biosciences, No.1, East Nengyuan Road, Beijing 102200, China; GrandOmics Biosciences, No.1, East Nengyuan Road, Beijing 102200, China; State Key Laboratory for Conservation and Utilization of Bio-Resources in Yunnan, Research Center for Perennial Rice Engineering and Technology of Yunnan, School of Agriculture, Yunnan University, No.2, North Cuihu Road, Kunming, Yunnan 650091, China; State Key Laboratory of Genetic Resources and Evolution, Kunming Institute of Zoology, Chinese Academy of Sciences, No.32, East Jiaochang Road, Kunming, Yunnan 650223, China; Center for Ecological and Environmental Sciences, Key Laboratory for Space Bioscience & Biotechnology, Northwestern Polytechnical University, No.127, West Youyi Road, Xi'an, Shanxi 710072, China; GrandOmics Biosciences, No.1, East Nengyuan Road, Beijing 102200, China; Department of Entomology, College of Plant Protection, China Agricultural University, No.2, West Yuanmingyuan Road, Beijing 100193, China

**Keywords:** assembly comparison, ONT ultralong, PacBio HiFi, CCS, single-molecular sequencer, contiguity

## Abstract

**Background:**

The availability of reference genomes has revolutionized the study of biology. Multiple competing technologies have been developed to improve the quality and robustness of genome assemblies during the past decade. The 2 widely used long-read sequencing providers—Pacific Biosciences (PacBio) and Oxford Nanopore Technologies (ONT)—have recently updated their platforms: PacBio enables high-throughput HiFi reads with base-level resolution of >99%, and ONT generated reads as long as 2 Mb. We applied the 2 up-to-date platforms to a single rice individual and then compared the 2 assemblies to investigate the advantages and limitations of each.

**Results:**

The results showed that ONT ultralong reads delivered higher contiguity, producing a total of 18 contigs of which 10 were assembled into a single chromosome compared to 394 contigs and 3 chromosome-level contigs for the PacBio assembly. The ONT ultralong reads also prevented assembly errors caused by long repetitive regions, for which we observed a total of 44 genes of false redundancies and 10 genes of false losses in the PacBio assembly, leading to over- or underestimation of the gene families in those long repetitive regions. We also noted that the PacBio HiFi reads generated assemblies with considerably fewer errors at the level of single nucleotides and small insertions and deletions than those of the ONT assembly, which generated an average 1.06 errors per kb and finally engendered 1,475 incorrect gene annotations via altered or truncated protein predictions.

**Conclusions:**

It shows that both PacBio HiFi reads and ONT ultralong reads had their own merits. Further genome reference constructions could leverage both techniques to lessen the impact of assembly errors and subsequent annotation mistakes rooted in each.

## Background

The availability of reference genomes has revolutionized the study of biology. The high-quality human reference genome enabled the identification of disease causative alleles [1, 2]; the genomes of agricultural crops have tremendously accelerated our understanding of how artificial selection shaped plant traits and how, in turn, these plant traits may influence species interactions, e.g., phytophagous insects, in agriculture [3, 4]. During the past decade, multiple competing technologies have been developed to improve the quality and robustness of genome assemblies [5–8], enabling genome reference collecting of the tree of life [9–11]. To date, a large number of genomes have been assembled by third-generation sequencing technologies, which can produce individual reads in the range of 10–100 kb or even longer [12–15]. Although the long-read methods still have a high error rate, they have been improving owing to advances in sequencing chemistry and computational tools. For example, the Pacific Biosciences (PacBio) single-molecule real-time (SMRT) sequencing platform released the Sequel II system. The updated SMRT cell enabled high-throughput HiFi reads using the circular consensus sequencing (CCS) mode to provide base-level resolution with >99% single-molecule read accuracy [16]; while Oxford Nanopore Technologies (ONT) launched its PromethION platform, which can yield >7 Tb per run, and its ultralong sequencing application facilitates the achievement of complete genome—telomere to telomere (T2T)—by resolving long and complex repetitive regions for various species including *Homo sapiens* [17]. The 2 cutting-edge sequencing technologies have enabled the sequencing of many species; however, almost all chose a single sequencing system, either the PacBio or the ONT platform, to obtain their reference genomes [15, 18, 19]. Here we present 1 rice individual (*Oryza sativa* ssp. *indica*, 2n = 2x = 24, variety 9311) [20, 21] that was sequenced and assembled independently using the 2 up-to-date systems, and we compare the 2 assemblies to investigate the advantages and limitations of each.

## Findings

**Figure 1:**
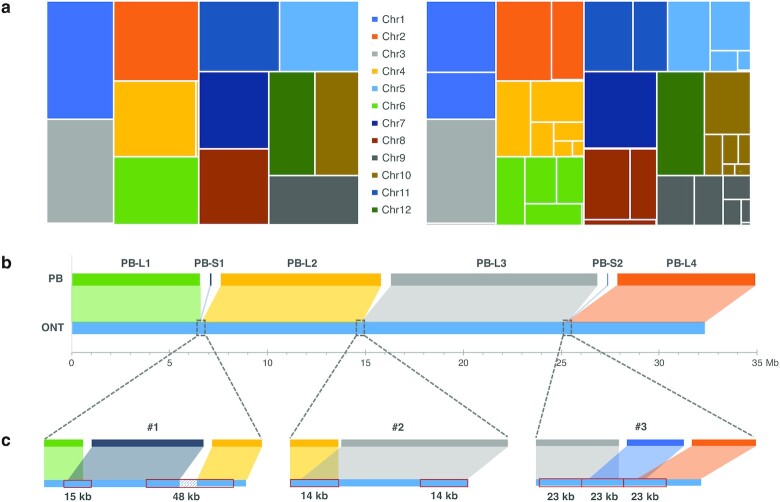
Contiguity of the ONT and PacBio assemblies. (a) Treemaps for contig length difference between the ONT (left) and PacBio (right) assembly; (b) the 6 PacBio contigs mapped to 1 ONT contig corresponding to Chr. 6; (c) details of the 3 PacBio gaps. Red rectangles indicate repeat elements.

Following DNA extraction from the rice sample, we sequenced the 2 extracts using the ONT PromethION and PacBio Sequel II platforms, respectively. The PromethION generated a total of 92 Gb data (230×) with an N50 of 41,473 bp, and the Sequel II produced a total of 253 Gb data (632×) with each molecular fragment being sequenced 14.72 times on average and produced ∼20 Gb HiFi reads (50×) with an average length of 13,363 bp. We applied multiple software packages, including Canu1.9 [22], NextDenovo2.0-beta.1 [23], WTDBG2.5 [24], Flye2.7.1 [25], SHASTA-0.4.0 [26], and NECAT [27], to assemble the rice genome for both the ONT and PacBio dataset (Supplementary Table S1) and then selected the optimal assembly for each sequencing platform on the basis of contig N50 (Supplementary Table S2). The ONT assembly showed higher contiguity, with a contig number of 18 and an N50 value of ∼32 Mb, in comparison with a contig number of 394 and N50 of 17 Mb for the PacBio assembly (Fig. 1a). Ten and 3 of the total 12 autosomes were assembled into a single contig in the ONT and PacBio assembly, respectively. We identified telomeres and centromeres for both assemblies and found that 7 of them reached a T2T-level assembly with no gaps and no Ns in between (Supplementary Table S3). A genome completeness assessment using BUSCO v3.1.0 [28] finds that both assemblies performed well, with the ONT showing a tiny bit better performance (98.62% vs 98.33%, Supplementary Table S4). We mapped both assemblies to a high-quality rice (R498) genome reference [20] using Minimap2 [29]. Both assemblies showed good collinearity (Supplementary Fig. S1), and the PacBio assembly contained more gaps than that of ONT (Fig. 1a).

**Figure 2:**
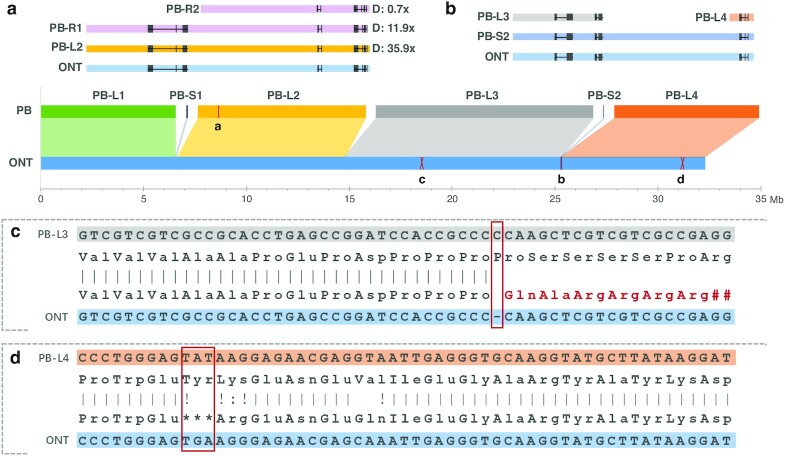
Assembly errors in which genes can be annotated. (a) An example shows gene gains caused by assembly redundancies, of which PB-R1 and PB-R2 had a similarity level of 99.67% and 99.51%, respectively, compared with the corresponding region on PB-L2. D: depth. (b) The gene redundancies caused by gaps that failed to be correctly connected by the PacBio assembly. (c) An example shows how a 1-base deletion led to a frameshift mistake for protein translation. (d) An example shows how a single-base error led to stop codon gain and truncated protein translation.

We then randomly took 1 chromosome (Chr. 6) where ONT's 1 single contig (32,367,127 bp) corresponded to 9 contigs (32,476,323 bp) of the PacBio assembly to investigate and visualize the incongruencies between them. For the 9 PacBio contigs assembled for Chr. 6, 4 reached a length ≥6 Mb and 5 had a length of merely 10–70 kb. We investigated the 3 gaps where the top 4 PacBio contigs (named PB-L1, PB-L2, PB-L3, and PB-L4 from 5′ to 3′ end, respectively) failed to connect (Fig. 1b). We mapped the ONT ultralong reads to those gaps and confirmed their correctness through manual inspections by IGV plot [30] (Supplementary Fig. S2). The Gap 1 between PB-L1 and PB-L2 reached a length of 74,888 bp. One of the short PacBio contigs (PB-S1, length of 70,208 bp) had an overlap of ∼10 kb with the 3′ end of PB-L1, thus leaving Gap 1 a region of 15,722 bp that PacBio failed to cover (Fig. 1c). We further examined the sequences obtained by ONT in and flanking this gap. We found that the overlapping and gap regions represented 2 elements of 15 and 48 kb in length that, although having only 1 copy on Chr. 6, had duplications that could be found on Chr. 5 (Supplementary Fig. S3). Repetitive elements with such lengths exceed the typical length generated by PacBio CCS; therefore the right path can hardly be disentangled from complicated string graphs [22, 31]. The Gap 2 between PB-L2 and PB-L3 characterized a region spanning up to 48 kb on the ONT assembly and is flanked by 2 tandem repeats of 14 kb in length. It was spanned by multiple ONT long reads (Supplementary Fig. S2) and thus can be successfully connected by the ONT assembly. The last gap, between PB-L3 and PB-L4, can be connected by 1 short PacBio contig (PB-S2, 25,292 bp), which had 9,469 and 2,621 bp overlaps with the 3′ end of PB-L3 and 5′ end of PB-L4, respectively. And it showed the same case as Gap 2, containing 3 tandem duplicates of length 23 kb that failed to be connected by PacBio HiFi reads. We found a total of 107 kb redundancies and 15 kb gaps on Chr. 6 owing to PacBio's incorrect assembly, which corresponded to an excess of 13 annotated genes (Fig. 2, Supplementary Table S5). The genome-wide misassembled regions accumulated to a length of ∼668 kb (534 kb redundancies and 134 kb gaps), hosting 54 annotated genes (44 redundancies and 10 loss, Supplementary Table S5). Because the PacBio assembly did not generate any single contigs that ONT broke into multiple segments, we cannot find a counter case for comparison. In addition, a down-sampling test showed that the ONT dataset, unlike the PacBio data, can produce genome assemblies of the same contiguity level using half or one-third of the raw reads, corroborating the central role that ultralong reads played in assembling genome regions with long repeats (Supplementary Fig. S4 and Table S6). It is also worth noting that PacBio can run in long-read mode [32], which, although it can hardly generate reads as long as the ONT ultralong reads, can aid in connecting some of the gaps caused by long repeats. Besides, longer PacBio libraries with HiFi reads reaching 20 kb [33] would be conducive to assembly contiguity as well.

In addition to those gaps that PacBio failed to connect, we noticed that there were a bunch of small-scale mismatches (<85 bp) between the 2 assemblies. First, we extracted the reciprocal matches ≥1 Mb between the 2 assemblies for comparison using QUAST [34]. Then, we mapped the PacBio HiFi reads to both genome assemblies to identify single-nucleotide variants (SNVs) and InDels under the assumption that HiFi reads provide high-level single-base accuracy. The mapping showed that the ONT assembly, although polished using 70× Illumina shotgun reads, still contained a large number of errors. In total, we found 210,993 single-nucleotide errors and 211,517 InDels (mean: 1.39 bp, Supplementary Fig. S5) accounting for an average number of 1.06 errors per kb. However, instead of scattering evenly on the assembly, those errors formed clusters (Supplementary Fig. S6). A further investigation into those regions showed that ∼94% of them have a shotgun read coverage ≤5, which explains why the last polishing step failed to fix those errors (Supplementary Fig. S7a). As those regions were well covered by ONT long reads (Supplementary Fig. S7b), we examined their GC content and methylation profiles, speculating that different methylation patterns in such regions may have reduced the base-calling accuracy there. The results showed that those ONT error-enriched regions contained higher or lower GC content and significantly higher methylation level compared with other genome regions (Supplementary Fig. S8), hence providing a training set that includes information about modifications and sequence motifs of rice where neural network base-calling tools could to some extent alleviate the error rate of the ONT assembly [35]. We also found that 7.48% of those errors were located on exons and affected the ability of ∼2,415 exons (1,475 genes) to translate correctly to amino acid sequences on the ONT genome assembly. Most of those affected genes have multiple paralogous copies on the genome (Supplementary Fig. S9), rather than being single-copy orthologs used in the BUSCO analysis, suggesting a limited performance of short-read–based genome-polishing methods for duplicated genes on the genome. In addition, we did note that HiFi read errors may be enriched in sequences with particular characteristics, rather than being completely random, e.g., regions such as simple sequence repeats and long homopolymers (Supplementary Methods, Fig. S10), which may exacerbate the aforementioned error statistics for the ONT assembly. In addition, QUAST also reported some mismatches >85 bp between the 2 assemblies. A manual examination for several randomly selected discrepancies on Chr. 6 showed that they were repeated regions incorrectly assembled using PacBio reads, or regions with high methylation level where ONT errors were enriched (Supplementary Methods and Fig. S11).

**Figure 3:**
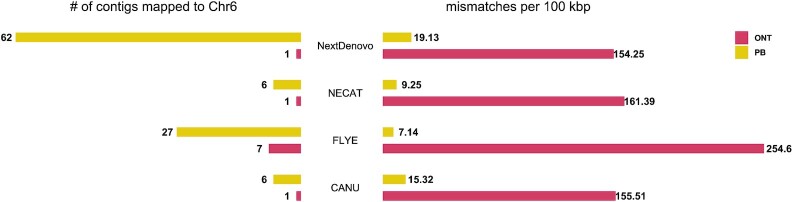
Assembly comparisons using the same methods. Left: number of contigs that were mapped onto Chr. 6; right: number of mismatches (including SNVs and InDels) per 100 kb.

Instead of using the assemblies generated by 2 different methods (Canu vs NextDenovo), a further examination for the 2 sequencing techniques using the same assembly methods (Supplementary Methods) achieved similar results: all assemblers produced a more contiguous genome assembly but with a loss of accuracy using the ONT ultralong reads compared with that using the PacBio HiFi reads (Fig. 3 and Supplementary Fig. S12).

In conclusion, our study investigated genome assembly qualities between the 2 up-to-date competing long-read sequencing techniques—PacBio HiFi reads and ONT ultralong reads. It showed both techniques had their own merits: (i) ONT ultralong reads delivered higher contiguity and prevented false redundancies caused by long repeats, which, in our case of the rice genome, assembled 10 of the 12 autosomes into 1 single contig; and (ii) PacBio HiFi reads produced fewer errors at the level of single nucleotides and small InDels and obtained >1,400 genes that were incorrectly annotated in the ONT assembly owing to its error-prone reads. However, the present study has several limitations, including, among others, (i) NextDenovo, which generated the most contiguous assembly for the ONT reads, is a newly developed assembler whose performance has not been validated on other species; (ii) rice, which has a relatively small and simple genome, cannot characterize the full spectrum of the strengths and weaknesses of the 2 sequencing technologies. Genome studies, especially for large and complex genomes, will shed more light on this matter. Therefore, we suggest that further genome reference constructions leverage both techniques to lessen the impact of assembly errors and subsequent annotation mistakes rooted in each. There is also an urgent demand for improved assembly and error correction algorithms to fulfill this task.

## Methods

### Sample preparation and sequencing

The DNA samples used for ONT and PacBio Sequel II platform sequencing were isolated from leaf tissues using the sodium dodecyl sulfate method and Q13323kit (QIAGEN, Hilden, North Rhine-Westphalia, Germany), respectively (Supplementary Methods). The ONT platform generated a total of 6,100,295 pass reads with an average quality of 8.99 within 20 hours, and the PacBio Sequel II platform generated a total of 21,986,306 subreads with each molecular fragment being sequenced 14.72 times on average within 30 hours. Then, the PacBio subreads were converted to HiFi reads using CCS [36] with default parameters. Additionally, we generated a total of 188,590,034 shotgun reads (∼70×) using a strategy of pair-end 150 bp (PE 150) on the MGISEQ-2000 platform.

### Genome assembly and polishing

After the genome assembly (Supplementary Table S1), we mapped the ONT raw reads and PacBio HiFi reads onto their corresponding genomes using Minimap2 [29] and conducted genome polishing using RACON (Racon, RRID:SCR_017642) [37] through 3 iterations. Then, for the ONT assembly we applied Medaka, a tool designed for ONT error correction, to conduct genome polishing once more. After that, NextPolish1.1.0 [38] was applied to fix small-scale errors (SNVs and InDels) for the ONT assembly using shotgun reads. We did not apply the shotgun-read–based polishing step to the PacBio assembly because PacBio HiFi reads have already reached an accuracy rate of 99%, which is as high as that of the shotgun reads. Finally, the ONT assembly generated by NextDenovo and PacBio assembly generated by Canu (Canu, RRID:SCR_015880) were selected out on the basis of N50 value (Supplementary Table S2) and used for the following comparison analyses.

### Identification for centromeres and telomeres

We identified centromere- and telomere-related sequences using the RCS2 family repeats and 5′-AAACCCT-3′ repeats, respectively [20, 39]. For centromeres, we first aligned the sequences of the RCS2 family (AF058902.1) onto both the ONT and PacBio assemblies using BWA-MEM (BWA, RRID:SCR_010910) [40], and regions that contained full RCS2 family units were identified as centromeres. Telomeres were identified by searching for 5′-AAACCCT-3′ repeats on each contig using Tandem Repeats Finder with default parameters [41].

### Assembly comparison

**Collinearity:** We aligned both assemblies to a high-quality rice genome (variety R498, Accession ID: GCA_002151415.1) using minimap2 [29] with a parameter setting of -x asm5. Then, we visualized the collinearity between the reference and query genomes using dotPlotly [42] (-t, -l, -m 30 000, -q 1 000 000).
**Gap identification:** We aligned the PacBio assembly onto the ONT assembly using minimap2 [29] (-x asm5) and kept the primary hit for each contig. Then, we examined the alignment boundaries for each contig and identified the corresponding gap positions for each contig.
**Identification of mismatches between ONT and PacBio assemblies:** We extracted the reciprocal matches ≥1 Mb between the 2 assemblies for comparison using QUAST 5.0.2 (QUAST, RRID:SCR_001228) with default parameters [34]. QUAST categorized mismatches into 2 different types: local mismatches >85 bp and small-scale mismatches including SNVs and small InDels.
**Identification of errors in forms of single nucleotides and small Indels:** We aligned PacBio HiFi reads onto the ONT assembly and then identified single-nucleotide polymorphisms (SNPs) and InDels using GATK4 (GATK, RRID:SCR_001876) [43] with filtering parameters as follows: QD < 2.0 || MQ < 40.0 || FS > 60.0 || SOR > 3.0 || MQRankSum < -12.5 || ReadPosRankSum < -8.0 for SNPs, and QD < 2.0 || FS > 200.0 || SOR > 10.0 || MQRankSum < -12.5 || ReadPosRankSum < -8.0 for InDels. Given that both the PacBio and ONT assemblies contain 1 set of the paired chromosomes and the discrepancies between them can present the heterozygous sites in the genome, we removed those that were identified to be heterozygous and regarded those homozygous derived alleles (1/1) as ONT errors.
**Gene loss and redundancies:** In the case that multiple PacBio assembly contigs mapped onto the same regions of the ONT assembly, we defined the relatively shorter ones as redundancies conditional on the following 2 criteria: (i) similarity score ≥97% between them; (2) total depth <60 and both have depths <40 (Fig. 2a). In addition, the gaps (shown in Fig. 1) that failed to be covered or were covered twice by the PacBio contigs were defined as losses and redundancies, respectively (Fig. 2b). Finally, those regions that contained genes contributed to the final gene loss and redundancy statistics.
**Incorrect translation caused by ONT errors:** First, we searched for ONT errors that were located on exons on the basis of gene annotations of both the ONT and PacBio assemblies. For the exon inconsistencies between the 2 assemblies (present/absent and mismatches), we aligned amino acid sequences of the PacBio assembly onto corresponding ONT regions using exonerate [44] (–model protein2genome –refine full -n 1) to investigate how the ONT errors affected gene translation.

### DNA methylation

We calculated the genome-wide methylation level for the ONT assembly using Nanopolish v0.11.1 (Nanopolish, RRID:SCR_016157) with called_sites ≥ 10. The methylation profiles and GC content were recorded throughout the genome with a window size of 1,000 bp and a step length of 500 bp. Windows that contained ≥5 ONT errors were defined as ONT error-enriched regions and were used to compare for the methylation and GC content with other genomic regions.

## Data Availability

The raw reads and the genome assemblies of PacBio (assembled using Canu1.9) and ONT (assembled using NextDenvo) are deposited on NCBI under project IDs PRJNA600693, PRJNA644721, and PRJNA644720, respectively. Supporting data, including annotation files, assemblies, and BUSCO results, are also available via the *GigaScience* database, GigaDB [45].

## Additional Files

**Supplementary Methods**.

**Supplementary Figure S1**. Collinearity between genome assembly of rice R498 and that of PacBio (left) and ONT (right). Note: The figure only shows alignments ≥30 kb and query sequences ≥1 Mb.

**Supplementary Figure S2**. IGV plots of the 3 PacBio gaps on Chr. 6. Gray shadows represent gap regions in the PacBio assembly. Red rectangles represent the repeat elements.

**Supplementary Figure S3**. Details of PacBio Gap 1. The 2 repetitive regions matched to another PacBio assembly contig corresponding to Chr5 (PB_Chr5) with high identities. IDY means similarity identities between each other. The bottom panel highlights local IDY values of 100% between each other with an alignment length of 10 kb (PB-L1 vs PB-S1), 12 kb (PB-L1 vs PB_Chr5), and 13 kb (PB-S1 vs PB_Chr5).

**Supplementary Figure S4**. Assembly statistics for the subsampling test. Contig N50 value (upper) and raw read coverage (under) were demonstrated for each assembly. Assemblies applied the same parameters in Supplementary Table S1 for Canu and NextDenovo.

**Supplementary Figure S5**. The length distribution of the ONT InDel errors. Note that InDels of length >20 bp had a total count of 216 and are not shown here.

**Supplementary Figure S6**. Distances between adjacent ONT errors. Those errors tended to cluster in the same region rather than distribute randomly and evenly on the genome, because the distances should have a peak at ∼1,000 bp for an average error rate of 1.06 per kb in the case of random distribution. The yellow curve represents a theoretical error distribution with a mean (SD) distance of 1,000 (200).

**Supplementary Figure S7**. Depth of (a) shotgun reads, (b) ONT raw reads, and (c) PacBio HiFi reads for those ONT error sites. Note that Illumina shotgun read depth >30 had a total count of 10,294 (2.44% of total) and is not shown here.

**Supplementary Figure S8**. Comparison of GC content and methylation level between the ONT error-enriched regions and other regions for the ONT assembly.

**Supplementary Figure S9**. The paralogous copy number distribution of the genes affected by ONT errors. Paralogs were searched using BLAST with e-value cutoff of 1e−5 for each gene.

**Supplementary Figure S10**. Two examples (1 SNP and 1 InDel) that show the mismatches between the ONT and PacBio assemblies, which were well covered by shotgun reads and thus could be errors on HiFi reads generated during the CCS process.

**Supplementary Figure S11**. Examples of the mismatches >85 bp and their corresponding IGV plots for the genome alignments for the PacBio (upper) and ONT (bottom) assemblies. (a) A 1,432-bp InDel where reads mapped onto PacBio's assembly with soft-clips. (b) A 231-bp mismatch on which ONT's assembly displayed a cluster of small-scale errors (GC content: 75.6%, methylation level: 91.0%). (c) A 204-bp InDel (at the end of contig tig00004207) on which no PacBio HiFi reads showed in the alignments (reads mapped onto multiple locations can have a mapping score of zero, and were removed in our analysis). We also noted that this InDel was introduced during the genome-polishing step by Racon, which may corrupt the correctly assembled sequence within repetitive regions.

**Supplementary Figure S12**. Contig alignments of Chr. 6. Red represents contigs that contain InDel mismatches of length ≥85 bp, and green, those that do not. The percentage values represent the coverage ratios (total length of mapped regions/the reference length).

**Supplementary Table S1**. Assembly parameters and computational resource statistics.

**Supplementary Table S2**. Assembly quality evaluation.

**Supplementary Table S3**. The centromeres and telomeres for each chromosome-level contig of ONT and PacBio assemblies.

**Supplementary Table S4**. Results of genome completeness assessment using BUSCO.

**Supplementary Table S5**. Gene loss and redundancies of the PacBio assembly.

**Supplementary Table S6**. Read summary of the subsampling test.

## Abbreviations

bp: base pairs; BUSCO: Benchmarking Universal Single-Copy Orthologs; BWA: Burrows-Wheeler Aligner; CCS: circular consensus sequencing; GATK: Genome Analysis Toolkit; Gb: gigabase pairs; GC: guanine-cytosine; IGV: Integrative Genomics Viewer; kb: kilobase pairs; Mb: megabase pairs; ONT: Oxford Nanopore Technologies; NCBI: National Center for Biotechnology Information; PacBio: Pacific Biosciences; SMRT: single-molecule real-time; SNP: single-nucleotide polymorphism; SNV: single-nucleotide variant; T2T: telomere to telomere; Tb: terabase pairs.

## Competing Interests

D.L., P.R., F.L., Z.S,, G.M., Y.T., X.L., Q.L, L.H., D.W. and S.L. are employees of Grandomics Biosciences, a company that provides bioinformatics and genomics services.

## Authors' Contributions

SL.L., D.W. and W.W. concieved the idea and coordinated the project. S.Z. and W.W. contributed the rice samples. D.L. led the analysis with helps from S.L., P.R., F.L., Z.S,, G.M., Y.T.. X.L., Q.L. and L.H. led the benchwork. S.L. and D.L. formulated the first draft, and all authors contributed to the final version. All authors read and approved the final manuscript. S.L. was supported by Chinese Postdoctoral Science Foundation (2019M660051) and Wuhan Technology Innovation Programme (2020020602012107).

## Supplementary Material

giaa123_GIGA-D-20-00061_Original_Submission

giaa123_GIGA-D-20-00061_Revision_1

giaa123_GIGA-D-20-00061_Revision_2

giaa123_Response_to_Reviewer_Comments_Original_Submission

giaa123_Response_to_Reviewer_Comments_Revision_1

giaa123_Reviewer_1_Report_Original_Submission Jason Chin -- 3/22/2020 Reviewed

giaa123_Reviewer_1_Report_Revision_1 Jason Chin -- 7/14/2020 Reviewed

giaa123_Reviewer_2_Report_Original_Submission Todd Michael -- 4/3/2020 Reviewed

giaa123_Reviewer_2_Report_Revision_1 Todd Michael -- 7/17/2020 Reviewed

giaa123_Reviewer_3_Report_Original_Submission Sergey Nurk -- 4/20/2020 Reviewed

giaa123_Supplemental_Files

## Notes

Dandan Lang and Shilai Zhang contributed equally.
