# Supplementary material for: Comparison of the two up-to-date sequencing technologies for genome assembly: HiFi reads of Pacific Biosciences Sequel II system and ultralong reads of Oxford Nanopore
Source: Gigascience. 2020 Dec 15;9(12):giaa123. doi: 10.1093/gigascience/giaa123 (PMC7736813; doi:10.1093/gigascience/giaa123)
Supplement: giaa123_GIGA-D-20-00061_Revision_1 [file giaa123_giga-d-20-00061_revision_1.pdf]

GigaScience

Comparison of the two up-to-date sequencing technologies for genome assembly: HiFi reads of Pacbio Sequel II system and ultralong reads of Oxford Nanopore

--Manuscript Draft--

Manuscript Number:	GIGA-D-20-00061R1
Full Title:	Comparison of the two up-to-date sequencing technologies for genome assembly: HiFi reads of Pacbio Sequel II system and ultralong reads of Oxford Nanopore
Article Type:	Technical Note
Funding Information:	
Abstract:	The availability of reference genomes has revolutionized the study of biology. Multiple competing technologies have been developed to improve the quality and robustness of genome assemblies during the last decade. The two widely-used long-read sequencing providers – Pacbio (PB) and Oxford Nanopore Technologies (ONT) – have recently updated their platforms: PB enables high throughput HiFi reads with base-level resolution with >99% and ONT generated reads as long as 2 Mb. We applied the two up-to-date platforms to one single rice individual and then compared the two assemblies to investigate the advantages and limitations of each. The results showed that ONT ultralong reads delivered higher contiguity producing a total of 18 contigs of which ten were assembled into a single chromosome compared to that of 394 contigs and three chromosome-level contigs for the PB assembly. The ONT ultralong reads also prevented assembly errors caused by long repetitive regions for which we observed a total of 44 genes of false redundancies and ten genes of false losses in the PB assembly leading to over/under-estimation of the gene families in those long repetitive regions. We also noted that the PB HiFi reads generated assemblies with considerably fewer errors at the level of single nucleotide and small InDels than that of the ONT assembly which generated an average 1.06 errors per Kb and finally engendered 1,475 incorrect gene annotations via altered or truncated protein predictions.
Corresponding Author:	Shanlin Liu
Corresponding Author Secondary Information:	
Corresponding Author's Institution:	
Corresponding Author's Secondary Institution:	
First Author:	DanDan Lang
First Author Secondary Information:	
Order of Authors:	DanDan Lang
	Shilai Zhang
	Pingping Ren
	Fan Liang
	Zongyi Sun
	Guanliang Meng
	Yuntao Tan
	Jiang Hu
	Xiaokang Li
	Qihua Lai
	Lingling Han

Powered by Editorial Manager® and ProduXion Manager® from Aries Systems Corporation

	Depeng Wang
	Fengyi Hu
	Wen Wang
	Shanlin Liu
**Order of Authors Secondary Information:**	
**Response to Reviewers:**	Journal: GigaScience<br>Manuscript ID: GIGA-D-20-00061<br>Title: " Comparison of the two up-to-date sequencing technologies for genome assembly: HiFi reads of Pacbio Sequel II system and ultralong reads of Oxford Nanopore"<br>Author(s): DanDan Lang; Shilai Zhang; Pingping Ren; Fan Liang; Zongyi Sun; Guanliang Meng; Yuntao Tan; Jiang Hu; Xiaokang Li; Qihua Lai; Lingling Han; Depeng Wang; Fengyi Hu; Wen Wang; Shanlin Liu<br><br><br>Dear Dr. Hans Zauner,<br>We are very grateful to both the reviewers and the editor for the critical comments and constructive suggestions, which have helped improve our paper considerably.<br>Below we provide our responses to the reviewers' comments in blue. We have incorporated most of the suggested changes as well as additional analyses. The manuscript has now gone through required revision and reorganization, and we sincerely hope that this revision is satisfactory to the reviewers<br><br>Reviewer #1: This manuscript compares the results of genome assemblies from the data of two long-reads sequencing technologies and multiple genome assemblers. It focuses on analyzing the impact of the sequence qualities (read lengths and accuracies) to the contiguity and the accuracy of the assembled contigs.<br><br>While the results agree with the general understanding of how the read lengths and the basecall accuracies affect the final assembly quality, I found the detailed examples comparing the two picked assemblies are interesting. It provides useful insight for understanding the impact of repeats for genome assembly results for researchers. The manuscript is well written and easy to follow to get the points across. Here are a couple points that I hope the authors will be able to address:<br><br>(1) While the rice strain is documented in the manuscript, it will be useful to comment on the polyploidy of this particular strain? The BUSCO results seem to indicate it is a haploid strain, and the readers may be able to check it out from the strain ID. However, the authors should comment on the polyploid to help the readers. It is important to understand how to interpret results according to the known polyploidy.<br><br>> The rice individual (Oryza sativa) we used in this study is the indica cultivar 9311, which is a diploid strain. We noted it at line 70.<br><br>(2) In the paragraph starting with "Following DNA extraction", please refer to the supplementary material about the extraction protocol there.<br><br>> To conform to the journal style, we moved part of the supplementary methods to the main text, which should have solved this problem. Thank you for pointing it out.<br><br>(3) The authors should comment on the time used for sequencing on PromethION and Sequel II, and the computation resources (CPU/wall clock time, memory, cluster setup, etc.) needed for each assembler.<br><br>> It is a good suggestion. We included it at Table S1 in the resubmitted version.<br><br>(4) The IGV view of the ONT reads mapped the PacBio assembly GAP doest show the disagreement of the ONT reads to the ONT contigs. While the high error rates may it messy to see. If such a view hard to see, it still useful to examine if there is some systematic disagreement between the reads and the contigs. I am hoping the authors can comment on whether some systematic errors are visible. Also, will it provide useful

insight if we compare it to PacBio Reads mapping to the ONT contigs?

> The IGV plot aims to demonstrate the GAPs of the PB HiFi assembly can be spanned by several ONT ultra-long reads, and thus explained the reason why such gaps can be assembled using ultra-long reads. Zoom in the IGV plot may show the systematic errors. However, it will as well dismiss our main purpose. Therefore, we would keep it as its current view.

(5) When the authors refer to "string graph," it needs a citation. The term the "string graph" is coined by Gene Meyer for a specific way to construct a graph for genome assembly. Not all assemblers use the same graph construction. The authors should use "assembly graphs" and cite related papers.

>We added the corresponding citation, and algorithms of the software referred to here is based on string graph, so we kept the term “string graph”.

(6) Related to the polyploidy of the strain, the author mentioned "diploid heterozygous states," there is no citation or explanation to help the readers to know what the authors refer to.

>As assembly obtains one single suite of a diploid genome, only one state of those heterozygous sites presents in the assembly results. The differences between the ONT and the PB assembly could be the real conditions in the individual we sequenced. We clarify it at lines 230-231.

(7) The authors mention the errors in ONT assembly are clustered. The authors' explanation is because of low coverage mapping in the polish steps. Are these clusters caused by repeat contents, low accuracy of ONT assembly op particular sequencing contexts? In the caption of Figure S5, the authors write: "the distances should have a peak around 1,000 bp for an average error rate of 1.06 per kb in the case of random distribution." The author should put a theoretical curve or a simulated one on the same plot to show the distribution of a random error model does generate a different distribution.

>Thank you for the suggestion. Reviewer #2 also proposed a similar suggestion. We further investigated the genomic characteristics in and flanking those error regions. It showed that those error-enriched regions were characterized with higher methylation level compared to the other genome regions, and we added it at lines 146-150. We also added a theoretical curve on Figure S6 (Figure S5 in the last version) to better illustrate our point of view. Thanks for this constructive suggestion.

Reviewer #2: In the manuscript entitled, "Comparison of the two up-to-date sequencing technologies for genome assembly: HiFi reads of PacBio Sequel II and ultralong reads of Oxford Nanopore," Lang et al., generate assemblies for a rice variety (9311) using the two different long read sequencing technologies and then compare contiguity and accuracy statistics. The authors conclude that Oxford Nanopore Technologies (ONT) sequencing provides superior contiguity, while Pacific Bioscience (PacBio) provides superior base quality accuracy, and that the two platforms should be leveraged together for reference quality genomes. Overall the manuscript is very concise and well developed. However, there are a couple points that the authors should acknowledge and discuss, which impact the interpretation of their results.

First off, the BioProject PRJNA600693 was not available to assess the assemblies or the raw data. In a manuscript that compares genomes, validating some of the claims is essential, and the data should be available to the reviewer.

>Thank for pointing it out. It is assessable now. In addition, to follow the rule of GigaScience, we have already uploaded the two assembly files, two annotation files, two complete BUSCO output files, two CDS sequence files, two protein translation files and alignment results to the GigaDB server in the process of our first submission. It should be available to reviewers. The access info is as follows:
username = user30
password = LiuSComparison

Powered by Editorial Manager® and ProduXion Manager® from Aries Systems Corporation

FTP server = parrot.genomics.cn

The authors set up a very nice and simple contrast between PacBio HiFi and ONT. There are some significant differences between the datasets that should be discussed though. The read N50 length of the two platforms is considerably different at 41 kb vs. 13 kb for ONT and PacBio respectively. Moreover the absolute coverage is significantly different between ONT and PacBio at 92 Gb (230x) vs. 253 Gb (632x) respectively, even though the reported HiFi coverage is only 50X. There are several opportunities here. First, the authors should at the very least mention these differences, which at face value explain ONT being more contiguous and PacBio having higher base quality. Second, since the authors have an extraordinary amount of data for this rice line, it would be also interesting to see where the quality or contiguity starts to decrease as a function of the amount or type of data.

>Good point. We clarified the coverage differences between the two platforms in the resubmitted version at lines 77-81. We also subsampled the raw reads to investigate the influence of data size on genome assembly, please find it at lines 127-130 and in Table S6 and Figure S4.

The section about the nucleotide variation is a little confusing. It is stated that the regions (~94%) that showed low base quality in the ONT assembly also had low shotgun read coverage. Was this ONT, PacBio or Illumina coverage that was low? With the amount of coverage that was generated for each platform (ONT, 230x; PacBio, 632x; Illumina 70x) why would there be regions in the assembly with less than 5x coverage. This needs to be clearer. In the same section, SNPs and INDELs are referred to as small-scale mis-assemblies; more accurately these are sequence errors not mis-assemblies. Did the authors use the ONT or PacBio data to look at DNA methylation? If the errors are clustering in the genome then maybe the errors in the ONT sequence are the result of mis-called bases that are highly methylated. Since the data is available this would be an important point to make or reason to rule out.

>It is a very good point regarding to the abnormal coverage issues. Firstly, we clarify that the low coverage refers to the shotgun reads generated using MGI-SEQ platform. Then, we added possible reasons that deterred the correct mapping of short reads for those regions, please find them at lines 146-156.
For the word “mis-assembly”, we agree that those SNVs and InDels should come from sequence errors. We clarified it at line 140.
It is a good suggestion as for the DNA methylation analysis. We investigated the correlation between methylation profiles and those error-enriched regions. It showed that the GC content and methylation level of those error-enriched regions are significantly higher than that of other genome regions. We included it at lines 150-156 and Figure S8.

PacBio can also run in long read mode, so researchers could mix HiFi with longreads on one platform. This would be good to also mention.

>Added, at lines 128-132.

The BUSCO scores for the two genomes are almost identical. It would be good to add a bit of commentary why you see similar BUSCO scores but some differences in protein content. This will help the reader understand the differences and limitations of each measure.

>Thank you. We included the explanation at lines 153-156.

While mentioning exact costs for both methods would not stand the test of time it would be good for the reader to understand the relative cost differences between the two approaches.

>Since the yield of both the platforms (especially the ONT) varies a lot between different species. For example, some human DNA samples can generate > 100 Gb data using one PromethION cell, but some marine or insect species can only generate < 20 Gb data per cell. As a result, we don’t think cost of the current work (both platforms have spent around $4,000 for sequencing) reflects a real cost difference for

Powered by Editorial Manager® and ProduXion Manager® from Aries Systems Corporation

other species. It would be better for the readers to consult their local dealers for the cost details.

Minor points:
What species of rice is 9311? The authors should use the scientific name somewhere in the manuscript to clarify what species is "rice."

>We corrected it as “one rice individual (Oryza sativa indica, 2n = 2x = 24, variety 9311)” at line 69.

Grammar:
The first sentence of the Main Text. Diseases don't find causative alleles. Maybe, "The human reference genome enabled the identification of disease causative alleles…."

Sentence 4 page 3: species don't leverage cutting edge sequencing. "The two cutting edge sequencing technologies has enabled the sequencing of many species…"

Bottom page 4 "It was gone through by multiple ONT long reads…" It was spanned by….

>Thank you for noticing those errors. We have them corrected accordingly.

Reviewer #3: Advances in sequencing technologies provide us with an unprecedented opportunity for high-quality de novo reconstruction of complex eukaryotic genomes. The manuscript presents the comparative analysis of the two assemblies of a rice genome, obtained with ultra-long ONT and Pacbio HiFi sequencing.

First, while a combination of HiFi and ultra-long ONT datasets is available for several human genomes (and maybe some other organisms), the scope of the study is limited to a single organism with a relatively small and simple genome. Moreover, only a single genome has been considered with a single dataset for each technology. In particular, while longer Pacbio HiFi libraries with reads reaching 20Kb are now not uncommon the dataset considered in the study had an average read length less than 12Kb.

>Firstly, human genome, as well as model species, could be special cases. For instance, scientists who work in the field of human health could account for more than half of the entire academic world. They depend heavily on one single genome reference and have been spending tremendous time and money to achieve high-quality genome references, and thus combined as many cutting-edge technologies as possible. However, the vast majority of scientists who study non-model species obtained the genome references of targeted species using only one single sequencing tech, either PB or ONT, due to limited funding. The current work provides scientists valuable information on the pros and cons of PB HiFi and ONT ultra-long, and thus help them decide which one fits their project better, and they can as well learn the disadvantages of their choices in advance, as a results, be cautious to any related conclusions.
>For the library size, more and more studies begin to build long CCS libraries (15 kb – 20 kb) nowadays. We started this work right after the launching of PB sequel II. 10 kb library was recommended to guarantee high accuracy level for each CCS read at that time. We have an average HiFi read length of 13.36 kb, instead of what you mentioned: less than 12 kb which is the average length of subreads. We removed this confusing statement in the main text. In addition, we also added a note in the manuscript clarify this problem saying that “It is also worth noting that PB can run in long read mode, which, although can hardly generate reads as long as the ONT ultralong reads, can aid in connecting some of the gaps caused by long repeats. Besides, longer PB libraries with HiFi reads reaching 20 kb would be conducive to assembly contiguity as well”.

Further I will focus on major issues of the presented analysis and mention some of the minor ones in the end.

Major issues

The 'primary' ONT assembly used was produced by a software tool for which I was not able to find neither publication/white-paper, nor a comprehensive benchmark. Moreover its github page states "In addition, we found that NextDenovo, of the current version, might produce a small number of unexpected connection errors in the highly repetitive regions, which, however, can be easily corrected using additional Hi-C or Bionano data. We are still in a progress of optimizing NextDenovo and will continuously update it, especially in terms of assembly accuracy". Since the only criteria used to choose the 'optimal' assembly between different assembly tools was based on their N50 values, it immediately raises questions about the reliability of the results!
The only confirmation of assembly accuracy given is the dotplot against the reference genome. Unfortunately at the presented resolution (of both the figure and the analysis itself) it fails to convince the reader of the structural accuracy of the assembly.
Also the discrepancy between N50 values of different ONT assemblies looks staggering and raises suspicion. I would suggest to include stats for some other well established long-read assemblers (e.g. Flye and Shasta), which will hopefully be able to produce assembly with continuity comparable to NextDenovo and dispel the suspicion.
As a side note, somehow the main text never states which assemblies were used for the most part of the analysis.

>NextDenovo is publicly available and free for downloading on Github. Up to the time we drafted this response letter, it has more than 2,000 downloads and eight releases (we used version 2.0 for this manuscript and the latest release is version 2.2). It is weird that the reviewer argued about the reliability of its assembly results because it generated a much better results compared to the other software. It is worth noting that its readme text on github states that it performs well especially for ONT ultra-long reads. It means the software developed algorithms to take advantage of ultra-long reads, just like HiCanu designed its algorithms to fit HiFi reads. In addition, HiCanu also showed ca. 10 times higher N50 compared to the other two software. The discrepancy between HiCanu and the other two software for HiFi reads is almost the same to that of NextDenovo for the ONT ultra-long reads (10.38 vs 10.29). As both HiCanu and NextDenovo are publicly available on Github and both have not been certified by peer review, we believe this comment reflect the reviewer’s personal preference.

>Although we think that this comment has more to do with the reviewer’s preference than the actual merit of the manuscript, we added multiple genome assembly results using three more software, FLYE, SHASTA and NECAT, to avoid the staggering N50 differences. In addition to the collinearity analysis for large-scale assembly errors, and SNP and InDels analysis for small-scale assembly errors, we further examined the median size discrepancies between ONT and PB assembly to credit the accuracy of this ONT assembly. We included the results at lines 160-164.

One of the most surprising points of the analysis is that the authors insist on interpreting 'redundancies' as 'misassemblies', which is not a common practice in the assembly benchmarking. While it is important to highlight that while dealing with diploid genomes one can expect to get higher redundancy from HiFi-based assemblies, which should hardly be considered an error as long as they truly represent one of the haplotypes. Besides heterozygous differences, another potential source of redundancies can come from the fact that most long-read assemblers produce overlapping contigs, so the higher the number of fragments the higher will be 'redundancy' from those overlaps. Overall, I don't think that any types of redundancies should be considered as a serious problem at the assembly side. If needed, both types of common redundancies described above can be more-or-less straightforwardly removed post assembly (e.g. purge_dups software), but most importantly they stem primarily from particular algorithm implementation rather than show a deficiency of a data type. For example I would expect Flye's assembly of HiFi data to get much lower redundancy values due to more aggressive settings toward masking heterozygous differences and output of 'bluntified' contigs. Last but not least, from the methodological point of view, while I'm still uncertain how 'redundant' regions were annotated, they have been certainly detected against the draft ONT assembly, which could contain 'collapsed' tandem repeats and other issues, potentially inflating the stats.

Powered by Editorial Manager® and ProduXion Manager® from Aries Systems Corporation

>Objection. We defined those redundancies as mis-assemblies as we intended to assemble one suite of the diploid genome. Practically, the assemblies can be chimeric of the two haploids, rather than containing both haploids in one single assembly file. Most of the current analysis tools are designed to make use of such a genome reference, especially in the field of comparative genomics, which is as well the reason why some software (e.g. purge_dups as you mentioned) are developed to remove those redundancies. For instance, those redundancies could lead to incorrect deductions and conclusions in the analysis for gene expansion and contraction.

>It is worth noting that, instead of generating a perfect genome assembly, we aimed to report our observations objectively based on typical genome assembly pipelines for each sequencing platform, from which the readers can easily find out the advantages and disadvantages of both sequencing platforms and then decide what following analyses should be performed to improve their work. The software developers can also learn directly from the results to improve the corresponding assembly algorithms to avoid those unwanted mis-assemblies.

>The reviewer suggested ONT assemblies could contain 'collapsed' repeats and other issues, so could inflated our estimation. First of all, this argument is intuitive and groundless. Secondly, we defined those redundancies very careful, as what we mentioned in our manuscript, we checked the depths of those potential redundancies and classed them as redundancies only in the case that a total depth < 60X and depth of each < 40X. In addition, we also manually checked several corresponding regions on the ONT assembly to make sure they are spanned by single long read.

Significant part of the main text focuses on the analysis of a handful of particular cases of contig 'breaks' in HiFi assembly. First, the choice of 3 gaps taken for deeper analysis (corresponding to chr6) is not explained and, considering how few of them are described, it is unclear how well they represent the general situation. Second, at least some of the analysis is questionable. For one of the gaps the manuscript states that "... the overlapping and the gap regions represented two elements of 15 kb and 48 kb in length that, although have only one copy on Chr. 6, can find their duplications on Chr. 5 (Figure S3). Repetitive elements with such lengths go beyond the typical length generated by PB CCS, therefore the right path can hardly be disentangled from complicated string graphs." At the same time on Figure S3 the sequence identity for instances of both repeats is reported below 98.5%! Repeat instances of such a high sequence divergence are extremely unlikely to affect HiCanu results, so there must be some other reason for fragmentation of this region.

I would recommend exploring the mapping of the HiFi reads onto the hypothesized genomic sequence, since it has been recently observed that HiFi reads can exhibit depletion of coverage in the GA-rich microsatellite regions of the genome. Besides being responsible for some of the observed gaps in this particular assembly, deeper investigation of this topic could have a serious impact on the choice of technology for certain assembly projects.

>Firstly, the scaffold for comparison was randomly selected and we added it in our manuscript to avoid confusion. Secondly, the three breaks showed in the manuscript are the entire set of breaks possessed in the selected assembly scaffolds for comparison, rather than that we chose the three. We would like to emphasize that we conducted the comparison analysis without any deliberate purpose to take side in any sequencing platform.

>For the sequence identity issues, we reported the average similarity score for the entire repeat regions (IDY of about 98.5%) between ONT assembly and PB assembly. The local similarity score can be up to 100% for regions > 10 kb. We believe those local high similarity regions are to blame for generating those gaps and redundancies. We included the local similarity scores on Figure S3 to avoid confusion.

As a final major note I would like to highlight that the data used in the study doesn't seem to be available yet (query of the PRJNA600693 id doesn't return any results on

NCBI web site). TODO review was hampered by this.

>Thank you for your reminding. It is accessible now. Please find details in our response to Reviewer #2.

Minor issues.

If I understood correctly, the coverage of HiFi data exceeded 500x (253 Gb of data for a roughly 400Mb genome). Since it far exceeds the typical coverage of sequencing projects that most assemblers (e.g. HiCanu) are tuned to, I would suggest to subsample HiFi data or use HiCanu 2.0 (which would perform subsampling automatically) for processing a dataset of such coverage depth.

>We fed Canu self-corrected CCS HiFi reads which has a genome coverage of ca. 50X.

The authors note that "the errors of HiFi reads may be enriched in sequences with particular characteristics, rather than completely random … which may exacerbate the above error statistics for the ONT assembly", suggesting that the rate of the indels in polished ONT assemblies can be noticeably overestimated. I doubt that it is the case though. While the same properties of individual HiFi reads have also been recently observed by other investigators, to the best of my knowledge the consensus quality still tends to be very high. At the same time, the authors can make a much stronger claim by straightforwardly estimating the rate of 'false positive' errors detected within the regions of high coverage of unambiguously mapped Illumina reads.

>Firstly, we did NOT make any strong claims here, we said “may exacerbate the above error statistics for the ONT assembly” instead of what you mentioned “suggesting that the rate of the indels in polished ONT assemblies can be noticeably overestimated”. Secondly, we observed those disagreements between ONT assembly and HiFi assembly, and as what we stated in the manuscript, we also reckon that HiFi reads are of high quality, so we deemed those disagreements (SNPs and InDels) as errors of the ONT assembly. However, as Figure S10 showed, Illumina shotgun reads supported ONT assembly for some those differences and we carefully investigated the subreads of each CCS reads and found out that many subreads also supported the ONT assembly. Such information provided by subreads, however, lost during the CCS process. As it is impossible for us to manually check all such cases, we made a statement that “may exacerbate the above error statistics for the ONT assembly”.

The statement "PB assembly contained more gaps in each chromosome compared to that of ONT" can not be correct, since before that authors say that there were 3 cromosomes fully assembled from HiFi data.

>Corrected.

I would suggest against direct attempts at polishing HiFi assemblies with Racon, since it might result in corrupting the correctly assembled sequence within repetitive regions.

>Racon can correct lots of InDel errors for the HiFi assembly. As a result, we decide to kept it and added a note to remind readers of such an issue in Figure S11.

Conclusion.
Expectedly, while less than 60 genes were affected by identified assembly problems in HiFi assembly (most by redundancies, which as I mentioned before for the most part are easy to mitigate), even after polishing with Illumina reads > 1000 genes were affected by indels in the reported ONT assembly. Setting aside all the above mentioned issues, the results suggest the conclusion that ultra-long ONT could work well for scaffolding HiFi-based assemblies in order to produce almost-perfect genomic reconstruction of inbred rice varieties.

Overall, the presented manuscript falls short of providing the comprehensive comparison of the two technologies for sequence assembly (which a reader expects from its title), but works as a case study of how their combination should be able to provide an almost perfect medium-complexity genome of low-heterozygosity.

Powered by Editorial Manager® and ProduXion Manager® from Aries Systems Corporation

	>As what we replied above, instead of achieving a conclusion of which platform is better and how to obtain a perfect genome assembly, we aimed to report the assembly differences between the two recently released sequencing techniques and provided a reference for those scientists who aim to generate genome references using one of these two sequencing techniques or both. Given the fact that other reviewers found our manuscript to be clear and easy to follow, this comment also seems to reflect the reviewer's personal preference. We did suggest that genome assembly work should leverage both platforms in the next-to-last sentence of our manuscript. However, the reviewer should not draw such a conclusion based on this single sentence, as all the above results talked about comparisons between the two assemblies.<br><br>Last but not least I found some parts of the manuscript quite poorly written. Additional rounds of revisions are highly recommended before resubmission.<br><br>>Thank you for your suggestion. We carefully checked the English writing thorough out the manuscript.
Additional Information:	
Question	Response
Are you submitting this manuscript to a special series or article collection?	No
**Experimental design and statistics**<br><br>Full details of the experimental design and statistical methods used should be given in the Methods section, as detailed in our Minimum Standards Reporting Checklist. Information essential to interpreting the data presented should be made available in the figure legends.<br><br>Have you included all the information requested in your manuscript?	Yes
**Resources**<br><br>A description of all resources used, including antibodies, cell lines, animals and software tools, with enough information to allow them to be uniquely identified, should be included in the Methods section. Authors are strongly encouraged to cite Research Resource Identifiers (RRIDs) for antibodies, model organisms and tools, where possible.<br><br>Have you included the information requested as detailed in our Minimum	Yes

Standards Reporting Checklist?	
**Availability of data and materials**<br><br>All datasets and code on which the conclusions of the paper rely must be either included in your submission or deposited in publicly available repositories (where available and ethically appropriate), referencing such data using a unique identifier in the references and in the "Availability of Data and Materials" section of your manuscript.<br><br>Have you have met the above requirement as detailed in our Minimum Standards Reporting Checklist?	Yes

Powered by Editorial Manager® and ProduXion Manager® from Aries Systems Corporation

Manuscript

Click here to access/download;Manuscript;manuscript-20200702 .pdf

Comparison of the two up-to-date sequencing technologies for genome assembly: HiFi reads of Pacbio Sequel II system and ultralong reads of Oxford Nanopore

Dandan Lang[1#], Shilai Zhang[2#], Pingping Ren[1], Fan Liang[1], Zongyi Sun[1], Guanliang Meng[1], Yuntao Tan[1], Jiang Hu[1], Xiaokang Li, Qihua Lai, Lingling Han[1], Depeng Wang[1], Fengyi Hu[2], Wen Wang[3,4*], Shanlin Liu[1,5*]

1. GrandOmics Biosciences, Beijing, 102200, China
2. State Key laboratory for Conservation and Utilization of Bio-Resources in Yunnan, Research Center for Perennial Rice Engineering and Technology of Yunnan, School of Agriculture, Yunnan University, Kunming, Yunnan, 650091, China
3. State Key Laboratory of Genetic Resources and Evolution, Kunming Institute of Zoology, Chinese Academy of Sciences, 650223 Kunming, Yunnan, China.
4. Center for Ecological and Environmental Sciences, Key Laboratory for Space Bioscience & Biotechnology, Northwestern Polytechnical University, 710072 Xi'an, China.
5. Department of Entomology, College of Plant Protection, China Agricultural University, 100193 Beijing, China

[#]Contribute equally

*Correspondence to Shanlin Liu: liushanlin@grandomics.com & Wen Wang: wwang@mail.kiz.ac.cn

Abstract

The availability of reference genomes has revolutionized the study of biology. Multiple competing technologies have been developed to improve the quality and robustness of genome assemblies during the last decade. The two widely-used long-read sequencing providers – Pacbio (PB) and Oxford Nanopore Technologies (ONT) – have recently updated their platforms: PB enables high throughput HiFi reads with base-level resolution with > 99% and ONT generated reads as long as 2 Mb. We applied the two up-to-date platforms to one single rice individual and then compared the two assemblies to investigate the advantages and limitations of each. The results showed that ONT ultralong reads delivered higher contiguity producing a total of 18 contigs of which ten were assembled into a single chromosome compared to that of 394 contigs and three chromosome-level contigs for the PB assembly. The ONT ultralong reads also prevented assembly errors caused by long repetitive regions for which we observed a total of 44 genes of false redundancies and ten genes of false losses in the PB assembly leading to over/under-estimation of the gene families in those long repetitive regions. We also noted that the PB HiFi reads generated assemblies with considerably fewer errors at the level of single nucleotide and small InDels than that of the ONT assembly which generated an average 1.06 errors per kb and finally engendered 1,475 incorrect gene annotations via altered or truncated protein predictions.

Key words: assembly comparison, ONT ultralong, PB HiFi, CCS, single-molecular sequencer, contiguity

Findings

The availability of reference genomes has revolutionized the study of biology – the high quality human reference genome enabled the identification of disease causative alleles [1,2]; the genomes of agricultural crops have tremendously accelerated our understanding on how artificial selection shaped plant traits and how, in turn, these plant traits may influence species interactions, e.g. phytophagous insects, in agriculture [3,4]. During the last decade, multiple competing technologies have been developed to improve the quality and robustness of genome assemblies [5–8], enabling genome reference collecting of the tree of life [9–11]. To date, a large number of genomes have been assembled by Third Generation Sequencing (TGS) technologies which can produce individual reads in the range of 10~100 kb or even longer [12–15]. Although the long-read still has a high error rate, it has been improving owing to the advances in sequencing chemistry and computational tools, e.g. Pacbio (PB) Single-molecule real-time (SMRT) sequencing platform released the Sequel II system of which the updated SMRT cell enabled high throughput HiFi reads using the circular consensus sequencing (CCS) mode to provide base-level resolution with > 99% single-molecule read accuracy [16]; while the Oxford Nanopore Technologies (ONT) launched its PromethION platform which can yield > 7 Tb per run and its ultralong sequencing application facilitates the achievement of complete genome - Telomere to Telomere (T2T) - by resolving long and complex repetitive regions for various species including *Homo sapien* [17]. The two cutting edge sequencing technologies have enabled the sequencing of many species; however, almost all chose one single sequencing system, either the PB or the ONT platform, to obtain their reference genomes [15,18,19]. Here we present one rice individual (*Oryza sativa* ssp. *indica*, 2n = 2x = 24, variety 9311) [20,21] that was sequenced and assembled independently using the two up-to-date systems, and then we compared the two assemblies to investigate the advantages and limitations of each.

Following DNA extraction from the rice sample, we sequenced the two extracts using ONT PromethION and PB Sequel II platforms, respectively. The PromethION generated a total of 92 Gb data (230X) with an N50 of 41,473 bp, and the Sequel II produced a total of 253 Gb data (632X) with each molecular fragment being sequenced 14.72 times on average and produced ca. 20 Gb HiFi reads (50X) with an average length of 13,363 bp. We applied multiple software, including Canu1.9 [22], NextDenovo2.0-beta.1 (https://github.com/Nextomics/NextDenovo), WTDBG2.5 [23], Flye2.7.1 [24], SHASTA-0.4.0 [25] and NECAT (https://github.com/xiaochuanle/NECAT) to assemble the rice genome for both the ONT and PB dataset (Table S1), and then selected the optimal assembly for each sequencing platform based on contig N50 (Table S2). The ONT assembly showed higher contiguity with a contig number of 18 and an N50 value of ca. 32 Mb in comparison to a contig number of 394 and N50 of 17 Mb for the PB assembly (Figure 1a). Ten and three out of the total 12 autosomes were assembled into a single contig in the ONT and PB assembly, respectively. We identified telomeres and centromeres for both assemblies and found that seven of them reached a T2T level assembly with no gaps and no Ns in between (Table S3). A genome completeness assessment using BUSCOv3.1.0 [26] finds both assemblies performed well with the ONT having a tiny improvement (98.62% vs 98.33%, Table S4). We mapped both assemblies to a high-quality rice (R498) genome reference [20] using Minimap2 [27]. Both assemblies showed good collinearity (Figure S1) and the PB assembly contained more gaps compared to that of ONT (Figure 1a).

We then randomly took one chromosome (Chr. 6) where ONT's one single contig (32,367,127 bp) corresponded to nine contigs (32,476,323 bp) of the PB assembly to investigate and visualize the incongruencies between them. For the nine contigs of PB assembled for the Chr. 6, four reached a length ≥ 6 Mb and five had a length of merely 10-70 kb. We investigated the three gaps where the top four PB contigs (named as PB-L1, PB-L2, PB-L3 and PB-L4 from 5' to 3'end, respectively) failed to connect (Figure 1b). We mapped the ONT ultralong reads to those gaps and confirmed their correctness through manual inspections by IGV plot [28](Figure S2). The gap #1 between PB-L1

and PB-L2 reached a length of 74,888 bp. One of the short PB contigs (PB-S1, length of 70,208 bp) had an overlap of ~10 kb with the 3’ end of PB-L1, thus left the gap #1 a region of 15,722 bp that PB failed to cover (Figure 1c). We further examined the sequences obtained by ONT in and flanking this gap. It showed that the overlapping and the gap regions represented two elements of 15 kb and 48 kb in length that, although have only one copy on Chr. 6, can find their duplications on Chr. 5 (Figure S3). Repetitive elements with such lengths go beyond the typical length generated by PB CCS, therefore the right path can hardly be disentangled from complicated string graphs [22,29]. The gap #2 between PB-L2 and PB-L3 characterized a region spanning up to 48 kb on the ONT assembly and is flanked by two tandem repeats of 14 kb in length. It was spanned by multiple ONT long reads (Figure S2), so can be successfully connected by the ONT assembly. The last gap between PB-L3 and PB-L4 can be connected by one short PB contig (PB-S2, 25,292 bp), which had 9,469 and 2,621 bp overlaps with 3’end of PB-L3 and 5’end of PB-L4, respectively. And it showed the same case as gap #2, containing three tandem duplicates of length 23 kb that failed to be connected by PB HiFi reads. We found a total of 107 kb redundancies and 15 kb gaps on Chr. 6 owing to PB’s incorrect assembly, which corresponded to an excess of 13 annotated genes (Figure 2, Table S5). The genome-wide misassembled regions accumulated to a length of ~ 668 kb (534 kb redundancies and 134 kb gaps), hosting 54 annotated genes (44 redundancies and 10 loss, Table S5). As PB assembly did not generate any single contigs that ONT broke into multiple segments, we cannot find a counter case for comparison. In addition, a down-sampling test showed that the ONT dataset, unlike the PB data, can produce genome assemblies of the same contiguity level using half or one-third of raw reads, corroborating the central role that ultralong reads played in assembling genome regions with long repeats (Figure S4 and Table S6). It is also worth noting that PB can run in long read mode [30], which, although can hardly generate reads as long as the ONT ultralong reads, can aid in connecting some of the gaps caused by long repeats. Besides, longer PB libraries with HiFi reads reaching 20 kb [31] would be conducive to assembly contiguity as well.

In addition to those gaps that PB failed to connect, we noticed that there were a bunch of small-scale mismatches (< 85 bp) between the two assemblies. Firstly, we extracted the reciprocal matches ≥ 1 M between the two assemblies for comparison using QUAST [32]. Then, we mapped the PB HiFi reads to both genome assemblies to identify assembly errors under the assumption that HiFi reads provide high-level single-base accuracy. It showed that the ONT assembly, although polished using 70X Illumina's shotgun reads, still contained a large number of errors. In total, we found 210,993 single nucleotide errors and 211,517 InDels (Mean: 1.39 bp, Figure S5) accounting for an average number of 1.06 errors per kb. However, instead of scattering evenly on the assembly, those errors formed into clusters (Figure S6). A further investigation for those regions showed ~ 94% of them have a shotgun read coverage ≤ 5, which explains why the last polishing step failed to fix those errors (Figure S7a). As those regions were well covered by ONT long reads (Figure S7b), we examined the GC content and methylation profiles for them speculating that different methylation patterns in such regions may have reduced the base calling accuracies there. The results showed that those ONT error-enriched regions contained higher or lower GC content and significantly higher methylation level compared to other genome regions (Figure S8). We also found that 7.48 % of those errors located on exons and affected ~ 2,415 exons (1,475 genes) to translate correctly to amino acid sequences on the ONT genome assembly. Most of those affected genes have multiple paralogous copies on the genome (Figure S9), rather than being single-copy orthologs utilized in the BUSCO analysis, revealing a limited performance of short-reads-based genome polishing methods for duplicated genes on the genome. In addition, we did note that the errors of HiFi reads may be enriched in sequences with particular characteristics, rather than completely random, for example, regions like simple sequence repeats and long homopolymers (Supplementary Methods, Figure S10) which may exacerbate the above error statistics for the ONT assembly. What's more, QUAST also reported some mismatches > 85 bp between the two assemblies. A manual examination for several randomly-selected discrepancies on Chr. 6 showed that they were repeated regions incorrectly assembled

by PB, or regions with high methylation level where ONT errors enriched (Supplementary Methods and Figure S11).

In conclusion, our study investigated genome assembly qualities between the two up-to-date competing long read sequencing techniques - the PB's HiFi reads and the ONT's ultralong reads. It showed both techniques had their own merits with: (1) ONT ultralong reads delivered higher contiguity and prevented false redundancies caused by long repeats, which, in our case of the rice genome, assembled 10 out of the 12 autosomes into one single contig, and (2) PB HiFi reads produced fewer errors at the level of single nucleotide and small InDels and obtained more than 1,400 genes that incorrectly annotated in the ONT assembly due to its error-prone reads. Therefore, we suggest that further genomic studies, especially genome reference constructions, should leverage both techniques to lessen the impact of assembly errors and subsequent annotation mistakes rooted in each. There is also an urgent demand for improved assembly and error correction algorithms to fulfill this task.

Methods

Sample preparation and sequencing

The DNA used for ONT and PB sequel II platform sequencing were isolated from leaf tissues using SDS method and Q13323kit (QIAGEN), respectively (Supplementary Methods). The ONT platform generated a total of 6,100,295 pass reads with an average quality of 8.99 within 20 hours, and the PB sequel II platform generated a total of 21,986,306 subreads with each molecular fragment being sequenced 14.72 times on average within 30 hours. Then, the PB subreads converted to HiFi reads using ccs (https://github.com/PacificBiosciences/ccs) with default parameters. Additionally, we generated a total of 188,590,034 shotgun reads (~70X) using a strategy of pair-end 150 bp (PE 150) on the MGISEQ-2000 platform.

Genome assembly and polishing

After the genome assembly and selection (Table S1 & S2), we mapped the ONT raw reads and PB HiFi reads onto their corresponding genomes using Minimap2 [27] and conducted genome polishing using RACON [33] through three iterations. Then, for the ONT assembly we applied Medaka, a tool designed for ONT error correction, to conduct genome polishing once more. After that, NextPolish1.1.0 [34] was applied to fix small-scale errors (SNVs and InDels) for the ONT assembly using shotgun reads. We did not apply the shotgun-read-based polishing step to the PB assembly, since HiFi reads of PB platform have already reached an accurate rate of 99% as high as that of the shotgun reads.

Identification for Centromeres and Telomeres

We identified centromere and telomere-related sequences using the RCS2 family repeats and 5'-AAACCCT-3' repeats, respectively [20,35]. For centromeres, we first aligned the sequences of RCS2 family (AF058902.1) onto both the ONT and PB assemblies using BWA-MEM [36], and regions that contained full units of RCS2 family were identified as centromeres. Telomeres were identified by searching for 5'-AAACCCT-3' repeats on each contig using Tandem Repeats Finder with default parameters [37].

Assembly comparison

Collinearity: We aligned both assemblies to a high-quality rice genome (variety R498, Accession ID: GCA_002151415.1) using minimap2 [27] with a parameter setting of -x asm5. Then, we visualized the collinearity between the reference and query genomes using dotPlotly (https://github.com/tpoorten/dotPlotly, -t, -l, -m 30000, -q 1000000).

Gap identification: We aligned the PB assembly onto the ONT assembly using minimap2 [27] (-x asm5) and kept the primary hit for each contig. Then, we examined the alignment boundaries for each contig and identified the corresponding gap positions for each contig.

Identification of mismatches between ONT and PB assembly: we extracted the

reciprocal matches ≥ 1 M between the two assemblies for comparison using QUAST 5.0.2 with default parameters [32]. QUAST categorized mismatches into two different types: local mismatches > 85 bp and small-scale mismatches including SNVs and small InDels.

Identification of errors in forms of single nucleotide and small Indels: We aligned PB HiFi reads onto the ONT assembly and then identified SNPs and InDels using GATK4 [38] with filtering parameters: QD < 2.0 || MQ < 40.0 || FS > 60.0 || SOR > 3.0 || MQRankSum < -12.5 || ReadPosRankSum < -8.0 for SNPs, and QD < 2.0 || FS > 200.0 || SOR > 10.0 || MQRankSum < -12.5 || ReadPosRankSum < -8.0 for indels. Given that both the PB and ONT assembly contain one suite of the diploid genome and the discrepancies between them can present the heterozygous sites in the genome, we removed those that were identified to be heterozygous, and regarded those homozygous derived alleles (1/1) as ONT errors.

Gene loss and redundancies: In the case that multiple PB assembly contigs mapped onto the same regions of the ONT assembly, we defined the relatively shorter ones as redundancies conditional on the following two criteria: (1) have a similarity score ≥ 97% between each other; (2) have a total depth < 60 and both have depths < 40 (Figure 2a). In addition, the gaps (showed in Figure 1) failed to be covered or covered twice by the PB contigs were defined as losses and redundancies, respectively (Figure 2b). Finally, those regions that contained genes contributed to the final gene loss and redundancy statistics.

Incorrect translation caused by ONT errors: Firstly, we searched for ONT errors that located on exons based on gene annotations of both the ONT and PB assembly. For the exon inconsistencies between the two assemblies (present/absent and mismatches), we aligned amino acid sequences of the PB assembly onto corresponding ONT regions using exonerate [39] (--model protein2genome --refine full -n 1) to investigate how the ONT errors affected gene translation.

DNA methylation

We calculated the genome-wide methylation level for the ONT assembly using Nanopolish v0.11.1 (https://github.com/jts/nanopolish/) with called_sites ≥10. The methylation profiles and GC content were recorded throughout the genome with a window size of 1,000 bp and a step length of 500 bp. Windows that contains ≥ 5 ONT errors were defined as ONT error-enriched regions and were utilized to compare for the methylation and GC content with other genomic regions.

Availability of data and materials

We have all the data including two genome assemblies and their corresponding raw reads deposited on NCBI under the project ID PRJNA600693.

Competing interests

The authors declare that they have no competing interests.

References

1. Weischenfeldt J, Symmons O, Spitz F, Korbel JO. Phenotypic impact of genomic structural variation: insights from and for human disease. Nat Rev Genet. 2013;14:125–38.

2. Fujimoto A, Furuta M, Totoki Y, Tsunoda T, Kato M, Shiraishi Y, et al. Whole-genome mutational landscape and characterization of noncoding and structural mutations in liver cancer. Nat Genet. 2016;48:500.

3. Saxena RK, Edwards D, Varshney RK. Structural variations in plant genomes. Brief Funct Genomics. 2014;13:296–307.

4. Chen YH, Gols R, Benrey B. Crop domestication and its impact on naturally selected trophic interactions. Annu Rev Entomol. 2015;60:35–58.

5. Wheeler DA, Srinivasan M, Egholm M, Shen Y, Chen L, McGuire A, et al. The complete genome of an individual by massively parallel DNA sequencing. Nature. 2008;452:872–6.

6. Bentley DR, Balasubramanian S, Swerdlow HP, Smith GP, Milton J, Brown CG, et al. Accurate whole human genome sequencing using reversible terminator chemistry. Nature. 2008;456:53–9.

7. Pushkarev D, Neff NF, Quake SR. Single-molecule sequencing of an individual human genome. Nat Biotechnol. 2009;27:847.

8. Rothberg JM, Hinz W, Rearick TM, Schultz J, Mileski W, Davey M, et al. An integrated semiconductor device enabling non-optical genome sequencing. Nature. 2011;475:348–52.

9. Seberg O, Droege G, Barker K, Coddington JA, Funk V, Gostel M, et al. Global Genome Biodiversity Network: saving a blueprint of the Tree of Life–a botanical perspective. Ann Bot. 2016;118:393–9.

10. Mukherjee S, Seshadri R, Varghese NJ, Eloe-Fadrosh EA, Meier-Kolthoff JP, Göker M, et al. 1,003 reference genomes of bacterial and archaeal isolates expand coverage of the tree of life. Nat Biotechnol. 2017;35:676.

11. Lewin HA, Robinson GE, Kress WJ, Baker WJ, Coddington J, Crandall KA, et al. Earth BioGenome Project: sequencing life for the future of life. Proc Natl Acad Sci. 2018;115:4325–33.

12. Chaisson MJP, Huddleston J, Dennis MY, Sudmant PH, Malig M, Hormozdiari F,

et al. Resolving the complexity of the human genome using single-molecule sequencing. Nature. 2015;517:608–11.
13. VanBuren R, Bryant D, Edger PP, Tang H, Burgess D, Challabathula D, et al. Single-molecule sequencing of the desiccation-tolerant grass *Oropetium thomaeum*. Nature. 2015;527:508–11.
14. Gordon D, Huddleston J, Chaisson MJP, Hill CM, Kronenberg ZN, Munson KM, et al. Long-read sequence assembly of the gorilla genome. Science. 2016;352:aae0344.
15. Jiao Y, Peluso P, Shi J, Liang T, Stitzer MC, Wang B, et al. Improved maize reference genome with single-molecule technologies. Nature. 2017;546:524–7.
16. Wenger AM, Peluso P, Rowell WJ, Chang P-C, Hall RJ, Concepcion GT, et al. Accurate circular consensus long-read sequencing improves variant detection and assembly of a human genome. Nat Biotechnol. 2019;37:1155–62.
17. Miga KH, Koren S, Rhie A, Vollger MR, Gershman A, Bzikadze A, et al. Telomere-to-telomere assembly of a complete human X chromosome. bioRxiv. 2019;735928.
18. Loman NJ, Quick J, Simpson JT. A complete bacterial genome assembled *de novo* using only nanopore sequencing data. Nat Methods. 2015;12:733–5.
19. Jain M, Koren S, Miga KH, Quick J, Rand AC, Sasani TA, et al. Nanopore sequencing and assembly of a human genome with ultra-long reads. Nat Biotechnol. 2018;36:338.
20. Du H, Yu Y, Ma Y, Gao Q, Cao Y, Chen Z, et al. Sequencing and *de novo* assembly of a near complete *indica* rice genome. Nat Commun. 2017;8.
21. Yu J, Wang J, Lin W, Li S, Li H, Zhou J, et al. The genomes of *Oryza sativa*: a history of duplications. PLoS Biol. 2005;3.
22. Koren S, Walenz BP, Berlin K, Miller JR, Bergman NH, Phillippy AM. Canu: scalable and accurate long-read assembly via adaptive k-mer weighting and repeat separation. Genome Res. 2017;27:722–36.
23. Ruan J, Li H. Fast and accurate long-read assembly with wtdbg2. Nat Methods. 2020;17:155–8.
24. Kolmogorov M, Yuan J, Lin Y, Pevzner PA. Assembly of long, error-prone reads using repeat graphs. Nat Biotechnol. 2019;37:540–6.
25. Shafin K, Pesout T, Lorig-Roach R, Haukness M, Olsen HE, Bosworth C, et al. Nanopore sequencing and the Shasta toolkit enable efficient *de novo* assembly of eleven human genomes. Nat Biotechnol. 2020;1–10.
26. Seppey M, Manni M, Zdobnov EM. BUSCO: assessing genome assembly and annotation completeness. Gene Predict. 2019;227–45.
27. Li H. Minimap2: pairwise alignment for nucleotide sequences. Bioinformatics. 2018;34:3094–100.
28. Robinson JT, Thorvaldsdóttir H, Winckler W, Guttman M, Lander ES, Getz G, et al. Integrative genomics viewer. Nat Biotechnol. 2011;29:24–6.
29. Myers EW. The fragment assembly string graph. Bioinformatics. 2005;21:79–85.
30. Rhoads A, Au KF. PacBio sequencing and its applications. GPB. 2015;13:278–89.
31. Nurk S, Walenz BP, Rhie A, Vollger MR, Logsdon GA, Grothe R, et al. HiCanu: accurate assembly of segmental duplications, satellites, and allelic variants from high-fidelity long reads. bioRxiv. 2020.

32. Mikheenko A, Prjibelski A, Saveliev V, Antipov D, Gurevich A. Versatile genome assembly evaluation with QUAST-LG. Bioinformatics. 2018;34:i142–50.
33. Vaser R, Sović I, Nagarajan N, Šikić M. Fast and accurate *de novo* genome assembly from long uncorrected reads. Genome Res. 2017;27:737–46.
34. Hu J, Fan J, Sun Z, Liu S. NextPolish: a fast and efficient genome polishing tool for long read assembly. Bioinformatics. 2019.
35. Dong F, Miller JT, Jackson SA, Wang G-L, Ronald PC, Jiang J. Rice (*Oryza sativa*) centromeric regions consist of complex DNA. Proc Natl Acad Sci. 1998;95:8135–40.
36. Li H, Durbin R. Fast and accurate short read alignment with Burrows–Wheeler transform. Bioinformatics. 2009;25:1754–60.
37. Benson G. Tandem repeats finder: a program to analyze DNA sequences. Nucleic Acids Res. 1999;27:573–80.
38. McKenna A, Hanna M, Banks E, Sivachenko A, Cibulskis K, Kernytsky A, et al. The Genome Analysis Toolkit: a MapReduce framework for analyzing next-generation DNA sequencing data. Genome Res. 2010;20:1297–303.
39. Slater GSC, Birney E. Automated generation of heuristics for biological sequence comparison. BMC Bioinformatics. 2005;6:31.

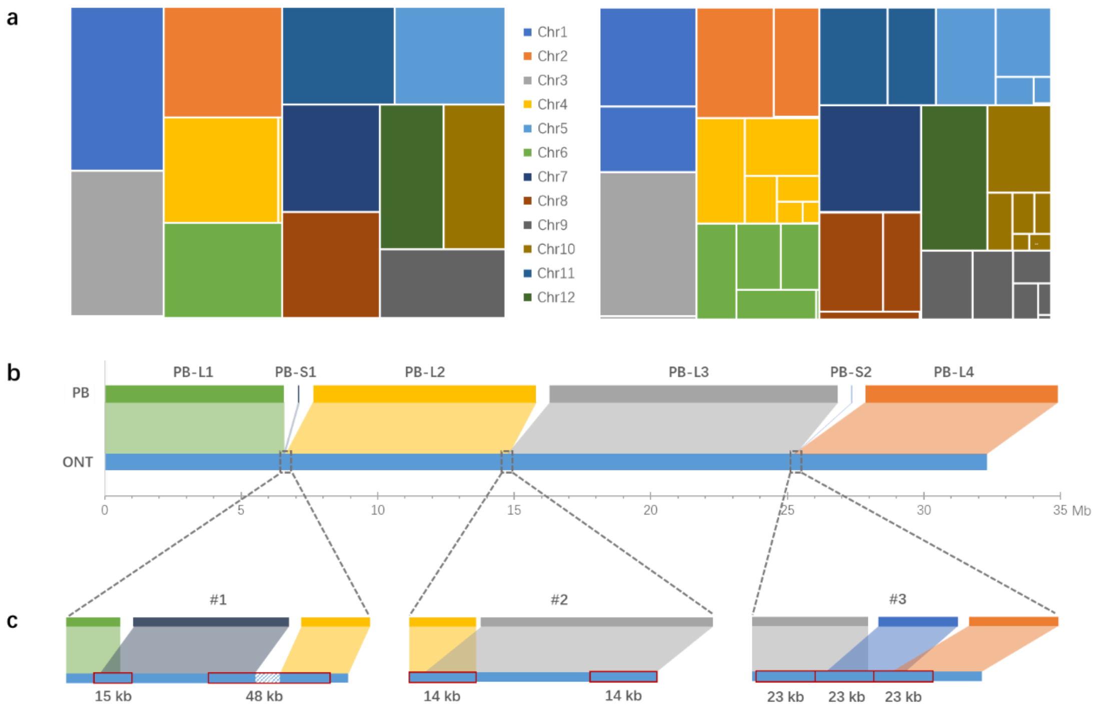

Figure 1. Contiguity of the ONT and PB assemblies. (a) Treemaps for contig length difference between the ONT (left) and PB (right) assembly; (b) The six PB contigs mapped to one ONT contig corresponding to Chr. 6; (c) Details of the three PB gaps. Red rectangles noted the repeat elements.

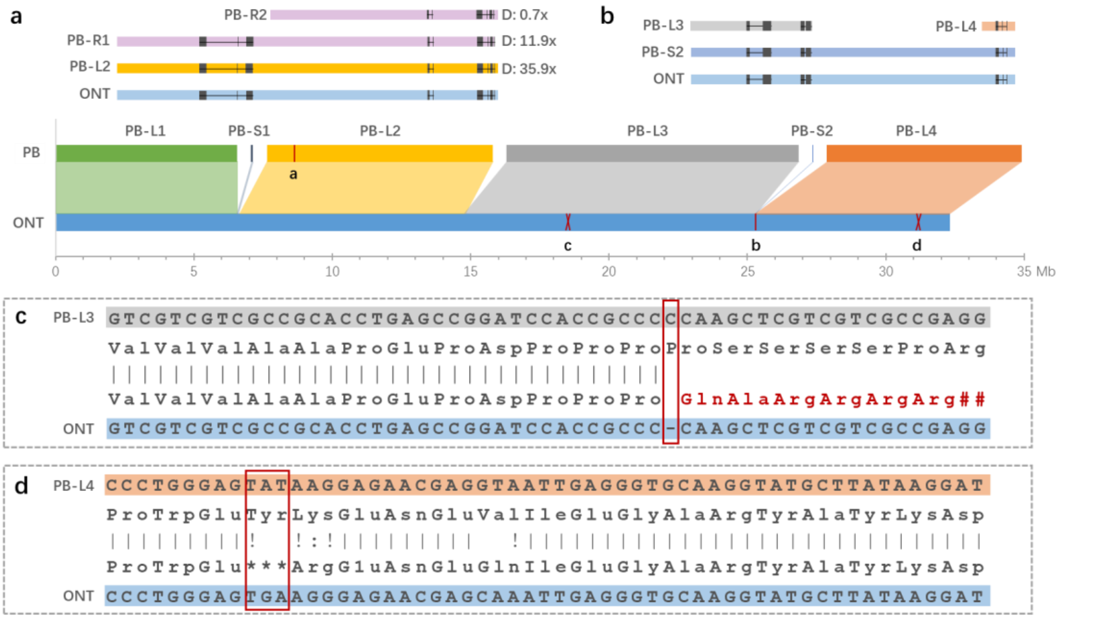

Figure 2. Assembly errors in which genes can be annotated. (a) An example shows gene gains that caused by assembly redundancies, of which the PB-R1 and PB-R2 had a similarity level of 99.67% and 99.51%, respectively, compared to the corresponding region on PB-L2, and "D" abbreviates from depth; (b) The gene redundancies caused by gaps that failed to be correctly connected by the PB assembly; (c) An example shows a 1-base deletion led to frameshift mistake for protein translation; (d) An example shows single base error led to stop codon gain and truncated protein translation.

Click here to access/download
Supplementary Material
Supplementary file-20200702.pdf

FigureS1

Click here to access/download
Supplementary Material
Figure S1.pdf

FigureS3

Click here to access/download
Supplementary Material
Figure S3.pdf

FigureS4

Click here to access/download
Supplementary Material
Figure S5.pdf

FigureS7

Click here to access/download
Supplementary Material
Figure S7.pdf

FigureS9

Click here to access/download
Supplementary Material
Figure S9.pdf

FigureS10

Click here to access/download
Supplementary Material
Figure S10.pdf

FigureS11

Click here to access/download
Supplementary Material
Figure S11.pdf

Figure S2

Click here to access/download
Supplementary Material
Figure S2.svg

Figure S4

Click here to access/download
Supplementary Material
Figure S4.svg

Figure S6

Click here to access/download
Supplementary Material
Figure S6.svg

Figure S8

Click here to access/download
Supplementary Material
Figure S8.svg

Figure 1

Click here to access/download
Supplementary Material
Figure 1.svg

Figure 2

Click here to access/download

Supplementary Material

Figure 2.svg
