# Supplementary material for: Comparison of the two up-to-date sequencing technologies for genome assembly: HiFi reads of Pacific Biosciences Sequel II system and ultralong reads of Oxford Nanopore
Source: Gigascience. 2020 Dec 15;9(12):giaa123. doi: 10.1093/gigascience/giaa123 (PMC7736813; doi:10.1093/gigascience/giaa123)
Supplement: giaa123_GIGA-D-20-00061_Revision_2 [file giaa123_giga-d-20-00061_revision_2.pdf]

GigaScience

Comparison of the two up-to-date sequencing technologies for genome assembly: HiFi reads of Pacbio Sequel II system and ultralong reads of Oxford Nanopore

--Manuscript Draft--

**Manuscript Number:**	GIGA-D-20-00061R2
**Full Title:**	Comparison of the two up-to-date sequencing technologies for genome assembly: HiFi reads of Pacbio Sequel II system and ultralong reads of Oxford Nanopore
**Article Type:**	Technical Note
**Funding Information:**	
**Abstract:**	The availability of reference genomes has revolutionized the study of biology. Multiple competing technologies have been developed to improve the quality and robustness of genome assemblies during the last decade. The two widely-used long-read sequencing providers – Pacbio (PB) and Oxford Nanopore Technologies (ONT) – have recently updated their platforms: PB enables high throughput HiFi reads with base-level resolution with >99% and ONT generated reads as long as 2 Mb. We applied the two up-to-date platforms to one single rice individual and then compared the two assemblies to investigate the advantages and limitations of each. The results showed that ONT ultralong reads delivered higher contiguity producing a total of 18 contigs of which ten were assembled into a single chromosome compared to that of 394 contigs and three chromosome-level contigs for the PB assembly. The ONT ultralong reads also prevented assembly errors caused by long repetitive regions for which we observed a total of 44 genes of false redundancies and ten genes of false losses in the PB assembly leading to over/under-estimation of the gene families in those long repetitive regions. We also noted that the PB HiFi reads generated assemblies with considerably fewer errors at the level of single nucleotide and small InDels than that of the ONT assembly which generated an average 1.06 errors per Kb and finally engendered 1,475 incorrect gene annotations via altered or truncated protein predictions.
**Corresponding Author:**	Shanlin Liu
**Corresponding Author Secondary Information:**	
**Corresponding Author's Institution:**	
**Corresponding Author's Secondary Institution:**	
**First Author:**	DanDan Lang
**First Author Secondary Information:**	
**Order of Authors:**	DanDan Lang
	Shilai Zhang
	Pingping Ren
	Fan Liang
	Zongyi Sun
	Guanliang Meng
	Yuntao Tan
	Xiaokang Li
	Qihua Lai
	Lingling Han
	Depeng Wang

Powered by Editorial Manager® and ProduXion Manager® from Aries Systems Corporation

	Fengyi Hu
	Wen Wang
	Shanlin Liu
**Order of Authors Secondary Information:**	
**Response to Reviewers:**	Dear Editor,<br><br>Thank you for handling the review of our manuscript. We appreciate your rapid feedback and the constructive reviews from the editorial board members and the reviewers. We have comprehensively addressed this feedback in our response below. We hope that this version of our manuscript is now suitable for publication in GigaScience.<br><br>Sincerely<br><br>Shanlin Liu<br><br><br>Comments from the Editorial Board Members:<br><br>1) Reviewer 2 in particular agrees with reviewer 3 that a direct comparison of similar methods would have been preferred - please discuss this in the manuscript.<br><br>>>> Thank you for your suggestion. We agreed that readers could be interested in not only the N50 value of the assemblies generated by different software, but also the assembly accuracy. In the revised version, we added the assembly accuracy estimations for all the assemblies. As a result, it now includes the comparisons between the assemblies generated using same software and analysis pipeline (Lines 169-174 and Figure 3).<br><br>2) Also the use of a new assembly method is problematic, as it is not well known in the field. I understand that validating this new method is outside the scope of your paper, but I recommend you mention this also as a limitation in the manuscript.<br><br>>>> We added this limitation at lines 183-189. It reads “However, the current study has several limitations, including, among others, (1) NextDenovo which generated the most contiguous assembly for the ONT is a newly developed assembler that has not been validated its performance on other species; (2) the rice which has a relatively small and simple genome cannot characterize the full spectrum of the strength and weakness of the two sequencing technologies. Genome studies, especially for those large and complex genomes, will shed more light on this matter.”. Furthermore, we noted that the developer of NextDenovo have updated their Github page which now includes its performance benchmarking to several widely-used assemblers, such as Canu, Flye, et al., using human genome.<br><br>3) I recommend that you also briefly discuss the concern that, being a case study in rice, the results may not be readily applicable to other species, as each species has its own challenges.<br><br>>>> Agree. Please find the above response #2.<br><br>Please also address the other latest comments of reviewers 1 and 2 in a second revised manuscript.<br>(I note that reviewer 2 could not access the FTP for supporting data- not quite sure where the problem is, as it seems to be working at my end ... our data curators can help the reviewer, if needed).<br><br>>>> It will be great that you can help the reviewer #2 to get the data on the FTP in the case that he/she fails to access the NCBI data as well.<br><br><br>Comments from the Reviewers:

Reviewer #1: Thanks for address most of the points I raised. The revised manuscript is a good improvment. Thanks. One minor thing, I am not sure the term "one suite of a diploid genome" is the right way to describe one single haplotype of the homologous chromosomes, please consider to the revise that for the manuscript.

>>> Thank you for your suggestion. We changed it to “one set of the paired chromosomes” at line 248 according to your advice.

Reviewer #2: The authors have addressed the concerns I raised in my first review. However, I agree with reviewer#3 on numerous points and the authors responses do raise more questions than they answer.

The authors state:
"It is weird that the reviewer argued about the reliability of its assembly results because it generated a much better results compared to the other software. It is worth noting that its readme text on github states that it performs well especially for ONT ultra-long reads."

Reviewer#3 is saying that there is no information on this assembler and relying on N50 is not a good gauge of whether the assembler is doing a good job. Also, it doesn't really matter what the readme states on github. Until a technology is proven to work, and in this case work well with ONT data, it is impossible to judge without evidence.

These comments also exposed an aspect of the paper which could be improved. The authors are arguing they are trying to make a dataset that will inform researchers how to leverage sequencing platforms for a specific goal. However, the analysis is not parallel in the sense that the authors don't compare similar assembly and polishing methods. It is great that the authors added the results from other assemblers. What would be even better is if the analysis was augmented to compare each of those assemblies. At the very least the main comparison should use the same assembly method.

>>> Thank you for your reminding. We realized that the good performance of this new assembly method (NextDenovo) for rice cannot prove that it can give equivalent performances to other species as well, and this might be a big flaw of the current study. Therefore, we firstly included some additional discussions to expose the limitations of the current work, and also included the comparisons that used the same assembly method. Please find our response #1 and #2 to the editorial board members.

Reveiwer#3 also made several other good points that the authors should take more care in addressing.

The methylation addition was a highlight. Since the technologies are moving so fast, and this manuscript is really about technology, have the authors tried the new methylation aware base-calling for ONT? Since so many of the base calling errors in ONT are due to modified bases at this point, it seems very important for the authors to present the most up to date analysis.

>>> We used the latest official release software GUPPY for basecalling, in which we failed to find any parameters specific for methylation. However, as far as we know, the performance of any particular ONT basecaller is influenced by the data used to train its model. Therefore, basecalling for native DNA (not PCR products) can perform much better in the case that their modifications and sequence motifs are represented in its training set compared to that not [1]. Inclusion of a species-specific training set for rice is feasible and will benefit the assembly accuracy for the ONT assemblies, which, however, violating our initial purpose of this study. Because most species cannot achieve such a training set as they do not have genome sequences that are publicly available, and will make the current work an unfair comparison. We added this alternative solution at lines 151-153. It reads “Providing a training set that includes information of modifications and sequence motifs of rice could at some extent alleviate the error rate of the ONT assembly.”.

Thank you for including the FTP. After several tries on different days, I could not download the full assemblies to validate the claims.

Powered by Editorial Manager® and ProduXion Manager® from Aries Systems Corporation

	>>> Please find our response #4 to the editorial board members.<br><br>Minor edits<br>These are not assembly errors, they are SNPs/INDELs resulting from mis-called bases.<br>138 "identify assembly errors under the assumption that HiFi reads provide high-level…"<br><br>>>> Corrected.<br><br>"suggesting" would be more accurate then revealing since<br>134 "revealing a limited performance of short-reads-based genome polishing methods for"<br><br>>>> Corrected.<br><br>Reword "by PB, or regions with high methylation level where ONT errors enriched", PB is not an assembler<br>"discrepancies on Chr. 6 showed that they were repeated regions incorrectly assembled by PB, or regions with high methylation level where ONT errors enriched (Supplementary Methods and Figure S11)."<br><br>>>> We corrected it to "using PB reads".<br><br>Reference<br>1. Wick RR, Judd LM, Holt KE. Performance of neural network basecalling tools for Oxford Nanopore sequencing. Genome Biol. 2019;20:129.
**Additional Information:**	
**Question**	**Response**
Are you submitting this manuscript to a special series or article collection?	No
**Experimental design and statistics**<br><br>Full details of the experimental design and statistical methods used should be given in the Methods section, as detailed in our Minimum Standards Reporting Checklist. Information essential to interpreting the data presented should be made available in the figure legends.<br><br>Have you included all the information requested in your manuscript?	Yes
**Resources**<br><br>A description of all resources used, including antibodies, cell lines, animals and software tools, with enough information to allow them to be uniquely identified, should be included in the	Yes

Methods section. Authors are strongly encouraged to cite Research Resource Identifiers (RRIDs) for antibodies, model organisms and tools, where possible.<br><br>Have you included the information requested as detailed in our Minimum Standards Reporting Checklist?	
**Availability of data and materials**<br><br>All datasets and code on which the conclusions of the paper rely must be either included in your submission or deposited in publicly available repositories (where available and ethically appropriate), referencing such data using a unique identifier in the references and in the “Availability of Data and Materials” section of your manuscript.<br><br>Have you have met the above requirement as detailed in our Minimum Standards Reporting Checklist?	Yes

Manuscript

Click here to access/download;Manuscript;manuscript-20200818_forupload.docx

Comparison of the two up-to-date sequencing technologies for genome assembly: HiFi reads of Pacbio Sequel II system and ultralong reads of Oxford Nanopore

Dandan Lang[1#], Shilai Zhang[2#], Pingping Ren[1], Fan Liang[1], Zongyi Sun[1], Guanliang Meng[1], Yuntao Tan[1], Xiaokang Li, Qihua Lai, Lingling Han[1], Depeng Wang[1], Fengyi Hu[2], Wen Wang[3,4*], Shanlin Liu[1,5*]

1. GrandOmics Biosciences, Beijing, 102200, China
2. State Key laboratory for Conservation and Utilization of Bio-Resources in Yunnan, Research Center for Perennial Rice Engineering and Technology of Yunnan, School of Agriculture, Yunnan University, Kunming, Yunnan, 650091, China
3. State Key Laboratory of Genetic Resources and Evolution, Kunming Institute of Zoology, Chinese Academy of Sciences, 650223 Kunming, Yunnan, China.
4. Center for Ecological and Environmental Sciences, Key Laboratory for Space Bioscience & Biotechnology, Northwestern Polytechnical University, 710072 Xi'an, China.
5. Department of Entomology, College of Plant Protection, China Agricultural University, 100193 Beijing, China

#Contribute equally

*Correspondence to Shanlin Liu: liushanlin@grandomics.com & Wen Wang: wwang@mail.kiz.ac.cn

Abstract

The availability of reference genomes has revolutionized the study of biology. Multiple competing technologies have been developed to improve the quality and robustness of genome assemblies during the last decade. The two widely-used long-read sequencing providers – Pacbio (PB) and Oxford Nanopore Technologies (ONT) – have recently updated their platforms: PB enables high throughput HiFi reads with base-level resolution with > 99% and ONT generated reads as long as 2 Mb. We applied the two up-to-date platforms to one single rice individual and then compared the two assemblies to investigate the advantages and limitations of each. The results showed that ONT ultralong reads delivered higher contiguity producing a total of 18 contigs of which ten were assembled into a single chromosome compared to that of 394 contigs and three chromosome-level contigs for the PB assembly. The ONT ultralong reads also prevented assembly errors caused by long repetitive regions for which we observed a total of 44 genes of false redundancies and ten genes of false losses in the PB assembly leading to over/under-estimation of the gene families in those long repetitive regions. We also noted that the PB HiFi reads generated assemblies with considerably fewer errors at the level of single nucleotide and small InDels than that of the ONT assembly which generated an average 1.06 errors per kb and finally engendered 1,475 incorrect gene annotations via altered or truncated protein predictions.

Key words: assembly comparison, ONT ultralong, PB HiFi, CCS, single-molecular sequencer, contiguity

Findings

The availability of reference genomes has revolutionized the study of biology. The high quality human reference genome enabled the identification of disease causative alleles [1,2]; the genomes of agricultural crops have tremendously accelerated our understanding of how artificial selection shaped plant traits and how, in turn, these plant traits may influence species interactions, e.g. phytophagous insects, in agriculture [3,4]. During the last decade, multiple competing technologies have been developed to improve the quality and robustness of genome assemblies [5–8], enabling genome reference collecting of the tree of life [9–11]. To date, a large number of genomes have been assembled by Third Generation Sequencing (TGS) technologies which can produce individual reads in the range of 10~100 kb or even longer [12–15]. Although the long-read methods still have a high error rate, they have been improving owing to the advances in sequencing chemistry and computational tools. For example, the Pacbio (PB) Single-molecule real-time (SMRT) sequencing platform released the Sequel II system. The updated SMRT cell enabled high throughput HiFi reads using the circular consensus sequencing (CCS) mode to provide base-level resolution with > 99% single-molecule read accuracy [16]; while the Oxford Nanopore Technologies (ONT) launched its PromethION platform which can yield > 7 Tb per run and its ultralong sequencing application facilitates the achievement of complete genome - Telomere to Telomere (T2T) - by resolving long and complex repetitive regions for various species including *Homo sapiens* [17]. The two cutting edge sequencing technologies have enabled the sequencing of many species; however, almost all chose one single sequencing system, either the PB or the ONT platform, to obtain their reference genomes [15,18,19]. Here we present one rice individual (*Oryza sativa* ssp. *indica*, 2n = 2x = 24, variety 9311) [20,21] that was sequenced and assembled independently using the two up-to-date systems, and we compare the two assemblies to investigate the advantages and limitations of each.

Findings

Following DNA extraction from the rice sample, we sequenced the two extracts using ONT PromethION and PB Sequel II platforms, respectively. The PromethION generated a total of 92 Gb data (230X) with an N50 of 41,473 bp, and the Sequel II produced a total of 253 Gb data (632X) with each molecular fragment being sequenced 14.72 times on average and produced ca. 20 Gb HiFi reads (50X) with an average length of 13,363 bp. We applied multiple software, including Canu1.9 [22], NextDenovo2.0-beta.1 (https://github.com/Nextomics/NextDenovo), WTDBG2.5 [23], Flye2.7.1 [24], SHASTA-0.4.0 [25] and NECAT (https://github.com/xiaochuanle/NECAT) to assemble the rice genome for both the ONT and PB dataset (Table S1), and then selected the optimal assembly for each sequencing platform based on contig N50 (Table S2). The ONT assembly showed higher contiguity with a contig number of 18 and an N50 value of ca. 32 Mb in comparison to a contig number of 394 and N50 of 17 Mb for the PB assembly (Figure 1a). Ten and three out of the total 12 autosomes were assembled into a single contig in the ONT and PB assembly, respectively. We identified telomeres and centromeres for both assemblies and found that seven of them reached a T2T level assembly with no gaps and no Ns in between (Table S3). A genome completeness assessment using BUSCOv3.1.0 [26] finds both assemblies performed well with the ONT having a tiny improvement (98.62% vs 98.33%, Table S4). We mapped both assemblies to a high-quality rice (R498) genome reference [20] using Minimap2 [27]. Both assemblies showed good collinearity (Figure S1) and the PB assembly contained more gaps compared to that of ONT (Figure 1a).

We then randomly took one chromosome (Chr. 6) where ONT's one single contig (32,367,127 bp) corresponded to nine contigs (32,476,323 bp) of the PB assembly to investigate and visualize the incongruencies between them. For the nine contigs of PB assembled for the Chr. 6, four reached a length ≥ 6 Mb and five had a length of merely 10-70 kb. We investigated the three gaps where the top four PB contigs (named as PB-L1, PB-L2, PB-L3 and PB-L4 from 5' to 3'end, respectively) failed to connect (Figure 1b). We mapped the ONT ultralong reads to those gaps and confirmed their correctness

through manual inspections by IGV plot [28](Figure S2). The gap #1 between PB-L1 and PB-L2 reached a length of 74,888 bp. One of the short PB contigs (PB-S1, length of 70,208 bp) had an overlap of ~10 kb with the 3' end of PB-L1, thus left the gap #1 a region of 15,722 bp that PB failed to cover (Figure 1c). We further examined the sequences obtained by ONT in and flanking this gap. It showed that the overlapping and the gap regions represented two elements of 15 kb and 48 kb in length that, although have only one copy on Chr. 6, can find their duplications on Chr. 5 (Figure S3). Repetitive elements with such lengths go beyond the typical length generated by PB CCS, therefore the right path can hardly be disentangled from complicated string graphs [22,29]. The gap #2 between PB-L2 and PB-L3 characterized a region spanning up to 48 kb on the ONT assembly and is flanked by two tandem repeats of 14 kb in length. It was spanned by multiple ONT long reads (Figure S2), so can be successfully connected by the ONT assembly. The last gap between PB-L3 and PB-L4 can be connected by one short PB contig (PB-S2, 25,292 bp), which had 9,469 and 2,621 bp overlaps with 3'end of PB-L3 and 5'end of PB-L4, respectively. And it showed the same case as gap #2, containing three tandem duplicates of length 23 kb that failed to be connected by PB HiFi reads. We found a total of 107 kb redundancies and 15 kb gaps on Chr. 6 owing to PB's incorrect assembly, which corresponded to an excess of 13 annotated genes (Figure 2, Table S5). The genome-wide misassembled regions accumulated to a length of ~ 668 kb (534 kb redundancies and 134 kb gaps), hosting 54 annotated genes (44 redundancies and 10 loss, Table S5). As PB assembly did not generate any single contigs that ONT broke into multiple segments, we cannot find a counter case for comparison. In addition, a down-sampling test showed that the ONT dataset, unlike the PB data, can produce genome assemblies of the same contiguity level using half or one-third of raw reads, corroborating the central role that ultralong reads played in assembling genome regions with long repeats (Figure S4 and Table S6). It is also worth noting that PB can run in long read mode [30], which, although can hardly generate reads as long as the ONT ultralong reads, can aid in connecting some of the gaps caused by long repeats. Besides, longer PB libraries with HiFi reads reaching 20 kb [31] would be conducive to assembly contiguity as well.

In addition to those gaps that PB failed to connect, we noticed that there were a bunch of small-scale mismatches (< 85 bp) between the two assemblies. Firstly, we extracted the reciprocal matches ≥ 1 M between the two assemblies for comparison using QUAST [32]. Then, we mapped the PB HiFi reads to both genome assemblies to identify SNVs/InDels under the assumption that HiFi reads provide high-level single-base accuracy. It showed that the ONT assembly, although polished using 70X Illumina's shotgun reads, still contained a large number of errors. In total, we found 210,993 single nucleotide errors and 211,517 InDels (Mean: 1.39 bp, Figure S5) accounting for an average number of 1.06 errors per kb. However, instead of scattering evenly on the assembly, those errors formed into clusters (Figure S6). A further investigation for those regions showed ~ 94% of them have a shotgun read coverage ≤ 5, which explains why the last polishing step failed to fix those errors (Figure S7a). As those regions were well covered by ONT long reads (Figure S7b), we examined the GC content and methylation profiles for them speculating that different methylation patterns in such regions may have reduced the base calling accuracies there. The results showed that those ONT error-enriched regions contained higher or lower GC content and significantly higher methylation level compared to other genome regions (Figure S8), hence providing a training set that includes information of modifications and sequence motifs of rice for the neural network basecalling tools could at some extent alleviate the error rate of the ONT assembly [33]. We also found that 7.48 % of those errors located on exons and affected ~ 2,415 exons (1,475 genes) to translate correctly to amino acid sequences on the ONT genome assembly. Most of those affected genes have multiple paralogous copies on the genome (Figure S9), rather than being single-copy orthologs utilized in the BUSCO analysis, suggesting a limited performance of short-reads-based genome polishing methods for duplicated genes on the genome. In addition, we did note that the errors of HiFi reads may be enriched in sequences with particular characteristics, rather than completely random, for example, regions like simple sequence repeats and long homopolymers (Supplementary Methods, Figure S10) which may exacerbate the above error statistics for the ONT assembly. What's more,

QUAST also reported some mismatches > 85 bp between the two assemblies. A manual examination for several randomly-selected discrepancies on Chr. 6 showed that they were repeated regions incorrectly assembled using PB reads, or regions with high methylation level where ONT errors enriched (Supplementary Methods and Figure S11).

Instead of using the assemblies generated by two different methods (Canu versus NextDenovo), a further examination for the two sequencing techniques using the same assembly methods (Supplementary Methods) achieved similar results: all assemblers produced a more contiguous genome assembly but with a loss of accuracy using the ONT ultralong reads compared to that using the PB HiFi reads (Figure 3 and Figure S12).

In conclusion, our study investigated genome assembly qualities between the two up-to-date competing long read sequencing techniques - the PB's HiFi reads and the ONT's ultralong reads. It showed both techniques had their own merits with: (1) ONT ultralong reads delivered higher contiguity and prevented false redundancies caused by long repeats, which, in our case of the rice genome, assembled 10 out of the 12 autosomes into one single contig, and (2) PB HiFi reads produced fewer errors at the level of single nucleotide and small InDels and obtained more than 1,400 genes that incorrectly annotated in the ONT assembly due to its error-prone reads. However, the current study has several limitations, including, among others, (1) NextDenovo which generated the most contiguous assembly for the ONT is a newly developed assembler that has not been validated its performance on other species; (2) the rice which has a relatively small and simple genome cannot characterize the full spectrum of the strength and weakness of the two sequencing technologies. Genome studies, especially for those large and complex genomes, will shed more light on this matter. Therefore, we suggest that further genome reference constructions should leverage both techniques to lessen the impact of assembly errors and subsequent annotation mistakes rooted in each. There is

also an urgent demand for improved assembly and error correction algorithms to fulfill this task.

Methods

Sample preparation and sequencing

The DNA used for ONT and PB sequel II platform sequencing were isolated from leaf tissues using SDS method and Q13323kit (QIAGEN), respectively (Supplementary Methods). The ONT platform generated a total of 6,100,295 pass reads with an average quality of 8.99 within 20 hours, and the PB sequel II platform generated a total of 21,986,306 subreads with each molecular fragment being sequenced 14.72 times on average within 30 hours. Then, the PB subreads converted to HiFi reads using ccs (https://github.com/PacificBiosciences/ccs) with default parameters. Additionally, we generated a total of 188,590,034 shotgun reads (~70X) using a strategy of pair-end 150 bp (PE 150) on the MGISEQ-2000 platform.

Genome assembly and polishing

After the genome assembly (Table S1), we mapped the ONT raw reads and PB HiFi reads onto their corresponding genomes using Minimap2 [27] and conducted genome polishing using RACON (Racon, RRID:SCR_017642) [34] through three iterations. Then, for the ONT assembly we applied Medaka, a tool designed for ONT error correction, to conduct genome polishing once more. After that, NextPolish1.1.0 [35] was applied to fix small-scale errors (SNVs and InDels) for the ONT assembly using shotgun reads. We did not apply the shotgun-read-based polishing step to the PB assembly, since HiFi reads of PB platform have already reached an accurate rate of 99% as high as that of the shotgun reads. Finally, ONT assembly generated by NextDenovo and PB assembly generated by Canu (Canu, RRID:SCR_015880) were selected out based on N50 value (Table S2) and used for the following comparison analyses.

Identification for Centromeres and Telomeres

We identified centromere and telomere-related sequences using the RCS2 family repeats and 5'-AAACCCT-3' repeats, respectively [20,36]. For centromeres, we first aligned the sequences of RCS2 family (AF058902.1) onto both the ONT and PB assemblies using BWA-MEM (BWA, RRID:SCR_010910) [37], and regions that contained full units of RCS2 family were identified as centromeres. Telomeres were identified by searching for 5'-AAACCCT-3' repeats on each contig using Tandem Repeats Finder with default parameters [38].

Assembly comparison

Collinearity: We aligned both assemblies to a high-quality rice genome (variety R498, Accession ID: GCA_002151415.1) using minimap2 [27] with a parameter setting of -x asm5. Then, we visualized the collinearity between the reference and query genomes using dotPlotly (https://github.com/tpoorten/dotPlotly, -t, -l, -m 30000, -q 1000000).

Gap identification: We aligned the PB assembly onto the ONT assembly using minimap2 [27] (-x asm5) and kept the primary hit for each contig. Then, we examined the alignment boundaries for each contig and identified the corresponding gap positions for each contig.

Identification of mismatches between ONT and PB assembly: we extracted the reciprocal matches $\geq$ 1 M between the two assemblies for comparison using QUAST 5.0.2 (QUAST, RRID:SCR_001228) with default parameters [32]. QUAST categorized mismatches into two different types: local mismatches > 85 bp and small-scale mismatches including SNVs and small InDels.

Identification of errors in forms of single nucleotide and small Indels: We aligned PB HiFi reads onto the ONT assembly and then identified SNPs and InDels using GATK4 (GATK, RRID:SCR_001876) [39] with filtering parameters: QD < 2.0 || MQ < 40.0 || FS > 60.0 || SOR > 3.0 || MQRankSum < -12.5 || ReadPosRankSum < -8.0 for SNPs, and QD < 2.0 || FS > 200.0 || SOR > 10.0 || MQRankSum < -12.5 || ReadPosRankSum < -8.0 for InDels. Given that both the PB and ONT assembly contain one set of the paired chromosomes and the discrepancies between them can present the heterozygous sites in the genome, we removed those that were identified to be

heterozygous, and regarded those homozygous derived alleles (1/1) as ONT errors.

Gene loss and redundancies: In the case that multiple PB assembly contigs mapped onto the same regions of the ONT assembly, we defined the relatively shorter ones as redundancies conditional on the following two criteria: (1) have a similarity score ≥ 97% between each other; (2) have a total depth < 60 and both have depths < 40 (Figure 2a). In addition, the gaps (showed in Figure 1) failed to be covered or covered twice by the PB contigs were defined as losses and redundancies, respectively (Figure 2b). Finally, those regions that contained genes contributed to the final gene loss and redundancy statistics.

Incorrect translation caused by ONT errors: Firstly, we searched for ONT errors that located on exons based on gene annotations of both the ONT and PB assembly. For the exon inconsistencies between the two assemblies (present/absent and mismatches), we aligned amino acid sequences of the PB assembly onto corresponding ONT regions using exonerate [40] (--model protein2genome --refine full -n 1) to investigate how the ONT errors affected gene translation.

DNA methylation

We calculated the genome-wide methylation level for the ONT assembly using Nanopolish v0.11.1 (Nanopolish, RRID:SCR_016157) with called_sites ≥ 10. The methylation profiles and GC content were recorded throughout the genome with a window size of 1,000 bp and a step length of 500 bp. Windows that contains ≥ 5 ONT errors were defined as ONT error-enriched regions and were utilized to compare for the methylation and GC content with other genomic regions.

Availability of data and materials

The raw reads, the genome assemblies of PB (assembled using Canu1.9) and ONT (assembled using NextDenvo) are deposited on NCBI under the project ID PRJNA600693, PRJNA644721 and PRJNA644720, respectively.

Supporting data, including annotation files, assemblies and BUSCO results, are also available via the *GigaScience* database, GigaDB [41]

Competing interests

The authors declare that they have no competing interests.

References

1. Weischenfeldt J, Symmons O, Spitz F, Korbel JO. Phenotypic impact of genomic structural variation: insights from and for human disease. Nat Rev Genet. 2013;14:125–38.

2. Fujimoto A, Furuta M, Totoki Y, Tsunoda T, Kato M, Shiraishi Y, et al. Whole-genome mutational landscape and characterization of noncoding and structural mutations in liver cancer. Nat Genet. 2016;48:500.

3. Saxena RK, Edwards D, Varshney RK. Structural variations in plant genomes. Brief Funct Genomics. 2014;13:296–307.

4. Chen YH, Gols R, Benrey B. Crop domestication and its impact on naturally selected trophic interactions. Annu Rev Entomol. 2015;60:35–58.

5. Wheeler DA, Srinivasan M, Egholm M, Shen Y, Chen L, McGuire A, et al. The complete genome of an individual by massively parallel DNA sequencing. Nature. 2008;452:872–6.

6. Bentley DR, Balasubramanian S, Swerdlow HP, Smith GP, Milton J, Brown CG, et al. Accurate whole human genome sequencing using reversible terminator chemistry. Nature. 2008;456:53–9.

7. Pushkarev D, Neff NF, Quake SR. Single-molecule sequencing of an individual human genome. Nat Biotechnol. 2009;27:847.

8. Rothberg JM, Hinz W, Rearick TM, Schultz J, Mileski W, Davey M, et al. An integrated semiconductor device enabling non-optical genome sequencing. Nature. 2011;475:348–52.

9. Seberg O, Droege G, Barker K, Coddington JA, Funk V, Gostel M, et al. Global Genome Biodiversity Network: saving a blueprint of the Tree of Life–a botanical perspective. Ann Bot. 2016;118:393–9.

10. Mukherjee S, Seshadri R, Varghese NJ, Eloe-Fadrosh EA, Meier-Kolthoff JP, Göker M, et al. 1,003 reference genomes of bacterial and archaeal isolates expand

coverage of the tree of life. Nat Biotechnol. 2017;35:676.
11. Lewin HA, Robinson GE, Kress WJ, Baker WJ, Coddington J, Crandall KA, et al. Earth BioGenome Project: sequencing life for the future of life. Proc Natl Acad Sci. 2018;115:4325–33.
12. Chaisson MJP, Huddleston J, Dennis MY, Sudmant PH, Malig M, Hormozdiari F, et al. Resolving the complexity of the human genome using single-molecule sequencing. Nature. 2015;517:608–11.
13. VanBuren R, Bryant D, Edger PP, Tang H, Burgess D, Challabathula D, et al. Single-molecule sequencing of the desiccation-tolerant grass *Oropetium thomaeum*. Nature. 2015;527:508–11.
14. Gordon D, Huddleston J, Chaisson MJP, Hill CM, Kronenberg ZN, Munson KM, et al. Long-read sequence assembly of the gorilla genome. Science. 2016;352:aae0344.
15. Jiao Y, Peluso P, Shi J, Liang T, Stitzer MC, Wang B, et al. Improved maize reference genome with single-molecule technologies. Nature. 2017;546:524–7.
16. Wenger AM, Peluso P, Rowell WJ, Chang P-C, Hall RJ, Concepcion GT, et al. Accurate circular consensus long-read sequencing improves variant detection and assembly of a human genome. Nat Biotechnol. 2019;37:1155–62.
17. Miga KH, Koren S, Rhie A, Vollger MR, Gershman A, Bzikadze A, et al. Telomere-to-telomere assembly of a complete human X chromosome. bioRxiv. 2019;735928.
18. Loman NJ, Quick J, Simpson JT. A complete bacterial genome assembled *de novo* using only nanopore sequencing data. Nat Methods. 2015;12:733–5.
19. Jain M, Koren S, Miga KH, Quick J, Rand AC, Sasani TA, et al. Nanopore sequencing and assembly of a human genome with ultra-long reads. Nat Biotechnol. 2018;36:338.
20. Du H, Yu Y, Ma Y, Gao Q, Cao Y, Chen Z, et al. Sequencing and *de novo* assembly of a near complete *indica* rice genome. Nat Commun. 2017;8.
21. Yu J, Wang J, Lin W, Li S, Li H, Zhou J, et al. The genomes of *Oryza sativa*: a history of duplications. PLoS Biol. 2005;3.
22. Koren S, Walenz BP, Berlin K, Miller JR, Bergman NH, Phillippy AM. Canu: scalable and accurate long-read assembly via adaptive k-mer weighting and repeat separation. Genome Res. 2017;27:722–36.
23. Ruan J, Li H. Fast and accurate long-read assembly with wtdbg2. Nat Methods. 2020;17:155–8.
24. Kolmogorov M, Yuan J, Lin Y, Pevzner PA. Assembly of long, error-prone reads using repeat graphs. Nat Biotechnol. 2019;37:540–6.
25. Shafin K, Pesout T, Lorig-Roach R, Haukness M, Olsen HE, Bosworth C, et al. Nanopore sequencing and the Shasta toolkit enable efficient *de novo* assembly of eleven human genomes. Nat Biotechnol. 2020;1–10.
26. Seppey M, Manni M, Zdobnov EM. BUSCO: assessing genome assembly and annotation completeness. Gene Predict. 2019;227–45.
27. Li H. Minimap2: pairwise alignment for nucleotide sequences. Bioinformatics. 2018;34:3094–100.
28. Robinson JT, Thorvaldsdóttir H, Winckler W, Guttman M, Lander ES, Getz G, et al. Integrative genomics viewer. Nat Biotechnol. 2011;29:24–6.

29. Myers EW. The fragment assembly string graph. Bioinformatics. 2005;21:79–85.
30. Rhoads A, Au KF. PacBio sequencing and its applications. GPB. 2015;13:278–89.
31. Nurk S, Walenz BP, Rhie A, Vollger MR, Logsdon GA, Grothe R, et al. HiCanu: accurate assembly of segmental duplications, satellites, and allelic variants from high-fidelity long reads. bioRxiv. 2020.
32. Mikheenko A, Prjibelski A, Saveliev V, Antipov D, Gurevich A. Versatile genome assembly evaluation with QUAST-LG. Bioinformatics. 2018;34:i142–50.
33. Wick RR, Judd LM, Holt KE. Performance of neural network basecalling tools for Oxford Nanopore sequencing. Genome Biol. 2019;20:129.
34. Vaser R, Sović I, Nagarajan N, Šikić M. Fast and accurate *de novo* genome assembly from long uncorrected reads. Genome Res. 2017;27:737–46.
35. Hu J, Fan J, Sun Z, Liu S. NextPolish: a fast and efficient genome polishing tool for long read assembly. Bioinformatics. 2019.
36. Dong F, Miller JT, Jackson SA, Wang G-L, Ronald PC, Jiang J. Rice (*Oryza sativa*) centromeric regions consist of complex DNA. Proc Natl Acad Sci. 1998;95:8135–40.
37. Li H, Durbin R. Fast and accurate short read alignment with Burrows–Wheeler transform. Bioinformatics. 2009;25:1754–60.
38. Benson G. Tandem repeats finder: a program to analyze DNA sequences. Nucleic Acids Res. 1999;27:573–80.
39. McKenna A, Hanna M, Banks E, Sivachenko A, Cibulskis K, Kernytsky A, et al. The Genome Analysis Toolkit: a MapReduce framework for analyzing next-generation DNA sequencing data. Genome Res. 2010;20:1297–303.
40. Slater GSC, Birney E. Automated generation of heuristics for biological sequence comparison. BMC Bioinformatics. 2005;6:31.
41 Lang D, Zhang S, Ren P, Liang F, Sun Z, Meng G, et al. Supporting data for "Comparison of the two up-to-date sequencing technologies for genome assembly: HiFi reads of Pacbio Sequel II system and ultralong reads of Oxford Nanopore" GigaScience Database 2020. http://dx.doi.org/10.5524/100805

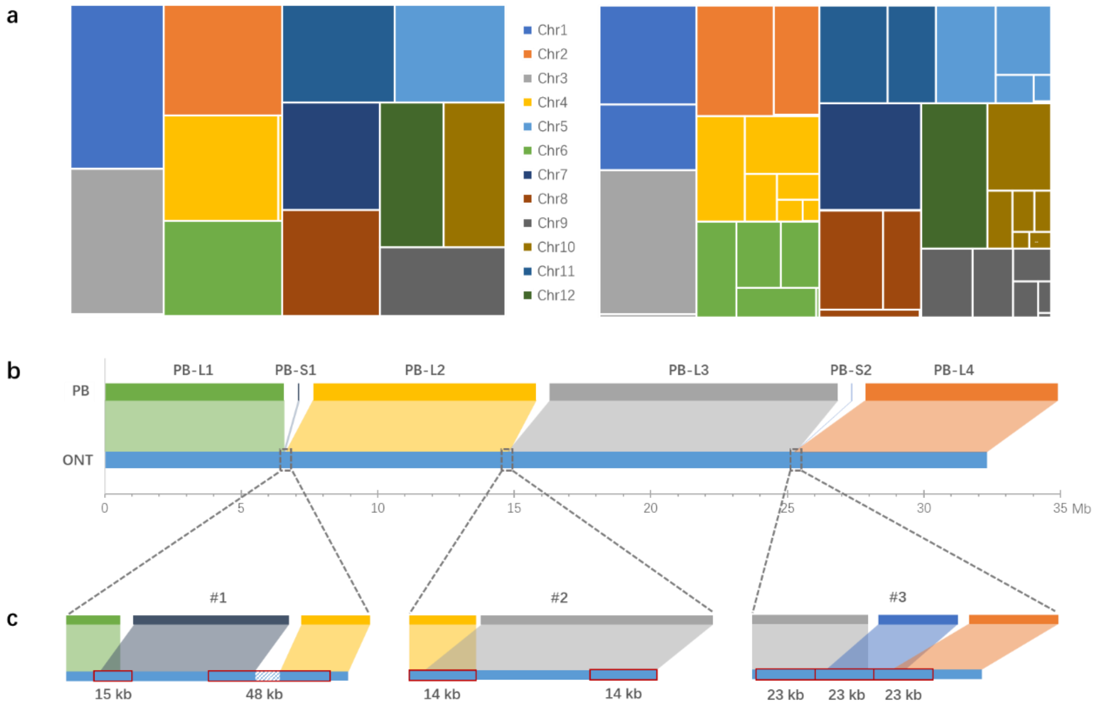

Figure 1. Contiguity of the ONT and PB assemblies. (a) Treemaps for contig length difference between the ONT (left) and PB (right) assembly; (b) The six PB contigs mapped to one ONT contig corresponding to Chr. 6; (c) Details of the three PB gaps. Red rectangles noted the repeat elements.

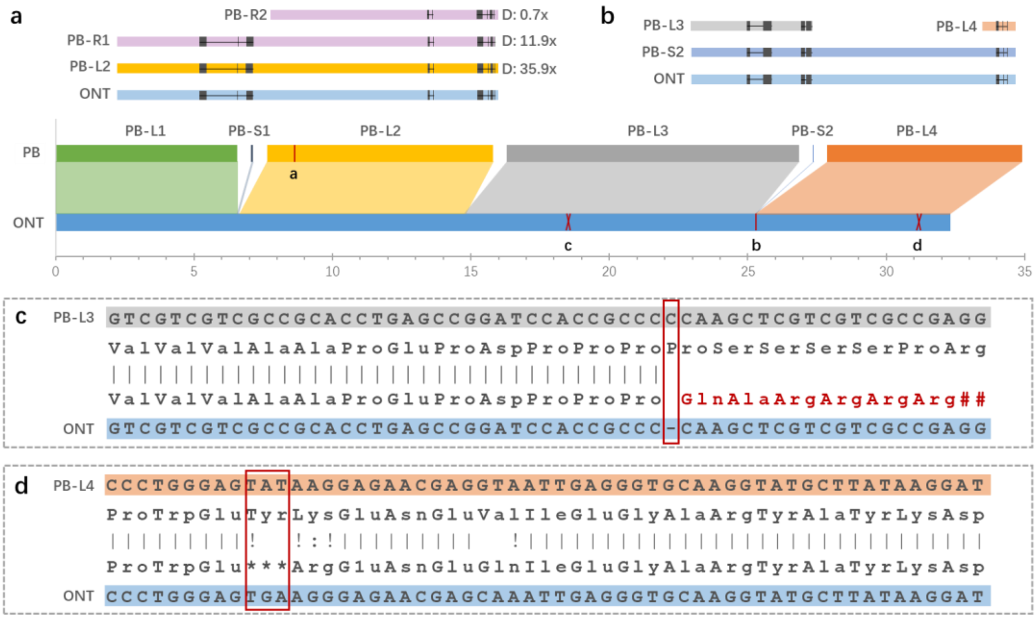

Figure 2. Assembly errors in which genes can be annotated. (a) An example shows gene gains that caused by assembly redundancies, of which the PB-R1 and PB-R2 had a similarity level of 99.67% and 99.51%, respectively, compared to the corresponding region on PB-L2, and "D" abbreviates from depth; (b) The gene redundancies caused by gaps that failed to be correctly connected by the PB assembly; (c) An example shows a 1-base deletion led to frameshift mistake for protein translation; (d) An example shows single base error led to stop codon gain and truncated protein translation.

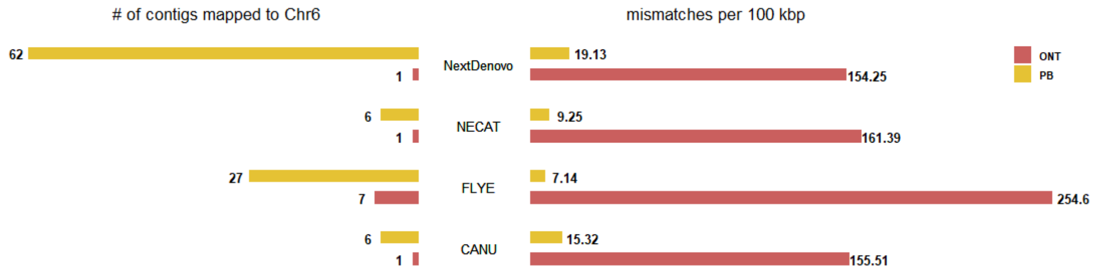

Figure 3. Assembly comparisons using the same methods. Left: number of contigs that were mapped onto Chr. 6; Right: number of mismatches (including SNVs and InDels) per 100 kb.

Figure 1

Click here to access/download
Supplementary Material
Figure 1.svg

Figure 2

Click here to access/download
Supplementary Material
Figure 2.svg

Figure 3

Click here to access/download
Supplementary Material
Figure 3.svg

FigureS1

Click here to access/download
Supplementary Material
Figure S1.pdf

Figure S2

Click here to access/download
Supplementary Material
Figure S2.svg

FigureS3

Click here to access/download

Supplementary Material

Figure S3.pdf

Figure S4

Click here to access/download
Supplementary Material
Figure S4.svg

FigureS4

Click here to access/download
Supplementary Material
Figure S5.pdf

Figure S6

Click here to access/download
Supplementary Material
Figure S6.svg

FigureS7

Click here to access/download
Supplementary Material
Figure S7.pdf

Figure S8

Click here to access/download
Supplementary Material
Figure S8.svg

FigureS9

Click here to access/download
Supplementary Material
Figure S9.pdf

FigureS10

Click here to access/download
Supplementary Material
Figure S10.pdf

FigureS11

Click here to access/download
Supplementary Material
Figure S11.pdf

Click here to access/download
Supplementary Material
Supplementary information-20200818.docx

Figure S12

Click here to access/download
Supplementary Material
Figure S12.pdf

Figure S12

Click here to access/download
Supplementary Material
Figure S12.pdf
