# Supplementary material for: Comparison of the two up-to-date sequencing technologies for genome assembly: HiFi reads of Pacific Biosciences Sequel II system and ultralong reads of Oxford Nanopore
Source: Gigascience. 2020 Dec 15;9(12):giaa123. doi: 10.1093/gigascience/giaa123 (PMC7736813; doi:10.1093/gigascience/giaa123)
Supplement: giaa123_Supplemental_Files [file giaa123_supplemental_files.zip › Supplementary information-20200818.docx]

**Supplementary Methods**

**Sample preparation and sequencing**

We extracted DNA from the rice leaves using SDS method and Q13323kit (QIAGEN) for ONT and PB respectively. We applied SDS method for the ONT platform since ONT ultralong sequencing requires more integrity DNA than the general demands of the Third Generation Sequencing. We firstly grinded the leaves carefully with liquid nitrogen and then cleaned the cell nucleus with modified buffer HB [1]. After that, the remaining was washed by 1x PBS buffer (pH=7.4, Invitrogen CAT: AM9264) and cell nucleus was lysed by Buffer TLB: 10mM Tris (pH=8.0, Solarbio CAT: T1150), 25mM EDTA, 100mM NaCl (Biosharp CAT: BL542A) and 1.25% SDS (Solarbio CAT: S1015). Next, RNA and protein were removed by RNase A (QIAGEN Lot 8600000227) and protease K (QIAGEN Lot 163024976), respectively. Finally, we eluted the DNA using Buffer EB (QIAGEN CAT: 19086) after phenol/chloroform extracting. Following DNA extraction, two extracts were constructed for ONT and PB libraries and then sequenced using PromethION and PB Sequel II platforms, respectively.

**Genome annotation**

Firstly, we characterized the repeat elements for both PB and ONT genomes using RepeatMasker (http://repeatmasker.org) with a rice repeat library downloaded from the Rice Genome Annotation Project (http://rice.plantbiology.msu.edu/annotation_oryza.shtml). Then, we applied GeMoMa-1.6.1 [2] for protein-coding gene prediction using four species: *Oryza sativa* ssp. *Indica* (R498, http://www.mbkbase.org/R498/), *Oryza sativa* ssp. *Japonica* (http://rice.plantbiology.msu.edu/pub/data/Eukaryotic_Projects/o_sativa/annotation_dbs/pseudomolecules/version_7.0/all.dir/), *Zea mays* (Accession number: GCF_000005005.2) and *Brachypodium distachyon* (Accession number: GCF_000005505.3). After that, we conducted *de novo* gene prediction using Augustus-3.3.1 [3] and combined the two prediction results using EVidenceModeler-1.1.1 [4] with a weight 1 and 5 for ABINITIO_PREDICTION (AUGUSTUS) and PROTEIN (GeMoMa), respectively.

**Mismatches of length > 85 bp between the ONT and PB assemblies**

QUAST classified the disagreements of length > 85 bp as local mis-assemblies, and we discovered a total 1,035 such disagreements between the ONT and PB assemblies. Both the ONT raw reads and PB HiFi reads were independently mapped onto the two genome assemblies. Then, we randomly selected several of those disagreements and visualized their corresponding alignments using IGV [5] and evaluated their correctness via manually checking (Figure S11).

**Performance comparison using same assembly methods**

Firstly, we applied RACON [6] to correct those SNV/InDel errors on the ONT assembly (generated by NextDenovo) using the PB HiFi reads. Then, the corrected ONT assembly served as a genome reference to evaluate the performance of the two sequencing technologies – PB HiFi reads and ONT ultralong reads - via feeding QUAST 5.0.2 [7] genome sequences generated by the same assembly methods one by one (Table S2). Assemblies generated by WTDBG and SHASTA were not included in the comparison analyses due to significant deviation from the expected genome size (~ 400 Mb). To be in line with the former comparison analyses, we utilized the Chr.6 again to illustrate the differences between the two assemblies for each method. The contiguity level of each assembly was evaluated by tallying the number of mapped nonredundant contigs that do not fully overlap with each other (Figure 2), and the assembly accuracy was calculated by adding together the “mismatches per 100 kbp” and “indels per 100 kbp” reported by QUAST.

**Supplementary Tables**

**Table S1. Assembly parameters and computational resource statistics**

|  | Assembler | Parameters | CPU time (h) | Peak RAM (G) |
| --- | --- | --- | --- | --- |
| PB  HiFi reads | CANU 1.9 | -pacbio-hifi | 158 | 36 |
|  | WDTBG-2.5 | -x ccs | 60 | 43 |
|  | NextDenovo_v2.0-beta.1 | minimap2_options_cns = -x ava-pb -k17 -w17 | 1,112 | 58 |
|  | SHASTA-0.4.0 | default | 5 | 94 |
|  | FLYE-2.7.1-b1590 | --pacbio-hifi, -i 0 | 105 | 64 |
|  | NECAT_20200119 | POLISH_CONTIGS=false | 840 | 39 |
| ONT  ultralong reads | CANU 1.9 | -nanopore-raw | 9,643 | 37 |
|  | WDTBG-2.5 | -x ont | 780 | 231 |
|  | NextDenovo_v2.0-beta.1 | read_cutoff = 1k, seed_cutoff = 68k | 1,020 | 48 |
|  | SHASTA-0.4.0 | default | 128 | 379 |
|  | FLYE-2.7.1-b1590 | --nano-raw, -i 0 | 648 | 258 |
|  | NECAT_20200119 | POLISH_CONTIGS=false | 390 | 56 |

Note: CANU version 1.9 was updated specially for PB HiFi reads. CPU time was estimated by wall clock time*thread number for each assembler.

**Table S2. Assembly quality evaluation**

|  | Assembler | Assembly size | # of Contigs | N50 |
| --- | --- | --- | --- | --- |
| PB  HiFi reads | CANU 1.9 | 404,883,979 | 394 | 17,256,392 |
|  | WTDBG-2.5 | 353,080,650 | 1,812 | 462,588 |
|  | NextDenovo_v2.0-beta.1 | 396,208,190 | 733 | 1,323,549 |
|  | SHASTA-0.4.0 | 306,794 | 96 | 5,888 |
|  | FLYE-2.7.1-b1590 | 393,297,823 | 287 | 4,359,037 |
|  | NECAT_20200119 | 390,641,382 | 217 | 7,161,524 |
| ONT  ultralong reads | CANU 1.9 | 407,654,647 | 686 | 12,297,832 |
|  | WTDBG-2.5 | 459,329,605 | 7,387 | 227,607 |
|  | NextDenovo_v2.0-beta.1 | 399,283,841 | 18 | 32,367,127 |
|  | SHASTA-0.4.0 | 324,731,392 | 2,299 | 553,961 |
|  | FLYE-2.7.1-b1590 | 403,813,869 | 850 | 12,592,690 |
|  | NECAT_20200119 | 399,890,998 | 79 | 25,824,958 |

Note: CANU version 1.9 was updated specially for PB HiFi reads.

**Table S3. The centromeres and telomeres for each chromosome-level contig of ONT and PB assemblies**

| **ONT contigs** | | | | | |
| --- | --- | --- | --- | --- | --- |
| Contig ID | Centromere position | Long arm length | Short arm length | Telomere repeat number | |
| ctg000009 (Chr.1) | 26,490,151 - 27,493,409 | 26,490,150 | 17,524,811 | 2,450 | - |
| ctg000001 (Chr.2) | 24,270,728 - 24,825,807 | 24,270,727 | 14,198,567 | 2,771 | - |
| ctg000015 (Chr.5) | 12,564,079 - 12,685,655 | 18,233,652 | 12,564,078 | - | - |
| ctg000005 (Chr.6) | 15,722,024 - 15,827,067 | 16,540,060 | 15,722,023 | 2,226 | 2,242 |
| ctg000014 (Chr.7) | 12,648,717 - 13,228,755 | 17,486,620 | 12,648,716 | 1,864 | - |
| ctg000008 (Chr.8) | 12,798,891 - 13,875,118 | 16,574,980 | 12,798,890 | 1,970 | 2,177 |
| ctg000000 (Chr.9) | 2,905,622 - 3,559,788 | 21,497,123 | 2,905,621 | - | - |
| ctg000012 (Chr.10) | 16,285,025 - 16,412,330 | 16,285,024 | 9,681,892 | 1,643 | 2,071 |
| ctg000004 (Chr.11) | 19,266,069 - 20,324,194 | 19,266,068 | 12,803,958 | 1,556 | - |
| ctg000010 (Chr.12) | 11,433,684 - 11,550,742 | 15,397,670 | 11,433,683 | 1,660 | 1,683 |
| **PB contigs** | | | | | |
| Contig ID | Centromere position | Long arm length | Short arm length | Telomere repeat number | |
| tig00000013 (Chr. 3) | 21,058,574 - 21,265,342 | 21,058,573 | 18,168,380 | 3,852 | 3,192 |
| tig00000139 (Chr. 7) | 17,493,629 - 18,180,458 | 17,493,628 | 12,650,984 | 1,718 | 2,642 |
| tig00000277 (Chr. 12) | 15,413,384 - 15,530,454 | 15,413,383 | 11,436,959 | 1,670 | 1,883 |

Note: Telomere repeat number represents the number of 5‘-AAACCCT-3’ repeat with – means fail to find.

**Table S4. Results of genome completeness assessment using BUSCO**

|  | PB assembly (%) | ONT assembly (%) |
| --- | --- | --- |
| Complete BUSCOs | 98.33 | 98.62 |
| Complete and single-copy BUSCOs | 96.36 | 96.80 |
| Complete and duplicated BUSCOs | 1.96 | 1.82 |
| Fragmented BUSCOs | 0.29 | 0.15 |
| Missing BUSCOs | 1.38 | 1.24 |
| Embryophyta | 100.00 | 100.00 |

NOTE: PB assembly represents the one assembled using CANU 1.9 and ONT assembly was obtained using NextDenovo.

**Table S5. Gene loss and** **redundancies of the PB assembly**

| **Gene loss** | | | | |
| --- | --- | --- | --- | --- |
| ONT_contig_ID | ONT_gene_ID | Start | End | # of loss |
| ctg000002 | ctg000002_G00074 | 999423 | 1034123 | 1 |
| ctg000002 | ctg000002_G00077 | 1062799 | 1069109 | 1 |
| ctg000004 | ctg000004_G01301 | 19428988 | 19446007 | 1 |
| ctg000008 | ctg000008_G01382 | 19347370 | 19353870 | 1 |
| ctg000008 | ctg000008_G02418 | 29556208 | 29684974 | 1 |
| ctg000012 | ctg000012_G00322 | 2969329 | 2970124 | 1 |
| ctg000012 | ctg000012_G00567 | 5045462 | 5046104 | 1 |
| ctg000012 | ctg000012_G00568 | 5049836 | 5052763 | 1 |
| ctg000012 | ctg000012_G00569 | 5053647 | 5056363 | 1 |
| ctg000012 | ctg000012_G00570 | 5056931 | 5058839 | 1 |
| **Gene** **redundancies** | | | | |
| ONT_contig_ID | ONT_gene_ID | Start | End | # of redundancie**s** |
| ctg000000 | ctg000000_G00597 | 11278491 | 11278830 | 2 |
| ctg000000 | ctg000000_G00598 | 11279619 | 11293649 | 1 |
| ctg000000 | ctg000000_G00081 | 1367697 | 1385458 | 1 |
| ctg000000 | ctg000000_G01574 | 21546713 | 21547382 | 1 |
| ctg000002 | ctg000002_G00074 | 999423 | 1034123 | 1 |
| ctg000002 | ctg000002_G00079 | 1080239 | 1107445 | 1 |
| ctg000003 | ctg000003_G00812 | 15707253 | 15728302 | 1 |
| ctg000004 | ctg000004_G00097 | 1418133 | 1418904 | 1 |
| ctg000004 | ctg000004_G00098 | 1420869 | 1421634 | 1 |
| ctg000005 | ctg000005_G00255 | 1931660 | 1932002 | 1 |
| ctg000005 | ctg000005_G00256 | 1933158 | 1934211 | 1 |
| ctg000005 | ctg000005_G00257 | 1940317 | 1945807 | 1 |
| ctg000005 | ctg000005_G00258 | 1947015 | 1948068 | 1 |
| ctg000005 | ctg000005_G00932 | 7747015 | 7751198 | 1 |
| ctg000005 | ctg000005_G00933 | 7762171 | 7762622 | 2 |
| ctg000005 | ctg000005_G00934 | 7766283 | 7780469 | 2 |
| ctg000005 | ctg000005_G01464 | 14805155 | 14815093 | 1 |
| ctg000005 | ctg000005_G02124 | 25314455 | 25316369 | 1 |
| ctg000005 | ctg000005_G02125 | 25318653 | 25322992 | 1 |
| ctg000005 | ctg000005_G02126 | 25333867 | 25334733 | 1 |
| ctg000006 | ctg000006_G03206 | 33368857 | 33372453 | 1 |
| ctg000006 | ctg000006_G03207 | 33373708 | 33375291 | 1 |
| ctg000006 | ctg000006_G03208 | 33380579 | 33384195 | 1 |
| ctg000006 | ctg000006_G03209 | 33384930 | 33385787 | 1 |
| ctg000006 | ctg000006_G03210 | 33386180 | 33386905 | 1 |
| ctg000008 | ctg000008_G01382 | 19347370 | 19353870 | 1 |
| ctg000008 | ctg000008_G01383 | 19356150 | 19363873 | 1 |
| ctg000008 | ctg000008_G01252 | 17263321 | 17263702 | 1 |
| ctg000008 | ctg000008_G01253 | 17263970 | 17264438 | 1 |
| ctg000008 | ctg000008_G02418 | 29556208 | 29684974 | 1 |
| ctg000012 | ctg000012_G00286 | 2690576 | 2700335 | 1 |
| ctg000012 | ctg000012_G00287 | 2703559 | 2704736 | 1 |
| ctg000012 | ctg000012_G00989 | 9408612 | 9410982 | 1 |
| ctg000012 | ctg000012_G00990 | 9411510 | 9413516 | 1 |
| ctg000012 | ctg000012_G00991 | 9416266 | 9418167 | 1 |
| ctg000012 | ctg000012_G00992 | 9419388 | 9424819 | 1 |
| ctg000015 | ctg000015_G00162 | 1369836 | 1372125 | 2 |
| ctg000015 | ctg000015_G00163 | 1373588 | 1377298 | 2 |
| ctg000015 | ctg000015_G00008 | 83886 | 101996 | 1 |

Note: the start and end positions corresponded to the coordinates on the ONT assembly.

**Table S6. Read summary of the subsampling test**

|  | **PB HiFi reads (CANU1.9)** | | | **ONT ultralong reads (NextDenovo)** | | |
| --- | --- | --- | --- | --- | --- | --- |
|  | 15X | 30X | 50X | 50X | 100X | 230X |
| Total length | 5,700,002,986 | 11,400,008,130 | 19,964,422,872 | 19,000,002,064 | 38,000,045,639 | 92,929,730,682 |
| # of Reads | 411,814 | 826,740 | 1,494,013 | 1,544,281 | 3,046,495 | 6,100,295 |
| N50 | 13,942 | 13,846 | 13,586 | 38,735 | 39,127 | 41,473 |
| Average length | 13,841 | 13,789 | 13,363 | 12,304 | 12,473 | 15,233 |

**Supplementary Figures Legend**

**Figure S1. Collinearity between genome assembly of rice R498 and that of the PB (left) and ONT (right).** Note: It only shows alignments ≥ 30 kb and query sequences ≥ 1 Mb.

**Figure S2. IGV plots of the three PB gaps on Chr. 6.** Gray shadows represent gap regions of the PB assembly. Red rectangles represent the repeat elements.

**Figure S3.** **Details of PB gap #1.** The two repetitive regions matched to another PB assembly contig corresponding to Chr5 (PB_Chr5) with high identities. IDY means similarity identities between each other. The bottom panel highlights local IDY values of 100% between each other with an alignment length of 10 kb (PB-L1 versus PB-S1), 12 kb (PB-L1 versus PB_Chr5), and 13 kb (PB-S1 versus PB_Chr5).

**Figure S4. Assembly statistics for the subsampling test.** Contig N50 value (upper) and raw read coverage (under) were demonstrated for each assembly. Assemblies applied the same parameters in Table S1 for CANU and NextDenovo.

**Figure S5. The length distribution of the ONT InDel errors.** Note that InDels of length > 20 bp had a total count of 216 and did not show here.

**Figure S6. Distances between adjacent ONT errors.** Those errors tend to cluster in the same region rather than distribute randomly and evenly on the genome, since the distances should have a peak around 1,000 bp for an average error rate of 1.06 per kb in the case of random distribution. The yellow curve represents a theoretical error distribution with a mean distance of 1,000 (SD of 200).

**Figure S7. Depth of (a) shotgun reads, (b) ONT raw reads and (c) PB HiFi reads for those ONT error sites.** Note that Illumina’s shotgun read depth > 30 had a total count of 10,294 (2.44% of total) did not show here.

**Figure S8. Comparison of GC content and methylation level between the ONT error-enriched regions and other regions for the ONT assembly.**

**Figure S9. The paralogous copy numbers distribution of the genes affected by ONT errors.** Paralogs were searched using BLAST with e-value cutoff of 1e-5 for each gene.

**Figure S10. Two examples (one SNP and one InDel) that showed the mismatches between the ONT and PB assemblies which were well covered by shotgun reads and thus could be errors on HiFi reads generated during the CCS progress.**

**Figure S11. Examples of the mismatches > 85 bp and their corresponding IGV plots for the genome alignments for the PB (upper) and ONT (bottom) assemblies**. (a) A 1,432 bp InDel where reads mapped onto PB’s assembly with soft-clips; (b) A 231 bp mismatch on which ONT’s assembly displayed a cluster of small-scale errors (GC content: 75.6%, methylation level: 91.0%). (c) A 204 bp InDel (at the end of contig tig00004207) on which no PB’s HiFi reads showed in the alignments (reads mapped onto multiple locations can have a mapping score of zero, and were removed in our analysis). We also noted that this InDel was introduced during the genome polishing step by Racon which may corrupt the correctly assembled sequence within repetitive regions.

**Figure S12. Contig alignments of Chr. 6.** Red represents contigs that contain InDel mismatches of length ≥ 85 bp, while green not. The percentage values represent the coverage ratios (total length of mapped regions / the reference length).

**References**

1. Workman R, Fedak R, Kilburn D, Hao S, Liu K, Timp W. High molecular weight DNA extraction from recalcitrant plant species for third generation sequencing. Protoc Exch. 2018;1–12.

2. Keilwagen J, Hartung F, Paulini M, Twardziok SO, Grau J. Combining RNA-seq data and homology-based gene prediction for plants, animals and fungi. BMC Bioinformatics. 2018;19:189.

3. Stanke M, Diekhans M, Baertsch R, Haussler D. Using native and syntenically mapped cDNA alignments to improve *de novo* gene finding. Bioinformatics. 2008;24:637–44.

4. Haas BJ, Salzberg SL, Zhu W, Pertea M, Allen JE, Orvis J, et al. Automated eukaryotic gene structure annotation using EVidenceModeler and the Program to Assemble Spliced Alignments. Genome Biol. 2008;9:R7.

5. Robinson JT, Thorvaldsdóttir H, Winckler W, Guttman M, Lander ES, Getz G, et al. Integrative genomics viewer. Nat Biotechnol. 2011;29:24–6.

6. Vaser R, Sović I, Nagarajan N, Šikić M. Fast and accurate *de novo* genome assembly from long uncorrected reads. Genome Res. 2017;27:737–46.

7. Mikheenko A, Prjibelski A, Saveliev V, Antipov D, Gurevich A. Versatile genome assembly evaluation with QUAST-LG. Bioinformatics. 2018;34:i142–50.
