# Supplementary figures and images for: Comparison of the two up-to-date sequencing technologies for genome assembly: HiFi reads of Pacific Biosciences Sequel II system and ultralong reads of Oxford Nanopore
Source: Gigascience. 2020 Dec 15;9(12):giaa123. doi: 10.1093/gigascience/giaa123 (PMC7736813; doi:10.1093/gigascience/giaa123)

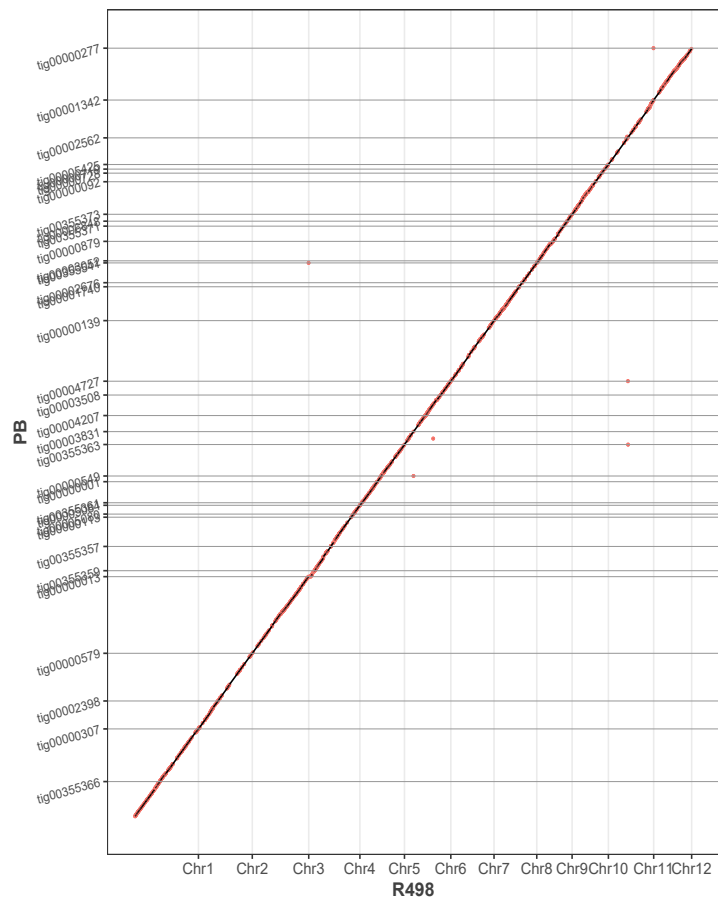

Supplement: giaa123_Supplemental_Files [file giaa123_supplemental_files.zip › Figure S1.pdf]

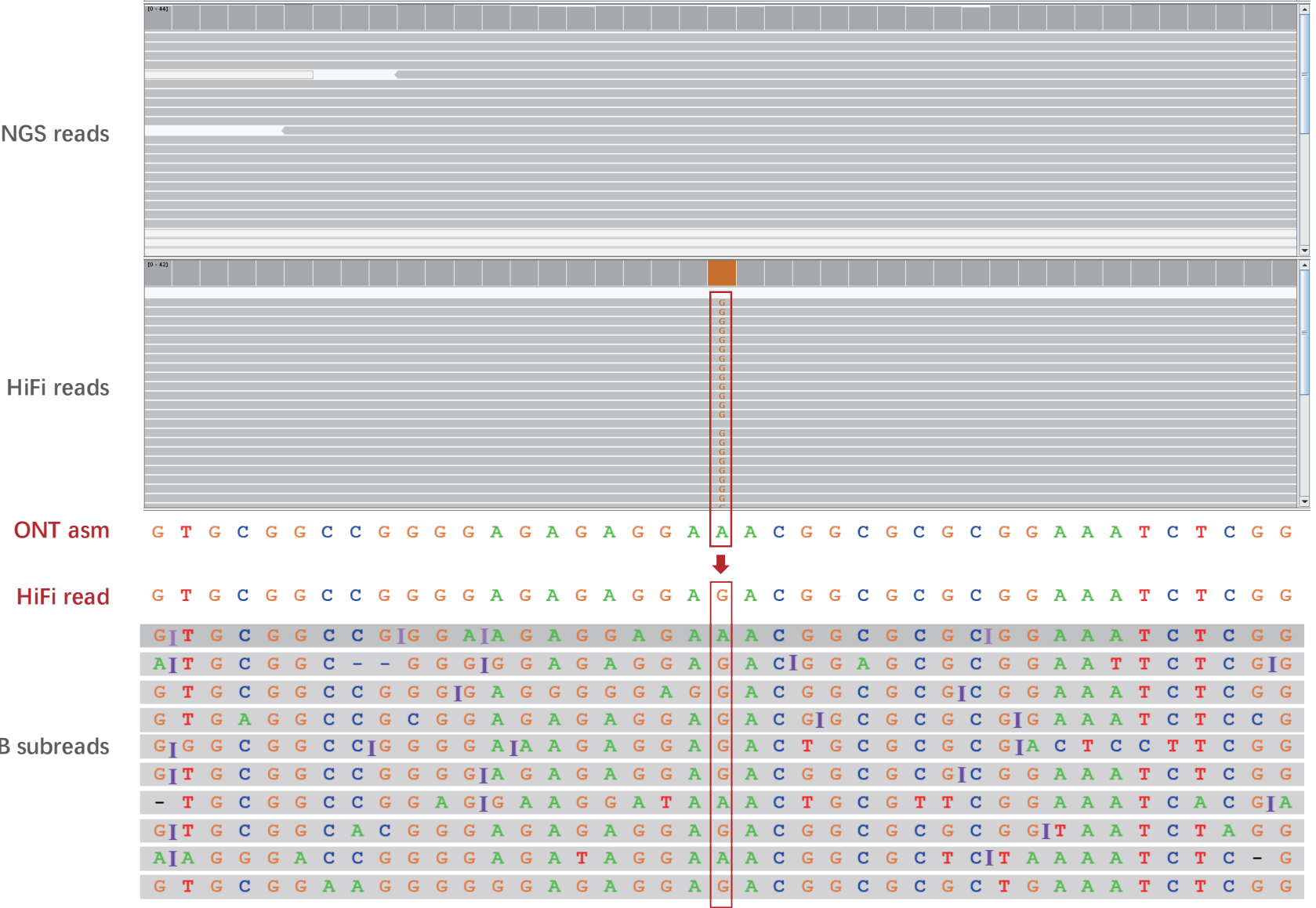

SNP

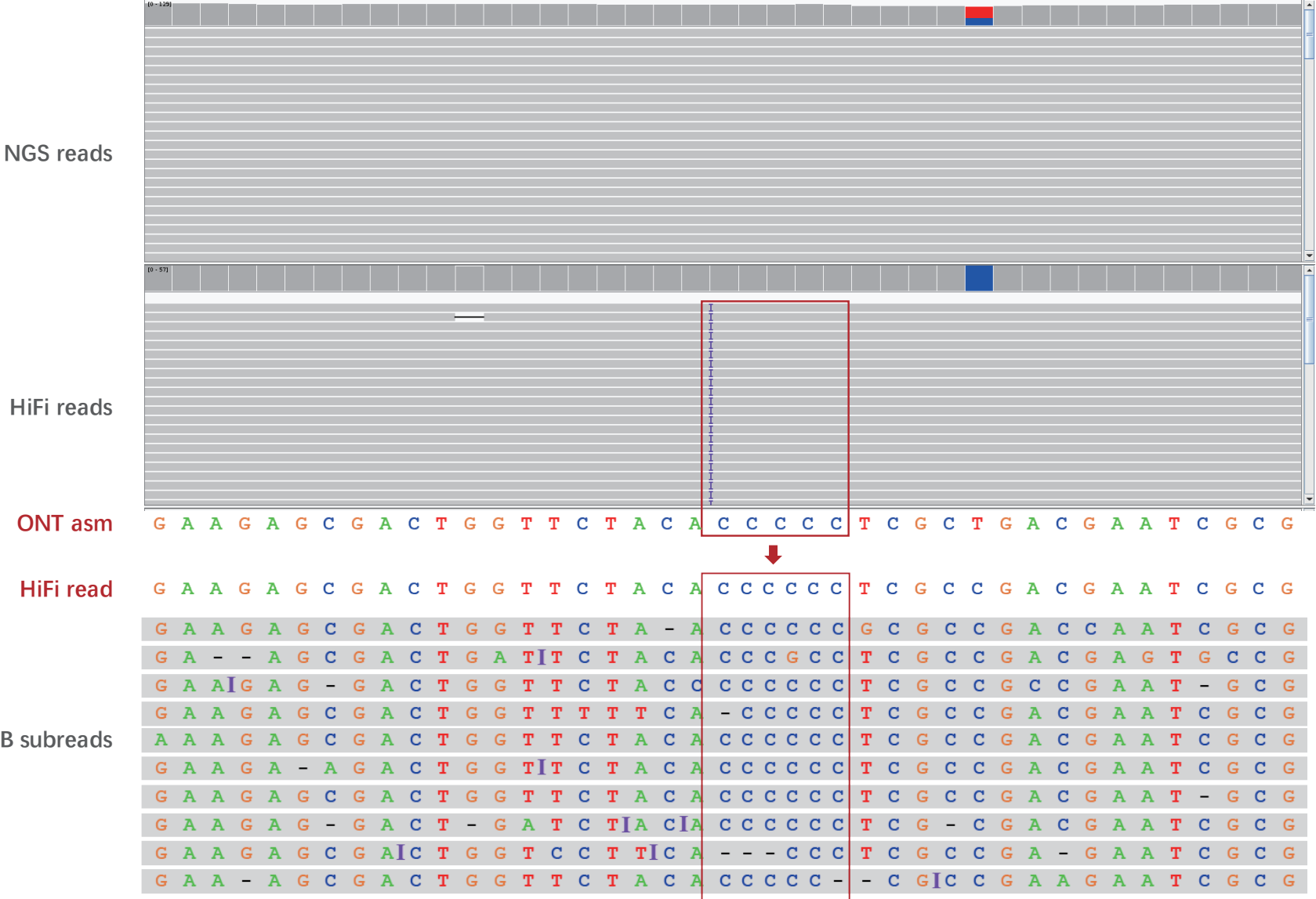

InDel

Supplement: giaa123_Supplemental_Files [file giaa123_supplemental_files.zip › Figure S10.pdf]

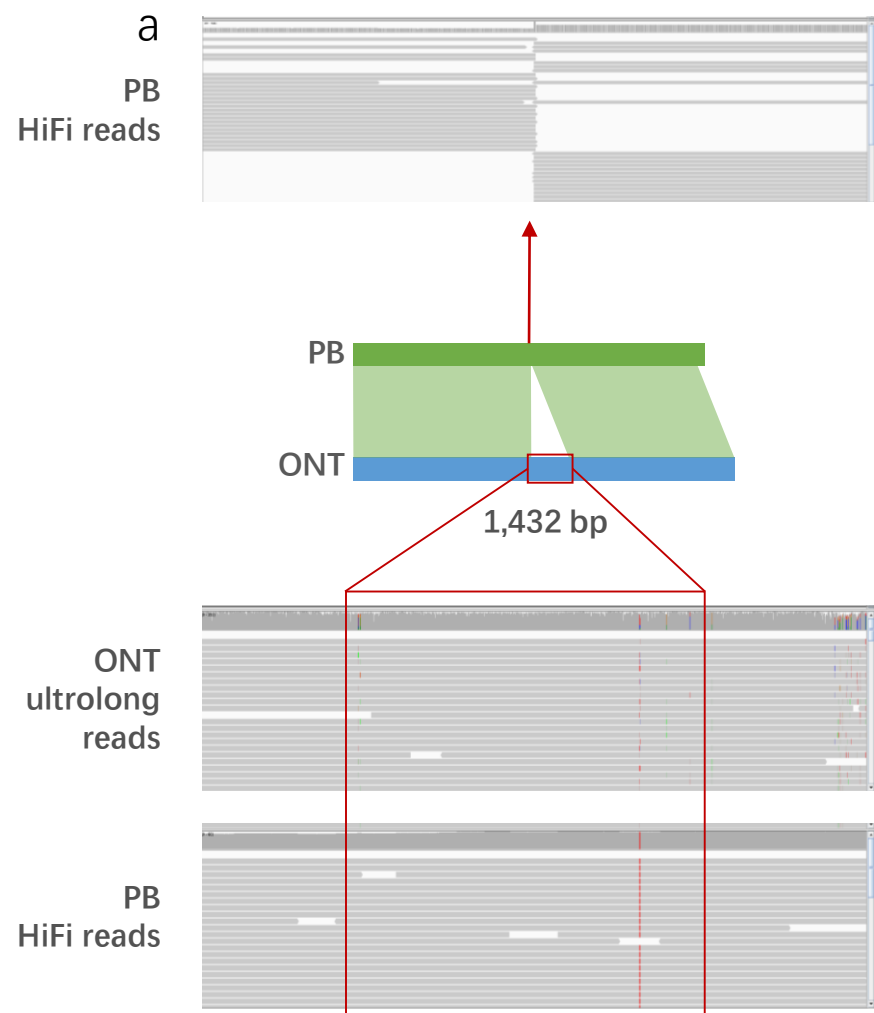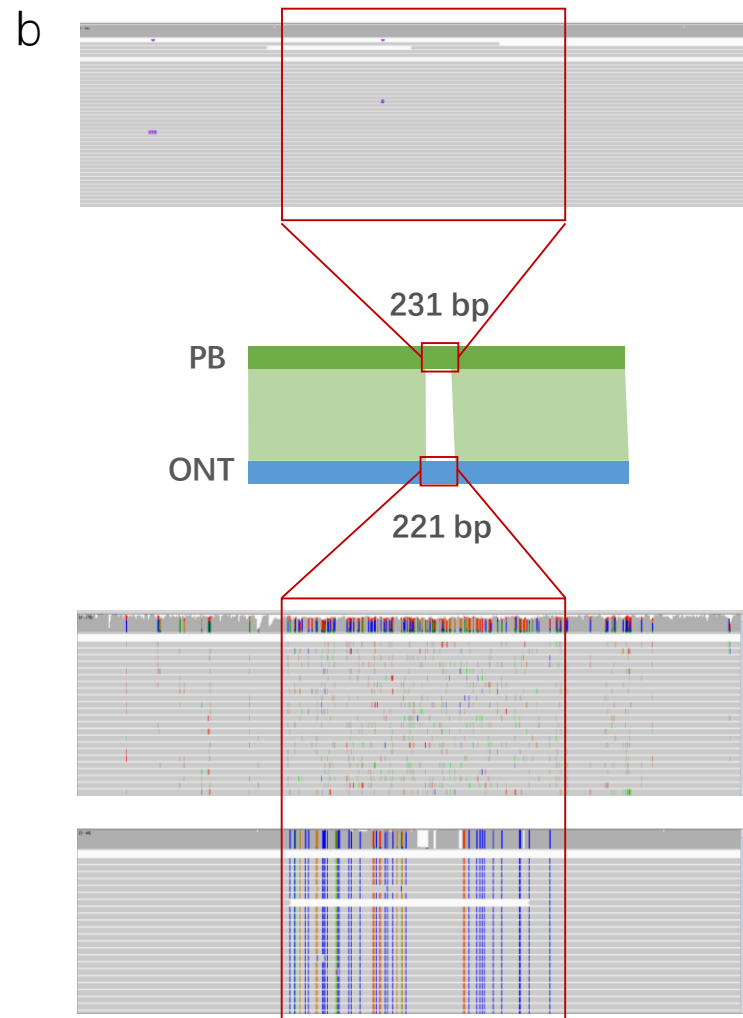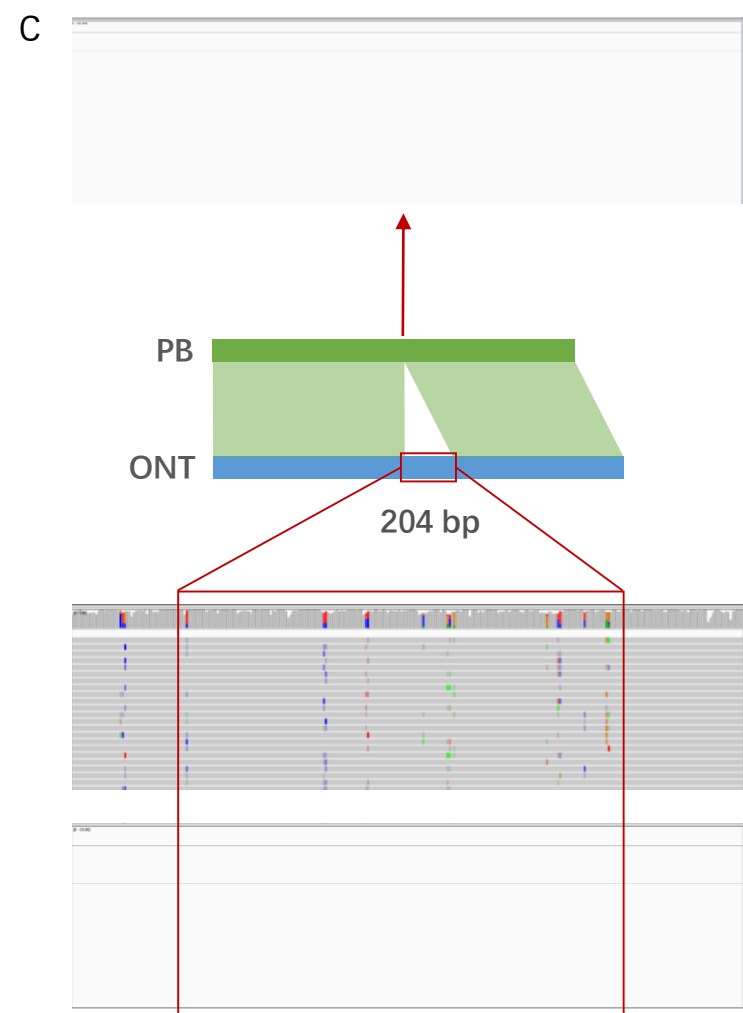

Supplement: giaa123_Supplemental_Files [file giaa123_supplemental_files.zip › Figure S11.pdf]

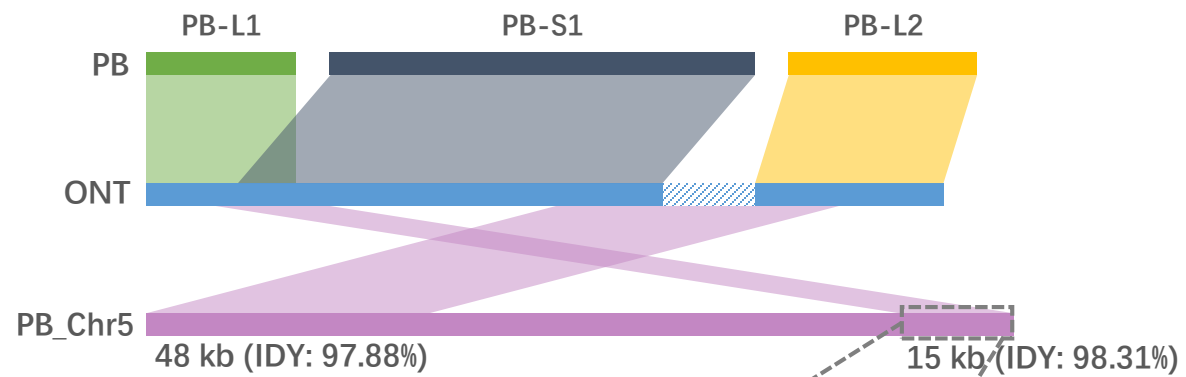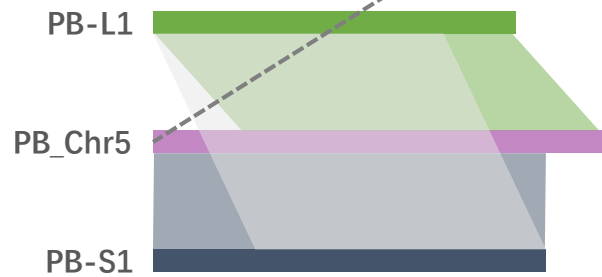

Supplement: giaa123_Supplemental_Files [file giaa123_supplemental_files.zip › Figure S3.pdf]

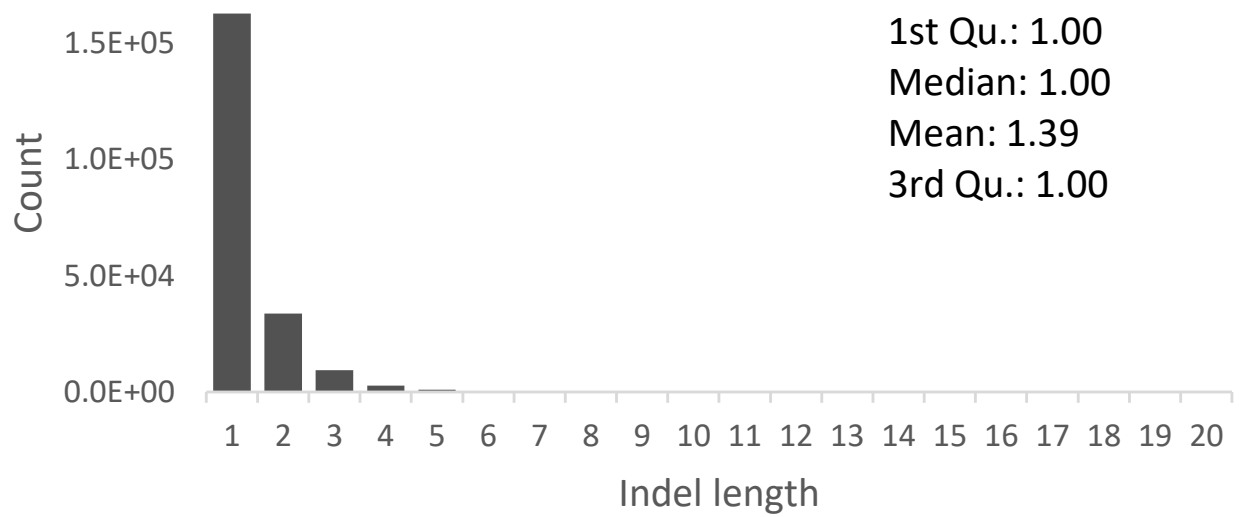

Supplement: giaa123_Supplemental_Files [file giaa123_supplemental_files.zip › Figure S5.pdf]

a.

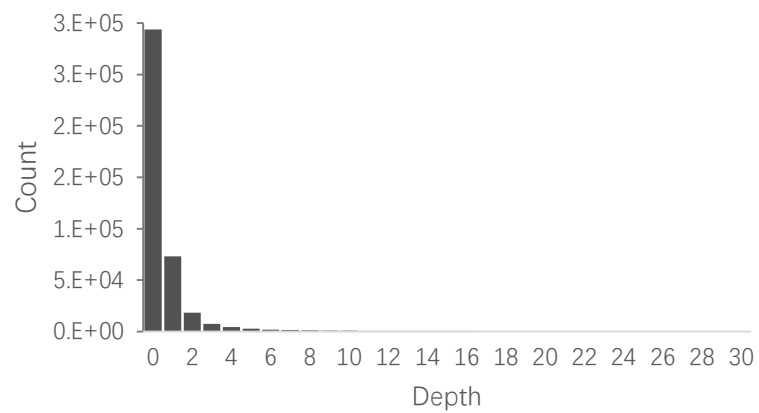

b.

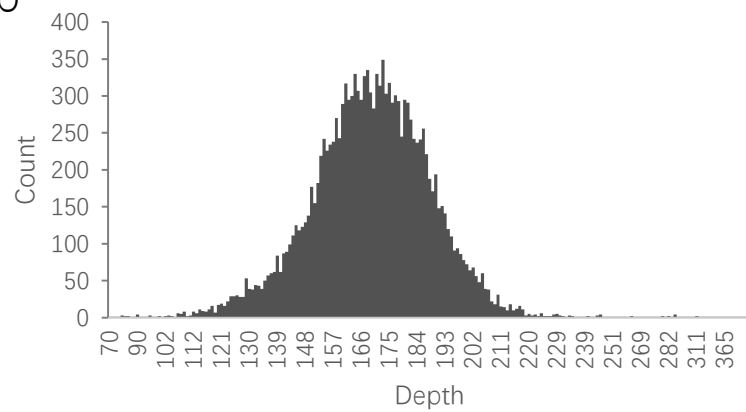

c.

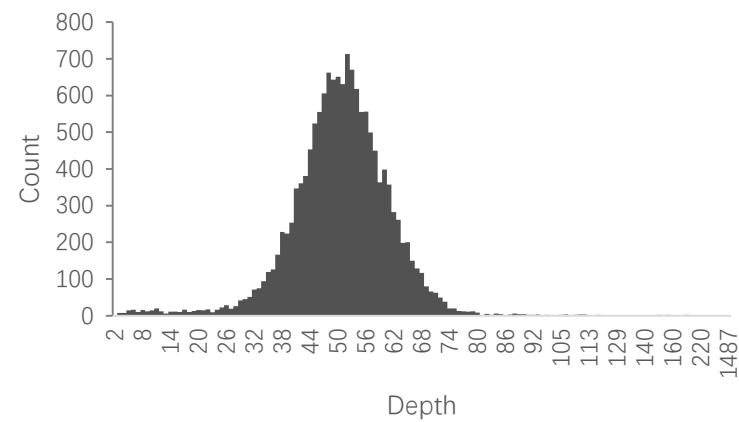

Supplement: giaa123_Supplemental_Files [file giaa123_supplemental_files.zip › Figure S7.pdf]

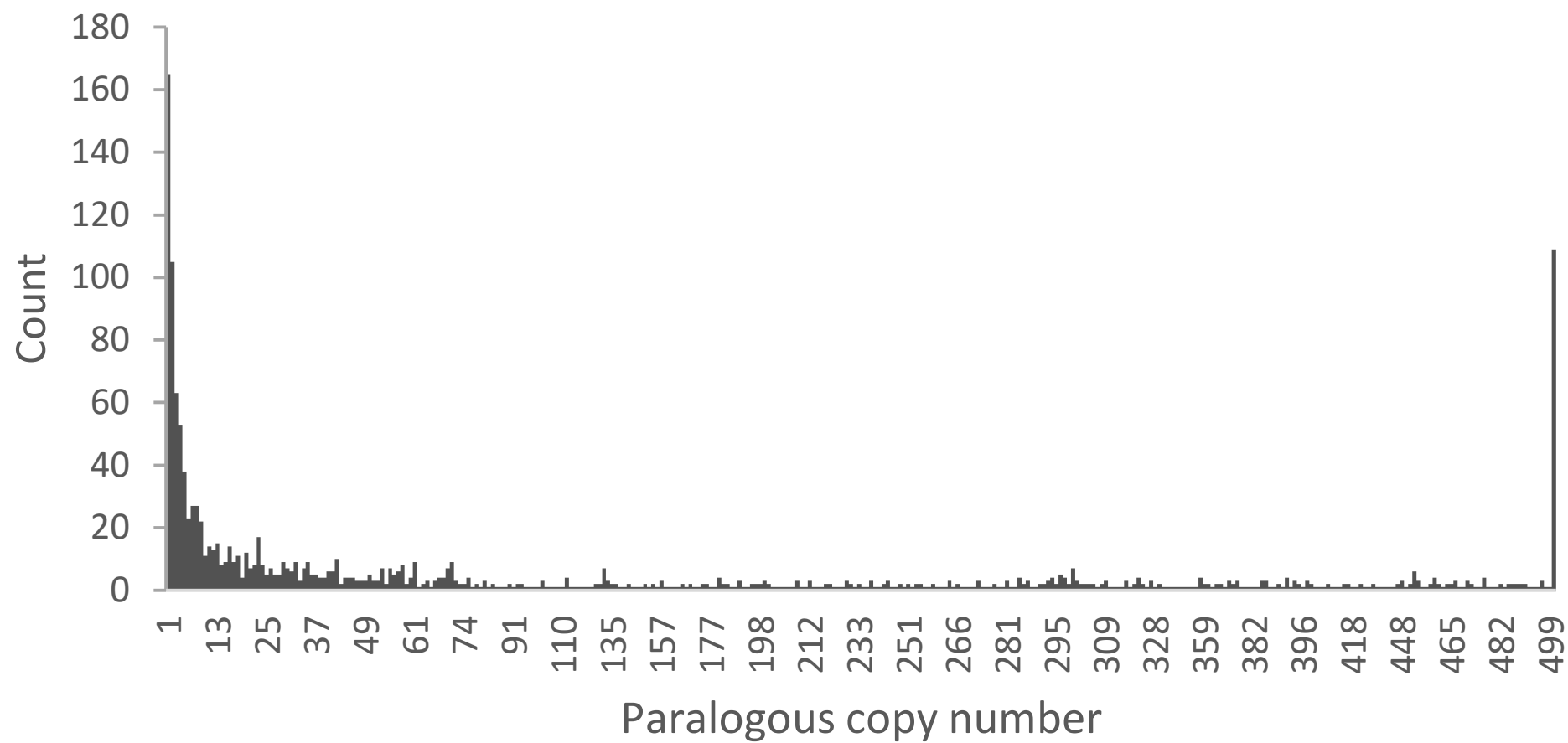

Supplement: giaa123_Supplemental_Files [file giaa123_supplemental_files.zip › Figure S9.pdf]
