# Supplementary material for: Comparison of the two up-to-date sequencing technologies for genome assembly: HiFi reads of Pacific Biosciences Sequel II system and ultralong reads of Oxford Nanopore
Source: Gigascience. 2020 Dec 15;9(12):giaa123. doi: 10.1093/gigascience/giaa123 (PMC7736813; doi:10.1093/gigascience/giaa123)
Supplement: giaa123_Response_to_Reviewer_Comments_Revision_1 [file giaa123_response_to_reviewer_comments_revision_1.pdf]

Author's Response To Reviewer Comments

Close

Dear Editor,

Thank you for handling the review of our manuscript. We appreciate your rapid feedback and the constructive reviews from the editorial board members and the reviewers. We have comprehensively addressed this feedback in our response below. We hope that this version of our manuscript is now suitable for publication in GigaScience.

Sincerely

Shanlin Liu

Comments from the Editorial Board Members:

1) Reviewer 2 in particular agrees with reviewer 3 that a direct comparison of similar methods would have been preferred - please discuss this in the manuscript.

>>> Thank you for your suggestion. We agreed that readers could be interested in not only the N50 value of the assemblies generated by different software, but also the assembly accuracy. In the revised version, we added the assembly accuracy estimations for all the assemblies. As a result, it now includes the comparisons between the assemblies generated using same software and analysis pipeline (Lines 169-174 and Figure 3).

2) Also the use of a new assembly method is problematic, as it is not well known in the field. I understand that validating this new method is outside the scope of your paper, but I recommend you mention this also as a limitation in the manuscript.

>>> We added this limitation at lines 183-189. It reads "However, the current study has several limitations, including, among others, (1) NextDenovo which generated the most contiguous assembly for the ONT is a newly developed assembler that has not been validated its performance on other species; (2) the rice which has a relatively small and simple genome cannot characterize the full spectrum of the strength and weakness of the two sequencing technologies. Genome studies, especially for those large and complex genomes, will shed more light on this matter.". Furthermore, we noted that the developer of NextDenovo have updated their Github page which now includes its performance benchmarking to several widely-used assemblers, such as Canu, Flye, et al., using human genome.

3) I recommend that you also briefly discuss the concern that, being a case study in rice, the results may not be readily applicable to other species, as each species has its own challenges.

>>> Agree. Please find the above response #2.

Please also address the other latest comments of reviewers 1 and 2 in a second revised manuscript. (I note that reviewer 2 could not access the FTP for supporting data- not quite sure where the problem is, as it seems to be working at my end ... our data curators can help the reviewer, if needed).

>>> It will be great that you can help the reviewer #2 to get the data on the FTP in the case that he/she fails to access the NCBI data as well.

Comments from the Reviewers:

Reviewer #1: Thanks for address most of the points I raised. The revised manuscript is a good improvment. Thanks. One minor thing, I am not sure the term "one suite of a diploid genome" is the

right way to describe one single haplotype of the homologous chromosomes, please consider to the revise that for the manuscript.

>>> Thank you for your suggestion. We changed it to “one set of the paired chromosomes” at line 248 according to your advice.

Reviewer #2: The authors have addressed the concerns I raised in my first review. However, I agree with reviewer#3 on numerous points and the authors responses do raise more questions than they answer.

The authors state:
"It is weird that the reviewer argued about the reliability of its assembly results because it generated a much better results compared to the other software. It is worth noting that its readme text on github states that it performs well especially for ONT ultra-long reads."

Reviewer#3 is saying that there is no information on this assembler and relying on N50 is not a good gauge of whether the assembler is doing a good job. Also, it doesn't really matter what the readme states on github. Until a technology is proven to work, and in this case work well with ONT data, it is impossible to judge without evidence.

These comments also exposed an aspect of the paper which could be improved. The authors are arguing they are trying to make a dataset that will inform researchers how to leverage sequencing platforms for a specific goal. However, the analysis is not parallel in the sense that the authors don't compare similar assembly and polishing methods. It is great that the authors added the results from other assemblers. What would be even better is if the analysis was augmented to compare each of those assemblies. At the very least the main comparison should use the same assembly method.

>>> Thank you for your reminding. We realized that the good performance of this new assembly method (NextDenovo) for rice cannot prove that it can give equivalent performances to other species as well, and this might be a big flaw of the current study. Therefore, we firstly included some additional discussions to expose the limitations of the current work, and also included the comparisons that used the same assembly method. Please find our response #1 and #2 to the editorial board members.

Reveiwer#3 also made several other good points that the authors should take more care in addressing.

The methylation addition was a highlight. Since the technologies are moving so fast, and this manuscript is really about technology, have the authors tried the new methylation aware base-calling for ONT? Since so many of the base calling errors in ONT are due to modified bases at this point, it seems very important for the authors to present the most up to date analysis.

>>> We used the latest official release software GUPPY for basecalling, in which we failed to find any parameters specific for methylation. However, as far as we know, the performance of any particular ONT basecaller is influenced by the data used to train its model. Therefore, basecalling for native DNA (not PCR products) can perform much better in the case that their modifications and sequence motifs are represented in its training set compared to that not [1]. Inclusion of a species-specific training set for rice is feasible and will benefit the assembly accuracy for the ONT assemblies, which, however, violating our initial purpose of this study. Because most species cannot achieve such a training set as they do not have genome sequences that are publicly available, and will make the current work an unfair comparison. We added this alternative solution at lines 151-153. It reads “Providing a training set that includes information of modifications and sequence motifs of rice could at some extent alleviate the error rate of the ONT assembly.”.

Thank you for including the FTP. After several tries on different days, I could not download the full assemblies to validate the claims.

>>> Please find our response #4 to the editorial board members.

Minor edits
These are not assembly errors, they are SNPs/INDELs resulting from mis-called bases.
138 "identify assembly errors under the assumption that HiFi reads provide high-level…"

>>> Corrected.

"suggesting" would be more accurate then revealing since
134 "revealing a limited performance of short-reads-based genome polishing methods for"

>>> Corrected.

Reword "by PB, or regions with high methylation level where ONT errors enriched", PB is not an assembler
"discrepancies on Chr. 6 showed that they were repeated regions incorrectly assembled by PB, or regions with high methylation level where ONT errors enriched (Supplementary Methods and Figure S11)."

>>> We corrected it to "using PB reads".

Reference

1. Wick RR, Judd LM, Holt KE. Performance of neural network basecalling tools for Oxford Nanopore sequencing. Genome Biol. 2019;20:129.

Close
