# Supplementary material for: Comparison of the two up-to-date sequencing technologies for genome assembly: HiFi reads of Pacific Biosciences Sequel II system and ultralong reads of Oxford Nanopore
Source: Gigascience. 2020 Dec 15;9(12):giaa123. doi: 10.1093/gigascience/giaa123 (PMC7736813; doi:10.1093/gigascience/giaa123)
Supplement: giaa123_Reviewer_1_Report_Original_Submission — Jason Chin -- 3/22/2020 Reviewed [file giaa123_reviewer_1_report_original_submission.pdf]

Reviewer Report

Title: Comparison of the two up-to-date sequencing technologies for genome assembly: HiFi reads of Pacbio Sequel II system and ultralong reads of Oxford Nanopore

Version: Original Submission **Date:** 3/22/2020

Reviewer name: Jason Chin

Reviewer Comments to Author:

This manuscript compares the results of genome assemblies from the data of two long-reads sequencing technologies and multiple genome assemblers. It focuses on analyzing the impact of the sequence qualities (read lengths and accuracies) to the contiguity and the accuracy of the assembled contigs. While the results agree with the general understanding of how the read lengths and the basecall accuracies affect the final assembly quality, I found the detailed examples comparing the two picked assemblies are interesting. It provides useful insight for understanding the impact of repeats for genome assembly results for researchers. The manuscript is well written and easy to follow to get the points across. Here are a couple points that I hope the authors will be able to address:

(1) While the rice strain is documented in the manuscript, it will be useful to comment on the polyploidy of this particular strain? The BUSCO results seem to indicate it is a haploid strain, and the readers may be able to check it out from the strain ID. However, the authors should comment on the polyploid to help the readers. It is important to understand how to interpret results according to the known polyploidy.

(2) In the paragraph starting with "Following DNA extraction", please refer to the supplementary material about the extraction protocol there.

(3) The authors should comment on the time used for sequencing on PromethION and Sequel II, and the computation resources (CPU/wall clock time, memory, cluster setup, etc.) needed for each assembler.

(4) The IGV view of the ONT reads mapped the PacBio assembly GAP doest show the disagreement of the ONT reads to the ONT contigs. While the high error rates may it messy to see. If such a view hard to see, it still useful to examine if there is some systematic disagreement between the reads and the contigs. I am hoping the authors can comment on whether some systematic errors are visible. Also, will it provide useful insight if we compare it to PacBio Reads mapping to the ONT contigs?

(5) When the authors refer to "string graph," it needs a citation. The term the "string graph" is coined by Gene Meyer for a specific way to construct a graph for genome assembly. Not all assemblers use the same graph construction. The authors should use "assembly graphs" and cite related papers.

(6) Related to the polyploidy of the strain, the author mentioned "diploid heterozygous states," there is no citation or explanation to help the readers to know what the authors refer to.

(7) The authors mention the errors in ONT assembly are clustered. The authors' explanation is because of low coverage mapping in the polish steps. Are these clusters caused by repeat contents, low accuracy of ONT assembly op particular sequencing contexts? In the caption of Figure S5, the authors write: "the distances should have a peak around 1,000 bp for an average error rate of 1.06 per kb in the

case of random distribution." The author should put a theoretical curve or a simulated one on the same plot to show the distribution of a random error model does generate a different distribution.

Level of Interest

Please indicate how interesting you found the manuscript: Choose an item.

Quality of Written English

Please indicate the quality of language in the manuscript: Choose an item.

Declaration of Competing Interests

Please complete a declaration of competing interests, considering the following questions:

- Have you in the past five years received reimbursements, fees, funding, or salary from an organisation that may in any way gain or lose financially from the publication of this manuscript, either now or in the future?
- Do you hold any stocks or shares in an organisation that may in any way gain or lose financially from the publication of this manuscript, either now or in the future?
- Do you hold or are you currently applying for any patents relating to the content of the manuscript?
- Have you received reimbursements, fees, funding, or salary from an organization that holds or has applied for patents relating to the content of the manuscript?
- Do you have any other financial competing interests?
- Do you have any non-financial competing interests in relation to this paper?

If you can answer no to all of the above, write 'I declare that I have no competing interests' below. If your reply is yes to any, please give details below.

1. Yes, I was an employee of PacBio. 2. Yes, I am a share holder of PacBio 3. No 4. No 5. No 6. No

I agree to the open peer review policy of the journal. I understand that my name will be included on my report to the authors and, if the manuscript is accepted for publication, my named report including any attachments I upload will be posted on the website along with the authors' responses. I agree for my report to be made available under an Open Access Creative Commons CC-BY license (http://creativecommons.org/licenses/by/4.0/). I understand that any comments which I do not wish to be included in my named report can be included as confidential comments to the editors, which will not be published.

Choose an item.

To further support our reviewers, we have joined with Publons, where you can gain additional credit to further highlight your hard work (see: https://publons.com/journal/530/gigascience). On publication of this paper, your review will be automatically added to Publons, you can then choose whether or not to claim your Publons credit. I understand this statement.

Yes Choose an item.
