# Supplementary material for: Comparison of the two up-to-date sequencing technologies for genome assembly: HiFi reads of Pacific Biosciences Sequel II system and ultralong reads of Oxford Nanopore
Source: Gigascience. 2020 Dec 15;9(12):giaa123. doi: 10.1093/gigascience/giaa123 (PMC7736813; doi:10.1093/gigascience/giaa123)
Supplement: giaa123_Reviewer_2_Report_Original_Submission — Todd Michael -- 4/3/2020 Reviewed [file giaa123_reviewer_2_report_original_submission.pdf]

Reviewer Report

Title: Comparison of the two up-to-date sequencing technologies for genome assembly: HiFi reads of Pacbio Sequel II system and ultralong reads of Oxford Nanopore

Version: Original Submission **Date:** 4/3/2020

Reviewer name: Todd Michael

Reviewer Comments to Author:

In the manuscript entitled, "Comparison of the two up-to-date sequencing technologies for genome assembly: HiFi reads of PacBio Sequel II and ultralong reads of Oxford Nanopore," Lang et al., generate assemblies for a rice variety (9311) using the two different long read sequencing technologies and then compare contiguity and accuracy statistics. The authors conclude that Oxford Nanopore Technologies (ONT) sequencing provides superior contiguity, while Pacific Bioscience (PacBio) provides superior base quality accuracy, and that the two platforms should be leveraged together for reference quality genomes. Overall the manuscript is very concise and well developed. However, there are a couple points that the authors should acknowledge and discuss, which impact the interpretation of their results.
First off, the BioProject PRJNA600693 was not available to assess the assemblies or the raw data. In a manuscript that compares genomes, validating some of the claims is essential, and the data should be available to the reviewer.
The authors set up a very nice and simple contrast between PacBio HiFi and ONT. There are some significant differences between the datasets that should be discussed though. The read N50 length of the two platforms is considerably different at 41 kb vs. 13 kb for ONT and PacBio respectively. Moreover the absolute coverage is significantly different between ONT and PacBio at 92 Gb (230x) vs. 253 Gb (632x) respectively, even though the reported HiFi coverage is only 50X. There are several opportunities here. First, the authors should at the very least mention these differences, which at face value explain ONT being more contiguous and PacBio having higher base quality. Second, since the authors have an extraordinary amount of data for this rice line, it would be also interesting to see where the quality or contiguity starts to decrease as a function of the amount or type of data.
The section about the nucleotide variation is a little confusing. It is stated that the regions (~94%) that showed low base quality in the ONT assembly also had low shotgun read coverage. Was this ONT, PacBio or Illumina coverage that was low? With the amount of coverage that was generated for each platform (ONT, 230x; PacBio, 632x; Illumina 70x) why would there be regions in the assembly with less than 5x coverage. This needs to be clearer. In the same section, SNPs and INDELs are referred to as small-scale mis-assemblies; more accurately these are sequence errors not mis-assemblies. Did the authors use the ONT or PacBio data to look at DNA methylation? If the errors are clustering in the genome then maybe the errors in the ONT sequence are the result of mis-called bases that are highly methylated. Since the data is available this would be an important point to make or reason to rule out.
PacBio can also run in long read mode, so researchers could mix HiFi with longreads on one platform. This would be good to also mention.
The BUSCO scores for the two genomes are almost identical. It would be good to add a bit of

commentary why you see similar BUSCO scores but some differences in protein content. This will help the reader understand the differences and limitations of each measure.
While mentioning exact costs for both methods would not stand the test of time it would be good for the reader to understand the relative cost differences between the two approaches.
Minor points
What species of rice is 9311? The authors should use the scientific name somewhere in the manuscript to clarify what species is "rice."
Grammar
The first sentence of the Main Text. Diseases don't find causative alleles. Maybe, "The human reference genome enabled the identification of disease causative alleles…."
Sentence 4 page 3: species don't leverage cutting edge sequencing. "The two cutting edge sequencing technologies has enabled the sequencing of many species…"
Bottom page 4 "It was gone through by multiple ONT long reads…" It was spanned by….

Level of Interest

Please indicate how interesting you found the manuscript: Choose an item.

Quality of Written English

Please indicate the quality of language in the manuscript: Choose an item.

Declaration of Competing Interests

Please complete a declaration of competing interests, considering the following questions:

- Have you in the past five years received reimbursements, fees, funding, or salary from an organisation that may in any way gain or lose financially from the publication of this manuscript, either now or in the future?
- Do you hold any stocks or shares in an organisation that may in any way gain or lose financially from the publication of this manuscript, either now or in the future?
- Do you hold or are you currently applying for any patents relating to the content of the manuscript?
- Have you received reimbursements, fees, funding, or salary from an organization that holds or has applied for patents relating to the content of the manuscript?
- Do you have any other financial competing interests?
- Do you have any non-financial competing interests in relation to this paper?

If you can answer no to all of the above, write 'I declare that I have no competing interests' below. If your reply is yes to any, please give details below.

I declare that I have no competing interests

I agree to the open peer review policy of the journal. I understand that my name will be included on my report to the authors and, if the manuscript is accepted for publication, my named report including any attachments I upload will be posted on the website along with the authors' responses. I agree for my report to be made available under an Open Access Creative Commons CC-BY license (http://creativecommons.org/licenses/by/4.0/). I understand that any comments which I do not wish to be included in my named report can be included as confidential comments to the editors, which will not be published.

Choose an item.

To further support our reviewers, we have joined with Publons, where you can gain additional credit to further highlight your hard work (see: https://publons.com/journal/530/gigascience). On publication of this paper, your review will be automatically added to Publons, you can then choose whether or not to claim your Publons credit. I understand this statement.

Yes Choose an item.
