# Supplementary material for: Comparison of the two up-to-date sequencing technologies for genome assembly: HiFi reads of Pacific Biosciences Sequel II system and ultralong reads of Oxford Nanopore
Source: Gigascience. 2020 Dec 15;9(12):giaa123. doi: 10.1093/gigascience/giaa123 (PMC7736813; doi:10.1093/gigascience/giaa123)
Supplement: giaa123_Reviewer_2_Report_Revision_1 — Todd Michael -- 7/17/2020 Reviewed [file giaa123_reviewer_2_report_revision_1.pdf]

Reviewer Report

Title: Comparison of the two up-to-date sequencing technologies for genome assembly: HiFi reads of Pacbio Sequel II system and ultralong reads of Oxford Nanopore

Version: Revision 1 **Date:** 7/17/2020

Reviewer name: Todd Michael

Reviewer Comments to Author:

The authors have addressed the concerns I raised in my first review. However, I agree with reviewer#3 on numerous points and the authors responses do raise more questions than they answer.
The authors state:
"It is weird that the reviewer argued about the reliability of its assembly results because it generated a much better results compared to the other software. It is worth noting that its readme text on github states that it performs well especially for ONT ultra-long reads."
Reviewer#3 is saying that there is no information on this assembler and relying on N50 is not a good gauge of whether the assembler is doing a good job. Also, it doesn't really matter what the readme states on github. Until a technology is proven to work, and in this case work well with ONT data, it is impossible to judge without evidence.
These comments also exposed an aspect of the paper which could be improved. The authors are arguing they are trying to make a dataset that will inform researchers how to leverage sequencing platforms for a specific goal. However, the analysis is not parallel in the sense that the authors don't compare similar assembly and polishing methods. It is great that the authors added the results from other assemblers. What would be even better is if the analysis was augmented to compare each of those assemblies. At the very least the main comparison should use the same assembly method.
Reveiwer#3 also made several other good points that the authors should take more care in addressing.
The methylation addition was a highlight. Since the technologies are moving so fast, and this manuscript is really about technology, have the authors tried the new methylation aware base-calling for ONT? Since so many of the base calling errors in ONT are due to modified bases at this point, it seems very important for the authors to present the most up to date analysis.
Thank you for including the FTP. After several tries on different days, I could not download the full assemblies to validate the claims.
Minor edits
These are not assembly errors, they are SNPs/INDELs resulting from mis-called bases.
138 "identify assembly errors under the assumption that HiFi reads provide high-level…"
"suggesting" would be more accurate then revealing since
134 "revealing a limited performance of short-reads-based genome polishing methods for"
reword
164 "by PB, or regions with high methylation level where ONT errors enriched"
PB is not an assembler
162 "discrepancies on Chr. 6 showed that they were repeated regions incorrectly assembled by PB, or

regions with high methylation level where ONT errors enriched (Supplementary Methods and Figure S11)."

Level of Interest

Please indicate how interesting you found the manuscript: Choose an item.

Quality of Written English

Please indicate the quality of language in the manuscript: Choose an item.

Declaration of Competing Interests

Please complete a declaration of competing interests, considering the following questions:

- Have you in the past five years received reimbursements, fees, funding, or salary from an organisation that may in any way gain or lose financially from the publication of this manuscript, either now or in the future?
- Do you hold any stocks or shares in an organisation that may in any way gain or lose financially from the publication of this manuscript, either now or in the future?
- Do you hold or are you currently applying for any patents relating to the content of the manuscript?
- Have you received reimbursements, fees, funding, or salary from an organization that holds or has applied for patents relating to the content of the manuscript?
- Do you have any other financial competing interests?
- Do you have any non-financial competing interests in relation to this paper?

If you can answer no to all of the above, write 'I declare that I have no competing interests' below. If your reply is yes to any, please give details below.

I declare that I have no competing interests

I agree to the open peer review policy of the journal. I understand that my name will be included on my report to the authors and, if the manuscript is accepted for publication, my named report including any attachments I upload will be posted on the website along with the authors' responses. I agree for my report to be made available under an Open Access Creative Commons CC-BY license (http://creativecommons.org/licenses/by/4.0/). I understand that any comments which I do not wish to be included in my named report can be included as confidential comments to the editors, which will not be published.

Choose an item.

To further support our reviewers, we have joined with Publons, where you can gain additional credit to further highlight your hard work (see: https://publons.com/journal/530/gigascience). On publication of this paper, your review will be automatically added to Publons, you can then choose whether or not to claim your Publons credit. I understand this statement.

Yes Choose an item.
