# Supplementary material for: Comparison of the two up-to-date sequencing technologies for genome assembly: HiFi reads of Pacific Biosciences Sequel II system and ultralong reads of Oxford Nanopore
Source: Gigascience. 2020 Dec 15;9(12):giaa123. doi: 10.1093/gigascience/giaa123 (PMC7736813; doi:10.1093/gigascience/giaa123)
Supplement: giaa123_Reviewer_3_Report_Original_Submission — Sergey Nurk -- 4/20/2020 Reviewed [file giaa123_reviewer_3_report_original_submission.pdf]

Reviewer Report

Title: Comparison of the two up-to-date sequencing technologies for genome assembly: HiFi reads of Pacbio Sequel II system and ultralong reads of Oxford Nanopore

Version: Original Submission **Date:** 4/20/2020

Reviewer name: Sergey Nurk

Reviewer Comments to Author:

Advances in sequencing technologies provide us with an unprecedented opportunity for high-quality de novo reconstruction of complex eukaryotic genomes.
The manuscript presents the comparative analysis of the two assemblies of a rice genome, obtained with ultra-long ONT and Pacbio HiFi sequencing.
First, while a combination of HiFi and ultra-long ONT datasets is available for several human genomes (and maybe some other organisms), the scope of the study is limited to a single organism with a relatively small and simple genome. Moreover, only a single genome has been considered with a single dataset for each technology. In particular, while longer Pacbio HiFi libraries with reads reaching 20Kb are now not uncommon the dataset considered in the study had an average read length less than 12Kb.
Further I will focus on major issues of the presented analysis and mention some of the minor ones in the end.
Major issues
The 'primary' ONT assembly used was produced by a software tool for which I was not able to find neither publication/white-paper, nor a comprehensive benchmark. Moreover its github page states "In addition, we found that NextDenovo, of the current version, might produce a small number of unexpected connection errors in the highly repetitive regions, which, however, can be easily corrected using additional Hi-C or Bionano data. We are still in a progress of optimizing NextDenovo and will continuously update it, especially in terms of assembly accuracy". Since the only criteria used to choose the 'optimal' assembly between different assembly tools was based on their N50 values, it immediately raises questions about the reliability of the results!
The only confirmation of assembly accuracy given is the dotplot against the reference genome. Unfortunately at the presented resolution (of both the figure and the analysis itself) it fails to convince the reader of the structural accuracy of the assembly.
Also the discrepancy between N50 values of different ONT assemblies looks staggering and raises suspicion. I would suggest to include stats for some other well established long-read assemblers (e.g. Flye and Shasta), which will hopefully be able to produce assembly with continuity comparable to NextDenovo and dispel the suspicion.
As a side note, somehow the main text never states which assemblies were used for the most part of the analysis.
One of the most surprising points of the analysis is that the authors insist on interpreting 'redundancies' as 'misassemblies', which is not a common practice in the assembly benchmarking. While it is important to highlight that while dealing with diploid genomes one can expect to get higher redundancy from HiFi-

based assemblies, which should hardly be considered an error as long as they truly represent one of the haplotypes. Besides heterozygous differences, another potential source of redundancies can come from the fact that most long-read assemblers produce overlapping contigs, so the higher the number of fragments the higher will be 'redundancy' from those overlaps. Overall, I don't think that any types of redundancies should be considered as a serious problem at the assembly side. If needed, both types of common redundancies described above can be more-or-less straightforwardly removed post assembly (e.g. purge_dups software), but most importantly they stem primarily from particular algorithm implementation rather than show a deficiency of a data type. For example I would expect Flye's assembly of HiFi data to get much lower redundancy values due to more aggressive settings toward masking heterozygous differences and output of 'bluntified' contigs. Last but not least, from the methodological point of view, while I'm still uncertain how 'redundant' regions were annotated, they have been certainly detected against the draft ONT assembly, which could contain 'collapsed' tandem repeats and other issues, potentially inflating the stats.

Significant part of the main text focuses on the analysis of a handful of particular cases of contig 'breaks' in HiFi assembly. First, the choice of 3 gaps taken for deeper analysis (corresponding to chr6) is not explained and, considering how few of them are described, it is unclear how well they represent the general situation. Second, at least some of the analysis is questionable. For one of the gaps the manuscript states that "... the overlapping and the gap regions represented two elements of 15 kb and 48 kb in length that, although have only one copy on Chr. 6, can find their duplications on Chr. 5 (Figure S3). Repetitive elements with such lengths go beyond the typical length generated by PB CCS, therefore the right path can hardly be disentangled from complicated string graphs." At the same time on Figure S3 the sequence identity for instances of both repeats is reported below 98.5%! Repeat instances of such a high sequence divergence are extremely unlikely to affect HiCanu results, so there must be some other reason for fragmentation of this region.

I would recommend exploring the mapping of the HiFi reads onto the hypothesized genomic sequence, since it has been recently observed that HiFi reads can exhibit depletion of coverage in the GA-rich microsatellite regions of the genome. Besides being responsible for some of the observed gaps in this particular assembly, deeper investigation of this topic could have a serious impact on the choice of technology for certain assembly projects.

As a final major note I would like to highlight that the data used in the study doesn't seem to be available yet (query of the PRJNA600693 id doesn't return any results on NCBI web site). TODO review was hampered by this.

Minor issues.

If I understood correctly, the coverage of HiFi data exceeded 500x (253 Gb of data for a roughly 400Mb genome). Since it far exceeds the typical coverage of sequencing projects that most assemblers (e.g. HiCanu) are tuned to, I would suggest to subsample HiFi data or use HiCanu 2.0 (which would perform subsampling automatically) for processing a dataset of such coverage depth.

The authors note that "the errors of HiFi reads may be enriched in sequences with particular characteristics, rather than completely random … which may exacerbate the above error statistics for the ONT assembly", suggesting that the rate of the indels in polished ONT assemblies can be noticeably overestimated. I doubt that it is the case though. While the same properties of individual HiFi reads have also been recently observed by other investigators, to the best of my knowledge the consensus quality

still tends to be very high. At the same time, the authors can make a much stronger claim by straightforwardly estimating the rate of 'false positive' errors detected within the regions of high coverage of unambiguously mapped Illumina reads.
The statement "PB assembly contained more gaps in each chromosome compared to that of ONT" can not be correct, since before that authors say that there were 3 cromosomes fully assembled from HiFi data.
I would suggest against direct attempts at polishing HiFi assemblies with Racon, since it might result in corrupting the correctly assembled sequence within repetitive regions.
Conclusion.
Expectedly, while less than 60 genes were affected by identified assembly problems in HiFi assembly (most by redundancies, which as I mentioned before for the most part are easy to mitigate), even after polishing with Illumina reads > 1000 genes were affected by indels in the reported ONT assembly.
Setting aside all the above mentioned issues, the results suggest the conclusion that ultra-long ONT could work well for scaffolding HiFi-based assemblies in order to produce almost-perfect genomic reconstruction of inbred rice varieties.
Overall, the presented manuscript falls short of providing the comprehensive comparison of the two technologies for sequence assembly (which a reader expects from its title), but works as a case study of how their combination should be able to provide an almost perfect medium-complexity genome of low-heterozygosity.
Last but not least I found some parts of the manuscript quite poorly written. Additional rounds of revisions are highly recommended before resubmission.

Level of Interest

Please indicate how interesting you found the manuscript: Choose an item.

Quality of Written English

Please indicate the quality of language in the manuscript: Choose an item.

Declaration of Competing Interests

Please complete a declaration of competing interests, considering the following questions:

- Have you in the past five years received reimbursements, fees, funding, or salary from an organisation that may in any way gain or lose financially from the publication of this manuscript, either now or in the future?
- Do you hold any stocks or shares in an organisation that may in any way gain or lose financially from the publication of this manuscript, either now or in the future?
- Do you hold or are you currently applying for any patents relating to the content of the manuscript?

- Have you received reimbursements, fees, funding, or salary from an organization that holds or has applied for patents relating to the content of the manuscript?
- Do you have any other financial competing interests?
- Do you have any non-financial competing interests in relation to this paper?

If you can answer no to all of the above, write 'I declare that I have no competing interests' below. If your reply is yes to any, please give details below.

I am one of the authors of the HiCanu software, which is benchmarked by the authors. While I did my best to provide an unbiased evaluation, this could be viewed as a conflict of interest.

I agree to the open peer review policy of the journal. I understand that my name will be included on my report to the authors and, if the manuscript is accepted for publication, my named report including any attachments I upload will be posted on the website along with the authors' responses. I agree for my report to be made available under an Open Access Creative Commons CC-BY license (http://creativecommons.org/licenses/by/4.0/). I understand that any comments which I do not wish to be included in my named report can be included as confidential comments to the editors, which will not be published.

Choose an item.

To further support our reviewers, we have joined with Publons, where you can gain additional credit to further highlight your hard work (see: https://publons.com/journal/530/gigascience). On publication of this paper, your review will be automatically added to Publons, you can then choose whether or not to claim your Publons credit. I understand this statement.

Yes Choose an item.
